# BaPreS: a software tool for predicting bacteriocins using an optimal set of features

**DOI:** 10.1186/s12859-023-05330-z

**Published:** 2023-08-17

**Authors:** Suraiya Akhter, John H. Miller

**Affiliations:** 1 School of Electrical Engineering and Computer Science, Washington State University, Pullman, WA USA; 2 School of Engineering and Applied Sciences, Washington State University Tri-Cities, Richland, WA USA

**Keywords:** Antibiotic resistance, Bacteriocin prediction, Feature selection, Machine learning, Deep learning, Sequence matching

## Abstract

**Background:**

Antibiotic resistance is a major public health concern around the globe. As a result, researchers always look for new compounds to develop new antibiotic drugs for combating antibiotic-resistant bacteria. Bacteriocin becomes a promising antimicrobial agent to fight against antibiotic resistance, due to cases of both broad and narrow killing spectra. Sequence matching methods are widely used to identify bacteriocins by comparing them with the known bacteriocin sequences; however, these methods often fail to detect new bacteriocin sequences due to their high diversity. The ability to use a machine learning approach can help find new highly dissimilar bacteriocins for developing highly effective antibiotic drugs. The aim of this work is to develop a machine learning-based software tool called BaPreS (Bacteriocin Prediction Software) using an optimal set of features for detecting bacteriocin protein sequences with high accuracy. We extracted potential features from known bacteriocin and non-bacteriocin sequences by considering the physicochemical and structural properties of the protein sequences. Then we reduced the feature set using statistical justifications and recursive feature elimination technique. Finally, we built support vector machine (SVM) and random forest (RF) models using the selected features and utilized the best machine learning model to implement the software tool.

**Results:**

We applied BaPreS to an established dataset and evaluated its prediction performance. Acquired results show that the software tool can achieve a prediction accuracy of 95.54% for testing protein sequences. This tool allows users to add new bacteriocin or non-bacteriocin sequences in the training dataset to further enhance the predictive power of the tool. We compared the prediction performance of the BaPreS with a popular sequence matching-based tool and a deep learning-based method, and our software tool outperformed both.

**Conclusions:**

BaPreS is a bacteriocin prediction tool that can be used to discover new highly dissimilar bacteriocins for developing highly effective antibiotic drugs. This software tool can be used with Windows, Linux and macOS operating systems. The open-source software package and its user manual are available at https://github.com/suraiya14/BaPreS.

**Supplementary Information:**

The online version contains supplementary material available at 10.1186/s12859-023-05330-z.

## Background

Bacteria become antibiotic resistant due to the excessive use of drugs in healthcare and agriculture. In the United States, around 3-million people get infected and approximately 35,000 individuals die because of antibiotic-resistant organisms [1]. Therefore, the resistance nature of bacteria drives the need for inventing novel antimicrobial compounds to treat antibiotic-resistant patients. Researchers developed several approaches to extract natural products as antimicrobial compounds by mining the bacterial genomes [2]. Bacteriocin is one type of natural antimicrobial compound which is a bacterial ribosomal product. As bacteriocins have both broad and narrow killing spectra depending on their specific structure and mode of action, they became attractive choices in the discovery of novel drugs that can produce less resistance in bacteria [3–5]. Current whole genome sequencing technology provides many genes that encode bacteriocins and these sequences are publicly available for future research. Researchers introduced several methods to identify bacteriocins from bacterial genomes based on bacteriocin precursor genes or context genes. For example, BAGEL [6] and BACTIBASE [7] are two publicly available online tools that curate experimentally validated and annotated bacteriocins. Like the widely used protein searching tool BLASTP [8, 9], these methods also allow users to identify putative bacteriocin sequences based on the homogeneity of known bacteriocins. However, these similarity-based approaches often fail to detect useful sequences that have high dissimilarity with known bacteriocin sequences; thereby, generating an undesired number of false negatives. To resolve this problem, some prediction tools, such as BOA (Bacteriocin Operon Associator) [10], were developed based on locating conserved context genes of the bacteriocin operon, but they still rely on homology-based genome searches.

Machine learning technique can be applied as a substitute for sequence similarity and context-based methods that can utilize potential peptide (protein) features of bacteriocin and non-bacteriocin to make strong prediction in identifying novel bacteriocin sequences. Recently some machine learning-based bacteriocin prediction techniques were proposed that utilized the presence or absence of *k*-mer (i.e., subsequences of length *k*) as potential features and represented peptide sequences using word embedding [11, 12]. There are also deep learning-based methods for bacteriocin prediction, for example RMSCNN [13] used a convolutional neural network [14, 15] for identifying marine microbial bacteriocins. However, these existing approaches did not consider the primary and secondary structure information of peptides that are crucial to find highly dissimilar bacteriocins. Also, those strategies did not apply any feature evaluation algorithm to eliminate the unnecessary features that may reduce the achievement of a machine learning classifier.

In this work we present a predictive pipeline for identifying bacteriocins by generating features from the physicochemical and structural characteristics of peptide sequences. We evaluated and selected subsets of the candidate features based on Pearson correlation coefficient, *t* − test, mean decrease Gini (MDG), and recursive feature elimination (RFE) analyses. The reduced feature sets called optimal feature sets are then used to predict bacteriocins using support vector machine (SVM) [16] and random forest (RF) [17] machine learning models. The main objective was to develop a software package called Bacteriocin Prediction Software (BaPreS) using the best machine learning model with a simple and intuitive graphical user interface (GUI) that can generate all required optimal features to get prediction results for testing protein sequences. The software provides options to users to test multiple sequences and add new training bacteriocin or non-bacteriocin sequences to the machine learning model for improving the prediction capability. BLASTP, a sequence matching tool and RMSCNN, a deep learning model were used to compare the performance of our software tool.

### Implementation

**Fig. 1:**
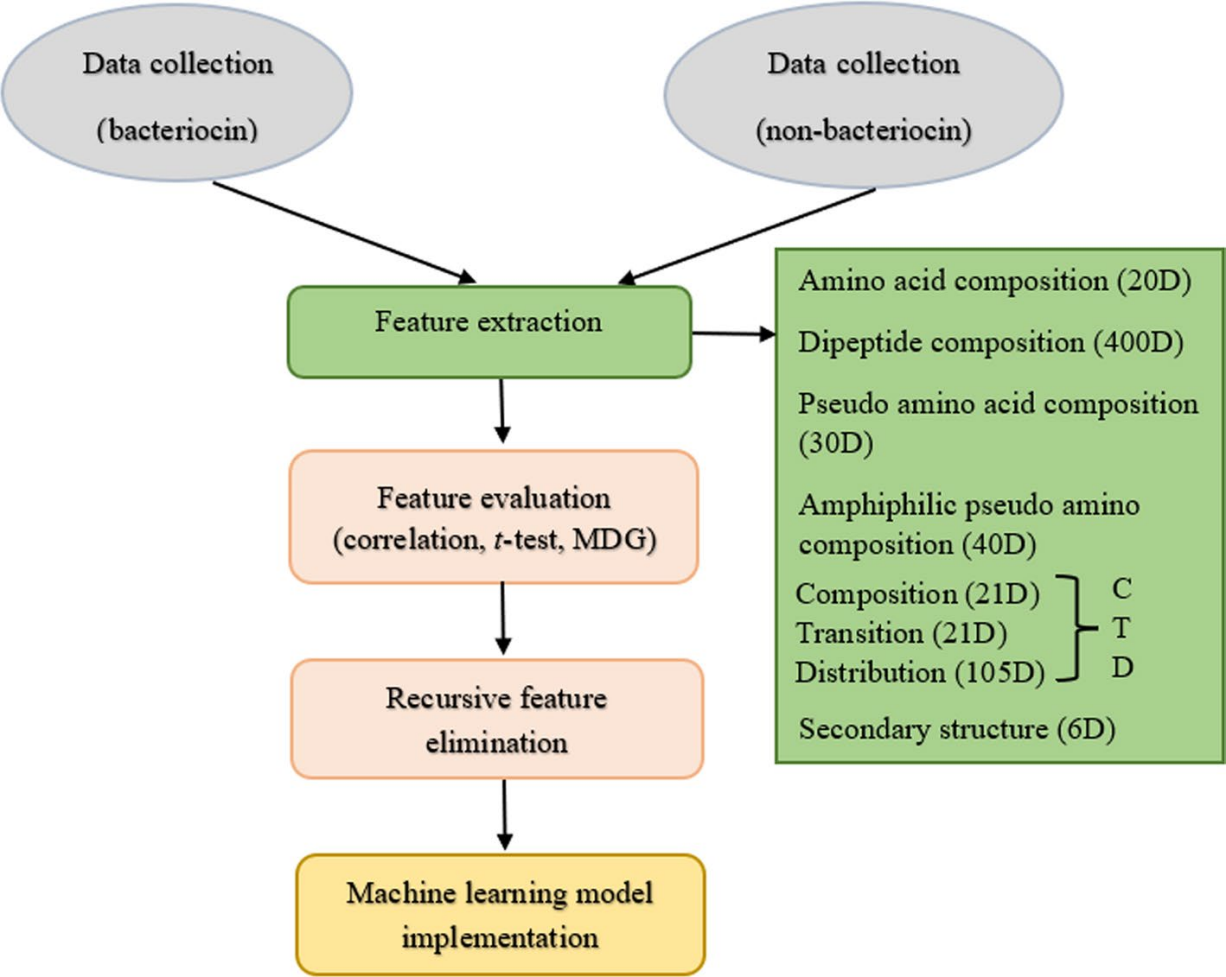
Illustrating the steps involved in the prediction of bacteriocin protein sequence

The overall workflow of our methods is depicted in Fig. 1. The steps in our methods include gathering datasets of bacteriocin and non-bacteriocin protein sequences, generating potential features, performing feature evaluation and recursive feature elimination analyses to remove irrelevant and weakest features, and finally building machine learning models using the selecting features to compare the prediction performance with the sequence matching and deep learning-based approaches.

We retrieved experimentally validated and annotated bacteriocin sequences (positive sequences) from two publicly available databases BAGEL [6] and BACTIBASE [7]. Non-bacteriocin sequences (negative sequences) were collected from the data used in RMSCNN [13]. Initially, we gathered a total of 483 positive and 500 negative sequences. As many of these accumulated sequences are duplicate or of high similarity and a machine learning model can be biased because of these duplicate sequences, we utilized CD-HIT tool [18] to obtain the unique positive and negative sequences by removing the sequences having ≥ 90% similarity. Choosing a lower similarity cutoff in the CD-HIT tool may lessen the sequence homology bias; however, as bacteriocins are a heterogeneous class of bacterial peptides (proteins) and there is a possibility that various novel bacteriocins have not yet been detected, we can consider a threshold of 90% sequence similarity to predict novel bacteriocin sequences [19–22]. Finally, we obtained 283 and 497 unique positive and negative sequences, respectively. To deal with the imbalanced dataset problem, we reduced the negative sequences from 497 to 283 by random sampling to make the number of positive and negative examples equal. We considered 80% and 20% of the total sequences as training and testing datasets, respectively. Positive and negative training sequences, in FASTA format, are listed in Additional file 1. The distribution of the training data to understand the pattern or gain insights into the relationships among the features is depicted in (Additional file 2: Fig. S1). Positive and negative testing sequences are presented in Additional file 3.

After collecting the positive and negative protein sequences, we generated potential candidate features from the sequences. Since there are 20 natural amino acids, we generated a 20D (‘D’ indicates dimension) amino acid composition (AAC) feature vector for every protein sequence where each value in the vector gives the fraction of a specific amino acid type. We extracted 400D dipeptide composition (DC) feature vectors for the sequences where each value indicates the fraction of dipeptides in a protein sequence [23]. Pseudo amino acid composition (PseAAC) and amphiphilic pseudo amino acid composition (APseAAC) feature vectors of 30D and 40D, respectively, were created for each sequence as proposed by Chou [24, 25]. We used the composition/transition/distribution (CTD) model [26, 27] to generated 147D feature vectors for various physicochemical amino acid properties. Amino acids are divided into three classes in the CTD model. For each sequence, we obtained 3D, 3D and 15D feature vectors for each physicochemical property as measurements of the composition, transition, and distribution of the classes, respectively. Finally, we generated 6D feature vectors from the secondary structure (SS) of each sequence. The SS features includes position index, spatially consecutive states, and segment information of the 3 structure states: alpha helices, beta sheets and gamma coils. Finally, we obtained a total of 643 features as listed in Table 1.

**Table 1:** List of features

Feature	Dimension
AAC	20
DC	400
PseAAC	30
APseAAC	40
CTD	147
SS	6

Unnecessary features may worsen the prediction performance of a machine learning model and it is crucial to remove those features before building the model. We evaluated features solely on the training data to prevent information leakage in handling unseen values in the testing dataset. We performed statistical analyses on the training data to identify the optimal or best feature sets to build our machine learning models. At first, we estimated Pearson correlation coefficient $\rho_{x,y}$ given by Eq. 1, to measure the correlation values among features.

1 $$\rho_{x,y} = \frac{{E\left[ {\left( {x - \mu_{x} } \right)\left( {y - \mu_{y} } \right)} \right]}}{{\sigma_{x } \sigma_{y} }}$$

Here, *x* and *y* are two features, $E$ indicates the expectation,$\sigma_{x}$ and $\sigma_{y}$ indicate the standard deviation, and $\mu_{x}$ and $\mu_{y}$ are mean values of $x$ and $y$, respectively. High absolute the value of $\rho_{x,y}$ indicates strong correlation with other features. If a feature is highly correlated with another feature, we can consider one of these two features and ignore the other one. We removed one of the two features if they have correlation value was ≥ 0.9, which resulted in the number of features decreasing from 643 to 590.

**Fig. 2:**
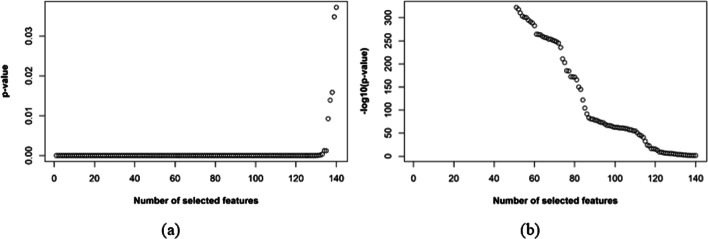
Trends of the *p-*values of the reduced feature set: **a** *p-*value vs. selected features and **b** − $log_{10}$(*p-*value) vs. selected features

Then we considered two additional statistical approaches to feature reduction. First, a standard *t-*test [28, 29] was applied to each of the 590 features to see if a statistically significant difference existed between the values of the feature in the positive and negative bacteriocin sequences of our dataset. We estimated the *p-*values for all 590 features to check if it was possible to discard the null hypothesis of no statistically significant difference. A low *p-*value for a feature indicates high importance of the feature for predicting bacteriocin sequences, and in that situation, we can discard the null hypothesis. We considered a threshold *p-*value of 0.05 and eliminated all features having *p* > 0.05. After filtering the features based on the *t-*test results, our feature vector was reduced from 590D to 140D, and we called the resulting data the *t-test-reduced feature set*. The *p-*values of the selected features are shown in Fig. 2 on linear and logarithmic scales. We noticed that the composition and distribution features of the CTD model were the top selected features in the *t*-test-reduced feature set.

We also built the random forest (RF) model with the 590 features (obtained from the Pearson correlation coefficient analysis) to estimate the mean decrease Gini (MDG). In the RF model, MDG corresponds to the feature importance that indicates each feature’s contribution to the homogeneity of the nodes and leaves [30, 31]. Equation 2, where $P_{i}$ is the probability of being in class *i* (positive or negative), was used to calculate the Gini index. A node is purer when its Gini index is closer to 0.

2 $$G = 1 - \mathop \sum \limits_{i = 1}^{2} P_{i}^{2}$$

Gini index of 0 and 1 corresponds to complete homogeneity and heterogeneity of the data, respectively. MDG is computed from the mean of all the drop of Gini indices across the whole of the trees built in the RF model. Greater MDG value indicates a more important feature, and with consideration of MDG values for the features, we reduced the dimension of the feature set to 44D and named it the *MDG-reduced feature set*. Features of the CTD model, PseAAC, and SS were identified as top selected features in the MDG-reduced feature set.

We further filtered features from the *t*-test-reduced and MDG-reduced feature sets using the recursive feature elimination (RFE) technique where a machine learning model is fitted, and features were ranked based on the evaluation of the training performance of the model. We considered two machine learning models RF and SVM in the RFE analyses. SVM maps data into a high dimensional space and identifies the hyperplane to separate the data of positive and negative classes. It considers a kernel function for the transformation of the data and a set of weights is used to define the hyperplane. There is a set of data points nearest to the hyperplane (called support vectors) that plays the crucial role in computing the decision boundary. RF is an ensemble model consisting of several decision trees where each tree is trained using a subset of the data. All decision trees independently make prediction on the data, and the final prediction is made by the RF by taking the majority votes of the decision trees. We applied 5 times repeated 10 fold cross-validation to assess the capability of the SVM and RF in the training phase in the RFE analyses. We obtained 42 (RF with MDG-reduced feature sets), 57 (RF with *t*-test-reduced feature sets), 44 (SVM with MDG-reduced feature sets) and 131 (SVM with *t*-test-reduced feature sets) features.

We trained SVM and RF models with different feature subsets obtained after RFE analyses. To find the best optimal feature set, we measured test performance of our tuned models, SVM and RF, for the reduced feature sets. We evaluated the prediction performance using Eqs. 3, 4, 5, 6 and 7, where TP, TN, FP, and FN correspond to true positives (correctly classified as positives values), true negatives (correctly classified as negative values), false positives (incorrectly classified as positive values), and false negatives (incorrectly classified as negative values), respectively. $Test_{Acc}$, $Test_{MCC}$, $Test_{recall}$, $Test_{precision}$, and $Test_{F1}$ indicate the accuracy, Matthews correlation coefficient (MCC) [32, 33], recall, precision, and F1 score, respectively, on the testing dataset. The MCC is considered to measure the effectiveness of our classifiers, with a value range of − 1 to + 1. The larger the MCC value, the better prediction is. The recall is used to measure how well a machine learning model can correctly predict positive examples with respect to all positive examples inputted to the model. The precision is used to measure the proportion of correct positive examples in the list of all predicted positive examples returned by the model. We calculated F1 score by taking the weighted average of precision and recall where the score of 1 and 0 indicate strong and poor prediction performance, respectively. We also estimated the confidence interval for the prediction results that provides upper and lower bound with a certain degree of confidence (in our case, 95%), where the true value of the outcome of the model exists. The higher the confidence interval, the greater the uncertainty of the predictions. All scripts used for the feature extraction, feature evaluation and performance comparison of SVM and RF models are available at https://github.com/suraiya14/ML_bacteriocins.

3 $$Test_{Acc} = \frac{TP + TN}{{TP + TN + FP + FN}}$$

4 $$Test_{MCC} = \frac{TP \times TN - FP \times FN}{{\sqrt {\left( {TP + FP} \right)\left( {TP + FN} \right)\left( {TN + FP} \right)\left( {TN + FN} \right)} }}$$

5 $$Test_{recall} = \frac{TP}{{TP + FN}}$$

6 $$Test_{precision} = \frac{TP}{{TP + FP}}$$

7 $$Test_{F1} = 2 \times \frac{{\left( {Test_{precision} \times Test_{recall} } \right)}}{{\left( {Test_{precision} + Test_{recall} } \right)}}$$

**Fig. 3:**
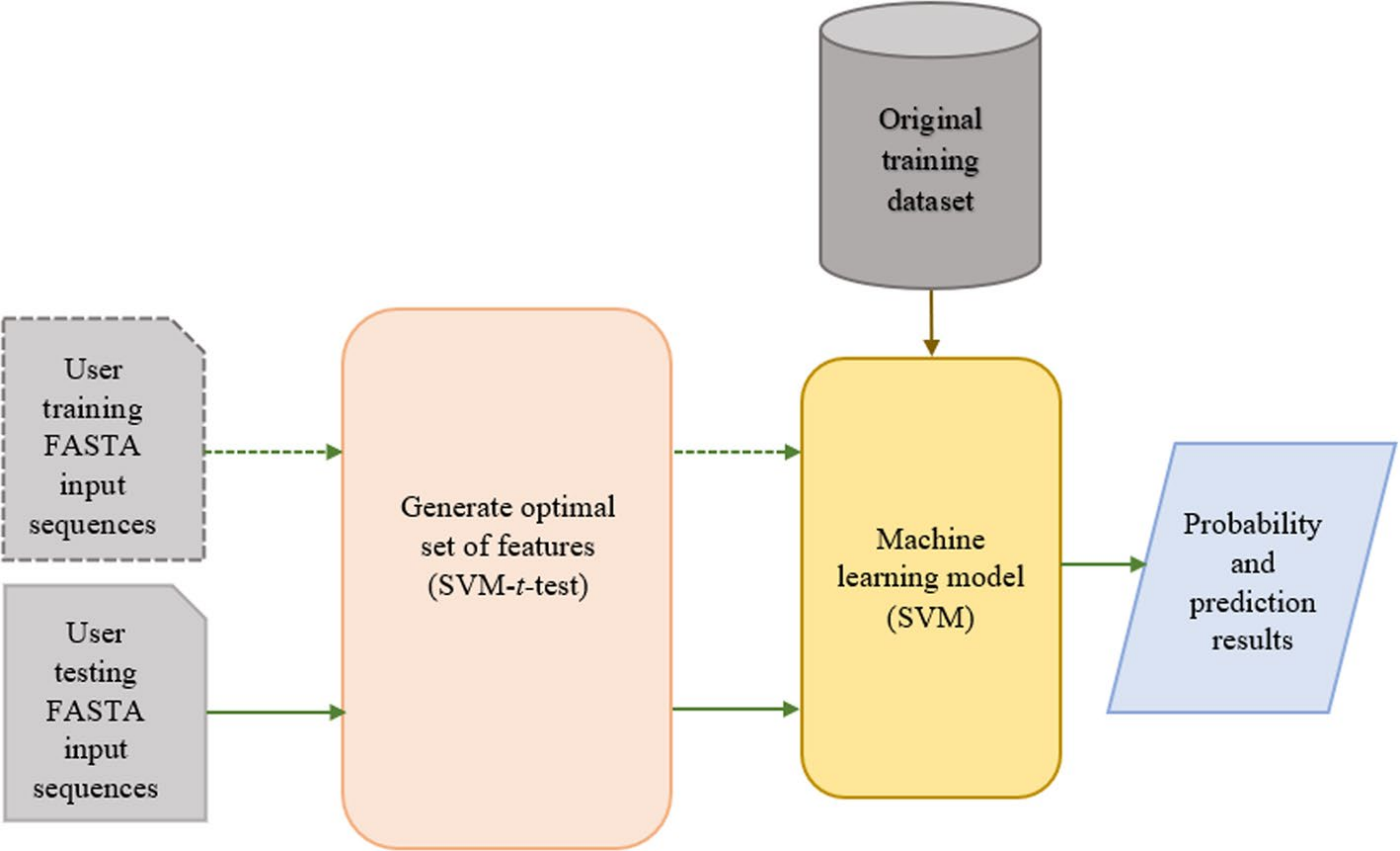
Architecture of the BaPreS software tool

**Fig. 4:**
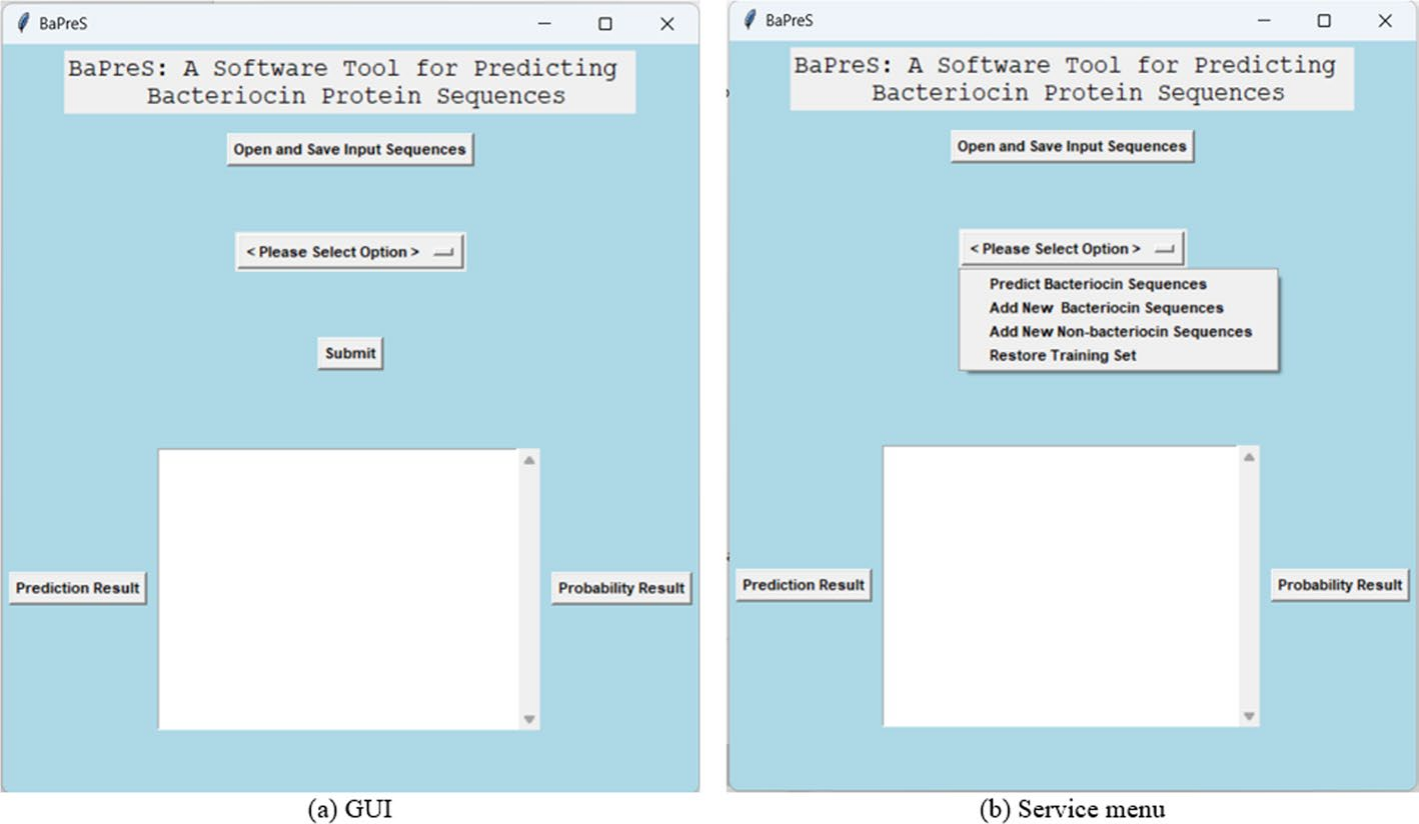
Graphical user interface (GUI) and various service menus of the BaPreS software tool

Finally, we implemented the BaPreS software tool using the machine learning model that showed the best prediction performance. Figures 3 and 4 show the architecture and GUI of the tool, respectively. All the required features in the BaPreS tool were generated using R and the GUI was designed using Python3. In this tool, users can upload and save an input file that should contain all protein sequences in FASTA format. If a user chooses the option of predicting bacteriocin, the BaPreS software tool will consider all protein sequences in the input file as testing sequences and generate all required optimal features with their feature values for the testing protein sequences automatically, classify them as bacteriocin or non-bacteriocin sequences and save the classification results with probability scores in two output files. Users can add new bacteriocin or non-bacteriocin protein sequences to the training dataset and return to the original training dataset supplied with this tool, if desired. The tool has a textbox in the GUI where users can see probability and classification results. The software package and the manual to use this software can be found at https://github.com/suraiya14/BaPreS.

## Results and discussion

**Fig. 5:**
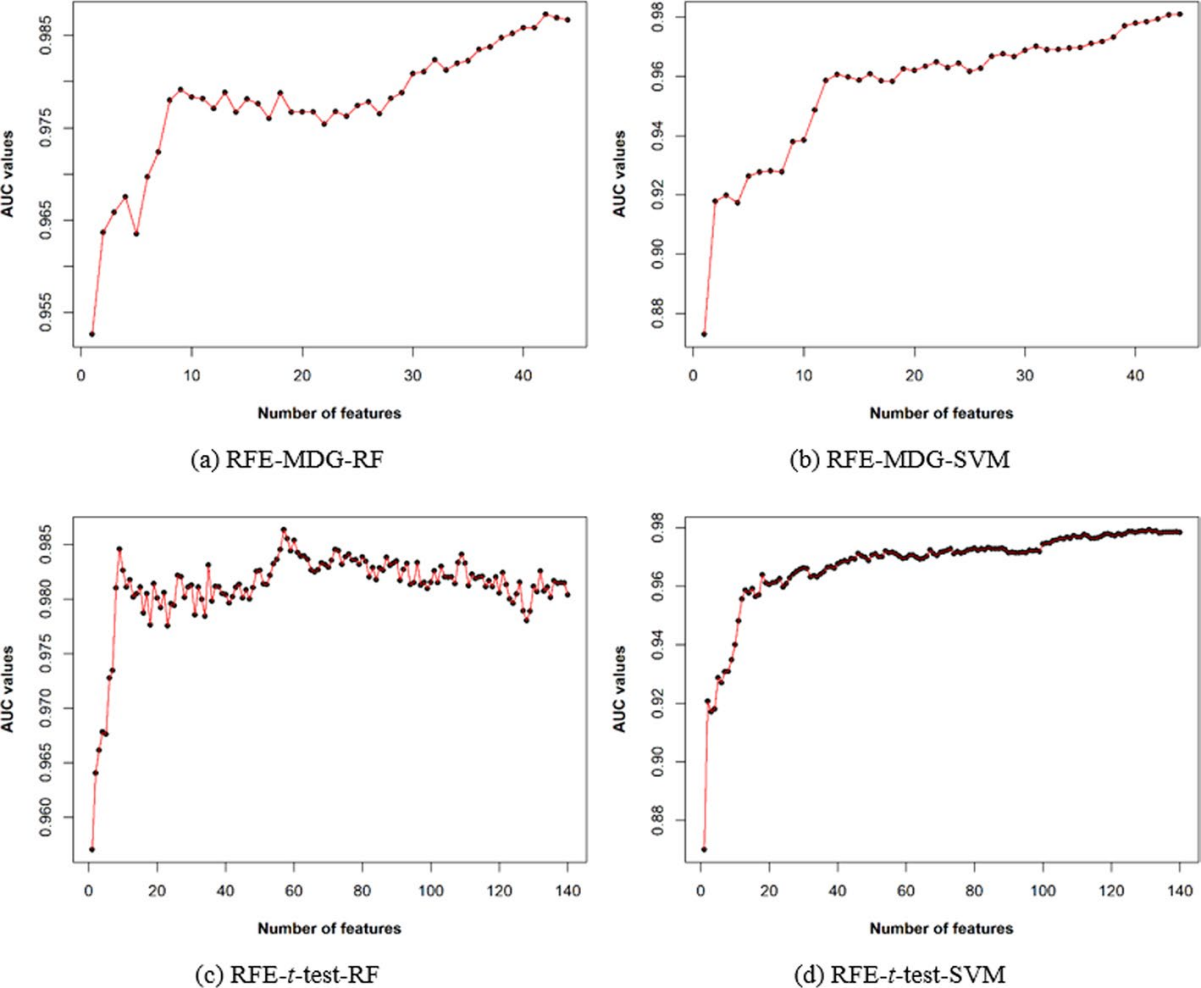
Performance of the RF and SVM machine learning models for the training data in the RFE approach

We mentioned earlier that SVM and RF machine learning models were used in the RFE approach to measure the training performance in terms of area under the receiver operating characteristic curve (AUC) by recursively considering subsets of the *t*-test-reduced and MDG-reduced feature sets independently. Figure 5(a) and (b) show the AUC values for the subset of the features in the RFE approach where RFE-MDG-RF and RFE-MDG-SVM depict the RFE analyses with the MDG-reduced feature sets for RF and SVM machine learning models, respectively. Similarly, Fig. 5(c) and (d) are RFE analyses with the *t*-test-reduced feature sets for RF and SVM machine learning models, respectively. We noticed gradual decreasing of AUC values with the elimination of the features from the machine learning models. Table 2 lists the maximum AUC values obtained from the machine learning models in the RFE analyses. We obtained the highest AUC value in the RF model for the MDG-reduced feature set. The top-5 features obtained from the RFE analyses are listed in Table 3. Features of the CTD model and PseAAC features are among the top ranked features for all models. More specifically, distribution (first residue) for secondary structure (group 1), distribution (first residue) for hydrophobicity (group 3) and distribution (first residue) for normalized van der Waals Volume (group 3) of the CTD model were found common in the top-5 features of all RFE analyses. It is known that most of the bacteriocins are cationic molecules having hydrophobic or amphiphilic characteristics and adopt diverse secondary structures, including alpha-helices, beta-sheets, and coils [19]. Therefore, the top-ranked features identified in the RFE analyses should play a critical role in predicting novel bacteriocins.

**Table 2:** Highest AUC values obtained from RF and SVM for different feature sets

Feature set	Machine learning model	AUC
MDG-reduced	RF	0.9873
	SVM	0.9809
*t*-test-reduced	RF	0.9864
	SVM	0.9794

**Table 3:** Top ranked features found from RF and SVM models in the RFE analyses

Feature rank	Feature for RFE-MDG-RF	Feature for RFE-MDG-SVM	Feature for RFE-*t*-test-RF	Feature for RFE-*t*-test-SVM
1	Distribution (first residue) for hydrophobicity (group 3)	PseAAC for the amino acid Leucine (L)	Distribution (first residue) for charge (group 2)	PseAAC for the amino acid Leucine (L)
2	Distribution (first residue) for secondary structure (group 1)	PseAAC for the amino acid Arginine (R)	Distribution (first residue) for hydrophobicity (group 3)	PseAAC for the amino acid Arginine (R)
3	Distribution (first residue) for charge (group 2)	Distribution (first residue) for hydrophobicity (group 3)	Distribution (first residue) for solvent accessibility (group 3)	Distribution (first residue) for hydrophobicity (group 3)
4	Distribution (first residue) for solvent accessibility (group 3)	Distribution (first residue) for secondary structure (group 1)	Distribution (first residue) for secondary structure (group 1)	Distribution (first residue) for secondary structure (group 1)
5	Distribution (first residue) for normalized van der Waals Volume (group 3)	Distribution (first residue) for normalized van der Waals Volume (group 3)	Distribution (first residue) for normalized van der Waals Volume (group 3)	Distribution (first residue) for normalized van der Waals Volume (group 3)

For our reduced feature sets, we trained SVM and RF models with different feature subsets obtained after RFE analyses. We tuned the SVM model with radial basis function (RBF) and set of cost values *C* = {4, 8, 16, 32, 64, 128} to find the best parameters. Similarly, we tuned the RF model with setting *ntree* = {400, 500} and *mtree* = {5, 6}. The RBF-kernel SVM with cost values of 4, 4, 4 and 8, and RF with *ntree* values of 500, 400, 500 and 400 and *mtree* values of 6, 5, 6 and 6 were found as best parameters for RFE-MDG-RF, RFE-MDG-SVM, RFE-*t*-test-RF and RFE-*t*-test-SVM feature sets, respectively. The prediction results of the models with corresponding best parameters for the testing dataset are shown as confusion matrices in Tables S1–S8 (Additional file 4) where ‘1’ and ‘− 1’ indicate positive (bacteriocin) and negative (non-bacteriocin) sequences, respectively. The diagonal entries in the confusion matrices show the correctly classified test sequences. The testing MCC, accuracy values, and confidence intervals of the RF and SVM models for different feature subset after RFE analyses are listed in Table 4. We found that the SVM machine learning model provides the best prediction values (based on MCC and accuracy values) for the RFE-*t*-test-SVM feature set, and prediction probability values and the predicted bacteriocin sequences obtained from this model for the testing dataset are presented in Table S9 (Additional file 5) and Additional file 6, respectively. We found that the best model identified 55 protein sequences as bacteriocins, of which the number of true positives is 53. We performed paired *t*-test on the probability values of positive and negative testing data for the best model (i.e., SVM with RFE-*t*-test-SVM feature set) and the second-best model (RF with RFE-MDG-SVM feature set). The prediction strength (based on the probability of 56 positive sequences) of SVM is higher than the RF model by 0.042 (*p* -value of 0.016). Thus, we obtained a more confident prediction in SVM model compared to the RF model and it is statistically significant if we consider *p*-value threshold of 0.05. For negative samples in the testing set, the mean of the probabilistic values of SVM is higher than the RF by 0.051 which is statistically significant as well (*p* -value of 0.007 < 0.05).

**Table 4:** MCC and accuracy values obtained from RF and SVM for testing data for different RFE feature subsets

Feature set after RFE	Machine learning models	$Test_{MCC}$	$Test_{Acc}$	Confidence interval
RFE-MDG-RF	RF	0.8763	0.9464	(0.887, 0.9801)
RFE-MDG-SVM	RF	0.8934	0.9464	(0.887, 0.9801)
RFE-MDG-RF	SVM	0.8219	0.9107	(0.8419, 0.9564)
RFE-MDG-SVM	SVM	0.8219	0.9107	(0.8419, 0.9564)
RFE-*t*-test-RF	RF	0.8763	0.9375	(0.8755, 0.9745)
RFE-*t*-test-SVM	RF	0.8593	0.9286	(0.8641, 0.9687)
RFE-*t*-test-RF	SVM	0.7862	0.8929	(0.8203, 0.9434)
RFE-*t*-test-SVM	SVM	0.9109	0.9554	(0.8989, 0.9853)

**Fig. 6:**
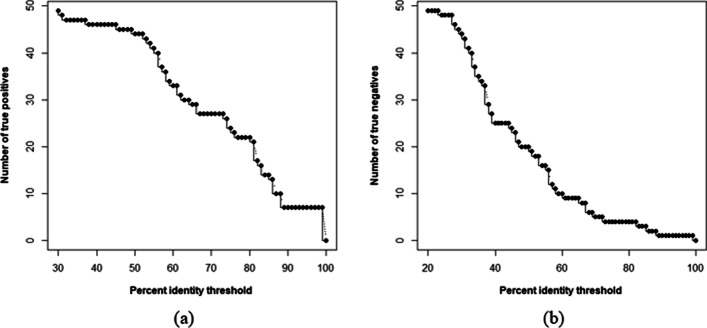
Identification of test sequences using BLASTP as a function of percent identity threshold **a** using bacteriocin sequences from the training data and **b** using non-bacteriocin sequences from the training data

We implemented BaPreS software tool using the best classifier i.e., the SVM model with RFE-*t*-test-SVM feature set. Our BaPreS's prediction performance was compared to the sequence matching tool BLASTP (https://blast.ncbi.nlm.nih.gov/Blast.cgi?PAGE=Proteins) [8, 9]. To identify bacteriocins sequences, BLASTP takes positive sequences of the training set as subject sequences and positive sequences of the testing set as query sequences and estimates the sequence similarity (percent identity) for each query sequence by aligning them with the subject sequences. Similarly, to detect non-bacteriocin sequences from BLASTP, we considered all negative sequences of the training and testing sets as subject and query sequences, respectively. Figure 6 shows the number of true positives and negatives with respective percent identity threshold for BLASTP tool. According to Table S8 (Additional file 4), our best classifier SVM model with RFE-*t*-test-SVM feature set has 53 true positives and 54 true negatives. BLASTP can identify a similar number of true positives and true negatives as our BaPreS if we set the percent identify threshold of BLASTP lower than 30 and 20 for finding the true positives and true negatives, respectively. However, setting such a low percent identify threshold in BLASTP is very unrealistic and will increase false positive and false negative results.

We also compared the performance of our BaPreS software tool with a recent deep learning-based method RMSCNN (https://github.com/cuizhensdws/RWMSCNN) [13] developed for the bacteriocin prediction. RMSCNN takes positive and negative training protein sequences in FASTA format as inputs, encodes all amino acids of each protein sequence to some numbers defined in a protein dictionary, then constructs a matrix of the encoded sequences. This matrix is used to train a convolutional neural network where a random model is used to modify the scale of the convolutional kernel. To compare the prediction accuracy, recall, precision, F1 score, and runtime with our BaPreS software tool, we ran RMSCNN with the same training and testing datasets that we used in our machine learning models. The runtime of RMSCNN or BaPreS is defined as the total time required in training and testing phases. Both RMSCNN and BaPreS were executed in a machine with macOS operating system, 2.3 GHz 8-Core Intel Core i9 processor, and 32 GB 2667 MHz DDR4 memory configuration. Table 5 shows the prediction accuracy, recall, precision, F1 score, and runtime of both methods/tools, and our BaPreS outperforms RMSCNN. As the input to the RMSCNN is the encoded protein sequences, it may suffer a similar problem like BLASTP in identifying highly dissimilar bacteriocin sequences. This could be a reason why our method showed better performance than RMSCNN.

**Table 5:** Accuracy and runtime (in seconds) of RMSCNN and BaPreS

Method/tool	$Test_{Acc}$	$Test_{recall}$	$Test_{precision}$	$Test_{F1}$	Runtime (sec.)
RMSCNN	0.9375	0.9107	0.9623	0.9358	2007.86
BaPreS	0.9554	0.9464	0.9636	0.9550	217.84

We can infer from Table 5 that BaPreS was able to utilize the most important features to detect highly diverse bacteriocin sequences with higher accuracy and lower runtime. Currently, our software tool is suitable to identify single bacteriocin protein sequence and we plan to update it to discover protein clusters of tailocins i.e., phage tail-like bacteriocins [34, 35]. Also, in the future, we will examine the feasibility of using other features such as position specific scoring matrix [36] in our tool and include a more robust feature selection algorithm such as partial least squares-based method to enhance the prediction accuracy of the tool. We plan to integrate feature stacking or ensemble techniques in the BaPreS tool to improve the generalization of our model. Whenever more nonduplicate bacteriocin sequences are available, we will retain our tool.

## Conclusions

Discovery of new bacteriocins is crucial to develop new antibiotic drugs to combat antibiotic resistance. In this paper, we presented a machine learning-based software tool for identifying novel bacteriocins. We extracted the applicant features from the primary and secondary attributes of protein sequences and then we analyzed all features based on Pearson correlation coefficient, *t- *test, and MDG values. We obtained two reduced feature sets of 140 and 44 features, and we further filtered out features using RFE technique. The final selected feature sets were considered as optimal sets of features and used to build the SVM and RF machine learning models. We found that SVM shows better prediction performance with the RFE-*t*-test-SVM-reduced feature set.

We implemented a software package BaPreS based on our best model to identify bacteriocin sequences by integrating all necessary tools and programs required for generating the optimal set of features automatically. Using our software tool, users will be able to predict unseen testing data for bacteriocin detection and can include new known bacteriocin and non-bacteriocin sequences to train data that eventually improve the predictive power of the machine learning model. The performance of BaPreS is compared to that of the sequence matching-based tool BLASTP. For BLASTP to obtain true positive as well as true negative results comparable to BaPreS requires a percent identity threshold so low that it is impractical. Also, our software tool showed better prediction accuracy with lower runtime compared to a deep learning-based method RMSCNN. Without having any programming knowledge, researchers can easily use our optimal feature-based software tool to discover novel bacteriocin sequences. Since our software tool is open source, they can modify our tool to fit it in similar or completely new biological applications.

### Supplementary Information

**Additional file 1** Training dataset composed of known bacteriocin and non-bacteriocin protein sequences.

**Additional file 2** Principal component analysis of the training dataset.

**Additional file 3** Testing dataset composed of known bacteriocin and non-bacteriocin protein sequences.

**Additional file 4** Confusion matrices of the machine learning models.

**Additional file 5** Prediction probability values for the testing dataset.

**Additional file 6** List of the predicted bacteriocin sequences.

## Data Availability

BaPreS has been implemented in R and Python3 programming languages and is available at https://github.com/suraiya14/BaPreS. In addition to the code, datasets and user manual of the software tool are also accessible. Project name: BaPreS, Project homepage: https://github.com/suraiya14/BaPreS, Operating systems: Windows, Linux, and MacOS, Programming languages: R and Python3, Requirements: R and Jupyter Notebook.

## Abbreviations

AAC: Amino acid composition
APseAAC: Amphiphilic pseudo amino acid composition
AUC: Area under the receiver operating characteristic curve
BaPreS: Bacteriocin prediction software
BOA: Bacteriocin operon associator
CTD: Composition/transition/distribution
DC: Dipeptide composition
GUI: Graphical user interface
MCC: Matthews correlation coefficient
MDG: Mean decrease Gini
PseAAC: Pseudo amino acid composition
RF: Random forest
RFE: Recursive feature elimination
SS: Secondary structure
SVM: Support vector machine
