# Supplementary material for: BaPreS: a software tool for predicting bacteriocins using an optimal set of features
Source: BMC Bioinformatics. 2023 Aug 17;24:313. doi: 10.1186/s12859-023-05330-z (PMC10433575; doi:10.1186/s12859-023-05330-z)
Supplement: Supplementary file 1 — Additional file 1 Training dataset composed of known bacteriocin and non-bacteriocin protein sequences. [file 12859_2023_5330_MOESM1_ESM.pdf]

Training dataset

Bacteriocin
--
>BAC010
CRQSCSFGPFTFVCDGNTK
>BAC011
CANSCSYGPLTWSCDGNTK
>BAC014
CTFTLPGGGGVCTLTSECIC
>BAC015
GGAGHVPEYFVGIGTPISFYG
>BAC017
IASKFICTPGCAKTGSFNSYCC
>BAC023
GNGVLKTISHECNMNTWQFLFTCC
>BAC024
GNPKVAHCASQIGRSTAWGAVSGA
>BAC025
NRWWQGVVPTVSYECRMNSWQHVFTCC
>BAC030
GKNGVFKTISHECHLNTWAFLATCCS
>BAC033
KGGSGVIHTISHEVIYNSWNFVFTCCS
>BAC034
KGGSGVIHTISHECNMNSWQFVFTCCS
>BAC042
KGKGFWSWASKATSWLTGPQQPGSPLLKKHR
>BAC043
SASVLKTSIKVSKKYCKGVTLTCGCNITGGK
>BAC045
WKSESLCTPGCVTGALQTCFLQTLTCNCKISK
>BAC047
ITSISLCTPGCKTGALMGCNMKTATCHCSIHVSK
>BAC048
TAGPAIRASVKQCQKTLKATRLFTVSCKGKNGCK
>BAC055
SDCNINSNTAADVILCFNQVGSCALCSPTLVGGPVP
>BAC056
KYYGNGLSCSKKGCTVNWGQAFSCGVNRVATAGHGK
>BAC057
STPVLASVAVSMELLPTASVLYSDVAGCFKYSAKHHC
>BAC058
KYYGNGVHCTKSGCSVNWGEAFSAGVHRLANGGNGFW
>BAC060
TSYGNGVHCNKSKCWIDVSELETYKAGTVSNPKDILW
>BAC062
GLGKAQCAALWLQCASGGTIGCGGGAVACQNYRQFCR
>BAC065
ARSYGNGVYCNNKKCWVNRGEATQSIIGGMISGWASGLAGM
>BAC069
AISYGNGVYCNKEKCWVNKAENKQAITGIVIGGWASSLAGMGH
>BAC070
TKYYGNGVYCNSKKCWVDWGTAQGCIDVVIGQLGGGIPGKGKC
>BAC071
KNYGNGVHCTKKGCSVDWGYAWTNIANNSVMNGLTGGNAGWHN

>BAC072
VGIGGGGGGGGGGSCGGQGGGCGGCSNGCSGGNGGSGGSGSHI
>BAC074
QINWGSVVGHCIGGAIIGGAFSGGAAAGVGCLVGSGKAIINGL
>BAC080
MGAIAKLVAKFGWPIVKKYYKQIMQFIGEGWAINKIIDWIKKHI
>BAC081
KYYGNGVSCNKNGCTVDWSKAIGIIGNNAAANLTTGGAAGWNKG
>BAC085
ANCSCSTASDYCPILTFCTTGTACSYTPTGCGTGWVYCACNGNFY
>BAC087
SLQYVMSAGPYTWYKDTRTGKTICKQTIDTASYTFGVMAEGWGKTFH
>BAC089
MDKFEKISTSNLEKISGGDLTTKLWSSWGYYLGKKARWNLKHPYVQF
>BAC090
ILFSYLLFYVLKENSKREDKYQNIIEELTELLPKIKEDVEDIKEKLNK
>BAC091
VNYGNGVSCSKTKCSVNWGQAFQERYTAGINSFVSGVASGAGSIGRRP
>BAC095
NRWTNAYSAALGCAVPGVKYGKKLGGVWGAVIGGVGGAAVCGLAGYVRKG
>BAC097
MQKPEIISADLGLCAVNEFVALAAIPGGAATFAVCQMPNLDEIVSNAAYV
>BAC098
MKLPVQQVYSVYGGKDLPKGHSHSTMPFLSKLQFLTKIYLLDIHTQPFFI
>BAC100
DQMSDGVNYGKGSSLSKGGAKCGLGIVGGLATIPSGPLGWLAGAAGVINSCMK
>BAC102
KLTFIQSTAAGDLYYNTNTHKYVYQQTQNAFGAAANTIVNGWMGGAAGGFGLHH
>BAC103
MNNLNKFSTLGKSSLSQIEGGSVPTSVYTLGIKILWSAYKHRKTIEKSFNKGFYH
>BAC118
ATYYGNGVYCNKQKCWVDWSRARSEIIDRGVKAYVNGFTKVLGGIGGR
>BAC119
GETDPNTQLLNDLGNNMAWGAALGAPGGLGSAALGAAGGALQTVGQGLIDHGPVNVPIPV
LIGPSWNGSGSGYNSATSSSGSGS
>BAC122
ASIIKTTIKVSKAVCKTLTCICTGSCSNCK
>BAC123
VPGGCTYTRSNRDVIGTCKTGSGQFRIRLDCNNAPDKTSVWAKPKVMVSVHCLVGQPRSI
SFETK
>BAC124
KYYGNGVHCGKKTCYVDWGQATASIGKIIVNGWTQHGPWAHR
>BAC125
DIAPPGPNGDPKSVQIDDKYTGAEMYGEGDFRVGLGTDLTMYPPVYRESLGNGSGGWEFD
FTVCGSTACRFVDSNGDVKEDDKAKEMWWQEINFNDINQDLYSRNDSDWVGSTPADTQPE
FDYTDFALARDGVTLALTALNPAMGSLALGATYFLSDMVNWIASQHEDDSSLKRKWDYDG
LSGPLYADSSTYLLARDEMTSNSYESFTIDNIAVAFPEFPVRTKYYVTFTAPDDPSTQSI
STLEEEGIYRVPATE
>BAC126
MARPIADLIHFNSTTVTASGDVYYGPGGGTGIGPIARPIEHGLDSSTENGWQEFESYADV
GVDPRRYVPLQVKEKRREIELQFRDAEKKLEASVQAELDKADAALGPAKNLAPLDVINRS
LTIVGNALQQKNQKLLLNQKKITSLGAKNFLTRTAEEIGEQAVREGNINGPEAYMRFLDR
EMEGLTAAYNVKLFTEAISSLQIRMNTLTAAKASIEAAAANKAREQAAAEAKRKAEEQAR
QQAAIRAANTYAMPANGSVVATAAGRGLIQVAQGAASLAQAISDAIAVLGRVLASAPSVM
AVGFASLTYSSRTAEQWQDQTPDSVRYALGMDAAKLGLPPSVNLNAVAKASGTVDLPMRL
TNEARGNTTTLSVVSTDGVSVPKAVPVRMAAYNATTGLYEVTVPSTTAEAPPLILTWTPA

SPPGNQNPSSTTPVVPKPVPVYEGATLTPVKATPETYPGVITLPEDLIIGFPADSGIKPI
YVMFRDPRDVPGAATGKGQPVSGNWLGAASQGEGAPIPSQIADKLRGKTFKNWRDFREQF
WIAVANDPELSKQFNPGSLAVMRDGGAPYVRESEQAGGRIKIEIHHKVRVADGGGVYNMG
NLVAVTPKRHIEIHKGGK
>BAC128
GWWNSWGKCVAGTIGGAGTGGLGGAAAGSAVPVIGTGIGGAIGGVSGGLTGAATFC
>BAC131
MDKVTDNSPDVESTESTEGSFPTVGVDTGDTITATLATGTENVGGGGGAFGGASESSAAI
HATAKWSTAQLKKHQAEQAARAAAAEAALAKAKSQRDALTQRLKDIVNDALRANAARSPS
VTDLAHANNMAMQAEAERLRLAKAEQKAREEAEAAEKALREAERQRDEIARQQAETAHLL
AMAEAAEAEKNRQDSLDEEHRAVEVAEKKLAEAKAELAKAESDVQSKQAIVSRVAGELEN
AQKSVDVKVTGFPGW
>BAC132
MGSNGADNAHNNAFGGGKNPGIGNTSGAGSNGSASSNRGNSNGWSWSNKPHKNDGFHSDG
SYHITFHGDNNSKPKPGGNSGNRGNNGDGASAKVGEITITPDNSKPGRYISSNPEYSLLA
KLIDAESIKGTEVYTFHTRKGQYVKVTVPDSNIDKMRVDYVNWKGPKYNNKLVKRFVSQF
LLFRKEEKEKNEKEALLKASELVSGMGDKLGEYLGVKYKNVAKEVANDIKNFHGRNIRSY
NEAMASLNKVLANPK
>BAC134
MKHLNETTNVRILSQFDMDTGYQAVVQKGNVGSKYVYGLQLRKGATTILRGYRGSKINNP
ILELSGQAGGHTQTWEFAGDRKDINGEERAGQWFIGVKPSKIEGSKIIWAKQIARVDLRN
QMGPHYSNTDFPRLSYLNRAGSNPFAGNKMTHAEAAVSPDYTKFLIATVENNCIGHFTIY
NLDTINEKLDEKGNSEDVNLETVKYEDSFIIDNLYGDDNNSIVNSIQGYDLDNDGNIYIS
SQKAPDFDGSYYAHH
>BAC135
METLTVHAPSPSTNLPSYGNGAFSLSAPHVPGAGPLLVQVVYSFFQSPNMCLQALTQLED
YIKKHGASNPLTLQIISTNIGYFCNADRNLVLHPGISVYDAYHFAKPAPSQYDYRSMNMK
QMSGNVTTPIVALAHYLWGNGAERSVNIANIGLKISPMKINQIKDIIKSGVVGTFPVSTK
FTHATGDYNVITGAYLGNITLKTEGTLTISANGSWTYNGVVRSYDDKYDFNASTHRGIIG
ESLTRLGAMFSGKEY
>BAC136
MNKTHKMATLVIAAILAAGMTAPTAYADSPGNTRITASEQSVLTQILGHKPTQTEYNRYV
ETYGSVPTEADINAYIEASESEGSSSQTAAHDDSTSPGTSTEIYTQAAPARFSMFFLSGT
WITRSGVVSLSLKPRKGGIGNEGDERTWKTVYDKFHNAGQWTRYKNNGVDASMKKQYMCH
FKYGMVKTPWNLEPHKKAADVSPVKCN
>BAC141
MSWLNFLKYIAKYGKKAVSAAWKYKGKVLEWLNVGPTLEWVWQKLKKIAGL
>BAC142
ATYYGNGLYCNKEKCWVDWNQAKGEIGKIIVNGWVNHGPWAPRR
>BAC143
MAKEFGIPAAVAGTVLNVVEAGGWVTTIVSILTAVGSGGLSLLAAAGRESIKAYLKKEIK
KKGKRAVIAW
>BAC148
LVAYGIAQGTAEKVVSLINAGLTVGSIISILGGVTVGLSGVFTAVKAAIAKQGIKKAIQL
>BAC150
KTVNYGNGLYCNQKKCWVNWSETATTIVNNSIMNGLTGGNAGWHSGGRA
>BAC153
TTKNYGNGVCNSVNWCQCGNVWASCNLATGCAAWLCKLA
>BAC154
DIGGSRQGCVA
>BAC157
MMNATENQIFVETVSDQELEMLIGGAGRGWIKTLTKDCPNVISSICAGTIITACKNCA
>BAC160
MQTIKELNTMELQEIIGGENDHRMPYELNRPNNLSKGGAKCAAGILGAGLGAVGGGPGGF
ISAGISAVLGCM
>BAC161

AYPGNGVHCGKYSCTVDKQTAIGNIGNNAA
>BAC162
LAGYTGIASGTAKKVVDAIDKGAAAFVIISIISTVISAGALGAVSASADFIILTVKNYIS
RNLKAQAVIW
>BAC164
MSLLALVAGTLGVSQSIATTVVSIVLTGSTLISIILGITAILSGGVDAILEIGWSAFVAT
VKKIVAERGKAAAIAW
>BAC166
WFYQGMNIAIYANIGGVANIIGYTEAAVATLLGAVVAVAPVVP
>BAC170
MAGFLKVVQILAKYGSKAVQWAWANKGKILDWINAGQAIDWVVEKIKQILGIK
>BAC172
MSDPVRITNPGAESLGYDSDGHEIMAVDIYVNPPRVDVFHGTPPAWSSFGNKTIWGGNEW
VDDSPTRSDIEKRDKEITAYKNTLSAQQKENENKRTEAGKRLSAAIAAREKDENTLKTLR
AGNADAADITRQEFRLLQAELREYGFRTEIAGYDALRLHTESRMLFADADSLRISPREAR
SLIEQAEKRQKDAQNADKKAADMLAEYERRKGILDTRLSELEKNGGAALAVLDAQQARLL
GQQTRNDRAISEARNKLSSVTESLNTARNALTRAEQQLTQQKNTPDGKTIVSPEKFPGRS
STNHSIVVSGDPRFAGTIKITTSAVIDNRANLNYLLSHSGLDYKRNILNDRNPVVTEDVE
GDKKIYNAEVAEWDKLRQRLLDARNKITSAESAVNSARNNLSARTNEQKHANDALNALLK
EKENIRNQLSGINQKIAEEKRKQDELKATKDAINFTTEFLKSVSEKYGAKAEQLAREMAG
QAKGKKIRNVEEALKTYEKYRADINKKINAKDRAAIAAALESVKLSDISSNLNRFSRGLG
YAGKFTSLADWITEFGKAVRTENWRPLFVKTETIIAGNAATALVALVFSILTGSALGIIG
YGLLMAVTGALIDESLVEKANKFWGI
>BAC173
MSDPVRITNPGAESLGYDSDGHEIMAVDIYVNPPRVDVFHGTPPAWSSFGNKTIWGGNEW
VDDSPTRSDIEKRDKEITAYKNTLSAQQKENENKRTEAGKRLSAAIAAREKDENTLKTLR
AGNADAADITRQEFRLLQAELREYGFRTEIAGYDALRLHTESRMLFADADSLRISPREAR
SLIEQAEKRQKDAQNADKKAADMLAEYERRKGILDTRLSELEKNGGAALAVLDAQQARLL
GQQTRNDRAISEARNKLSSVTESLKTARNALTRAEQQLTQQKNTPDGKTIVSPEKFPGRS
STNHSIVVSGDPRFAGTIKITTSAVIDNRANLNYLLTHSGLDYKRNILNDRNPVVTEDVE
GDKKIYNAEVAEWDKLRQRLLDARNKITSAESAINSARNNVSARTNEQKHANDALNALLK
EKENIRSQLADINQKIAEEKRKRDEINMVKDAIKLTSDFYRTIYDEFGKQASELAKELAS
VSQGKQIKSVDDALNAFDKFRNNLNKKYNIQDRMAISKALEAINQVHMAENFKLFSKAFG
FTGKVIERYDVAVELQKAVKTDNWRPFFVKLESLAAGRAASAVTAWAFSVMLGTPVGILG
FAIIMAAVSALVNDKFIEQVNKLIGI
>BAC174
NRWYCNSAAGGVGGAAVCGLAGYVGEAKENIAGEVRKGWGMAGGFTHNKACKSFPGSGWA
SG
>BAC176
AGDPLADPNSQIVRQIMSNAAWGPPLVPERFRGMAVGAAGGVTQTVLQGAAAHMPVNVPI
PKVPMGPSWNGSKG
>BAC178
KGLGKLIGIDWLLGQAKDAVKQYKKDYKRWH
>BAC181
AVPAVRKTNETLD
>BAC182
GNGVVLTLTHECNLATWTKKLKCC
>BAC185
KPAWCWYTLAMCGAGYDSGTCDYMYSHCFGVKHSSGGGGSYHC
>BAC186
NETNNFAETQKEITTNSEATLTNEDYTKLTSEVKTIYTNLIQYDQTKNKFYVDEDKTEQY
YNYDDESIKGVYLMKDSLNDELNNNNSSNYSEIINQKISEIDYVLQGNDINNLIPSNTRV
KRSADFSWIQRCLEEAWGYAISLVTLKGIINLFKAGKFEAAAAKLASATAGRIAGMAALF
AFVATCGATTVS
>BAC187
PNWTKIGKCAGSIAWAIGSGLFGGAKLIKIKKYIAELGGLQKAAKLLVGATTWEEKLHAG

GYALINLAAELTGVAGIQANCF
>BAC189
SNDSLWYGVGQFMGKQANCITNHPVKHMIIPGYCLSKILG
>BAC190
IAPIIVAGLGYLVKDAWDHSDQIISGFKKGWNGGRRK
>BAC191
LIDHLGAPRWAVDTILGAIAVGNLASWVLALVPGPGWAVKAGLATAAAIVKHQGKAAAAA
W
>BAC192
LVATGMAAGVAKTIVNAVSAGMDIATALSLFSGAFTAAGGIMALIKKYAQKKLWKQLIAA
>BAC193
PNDGDTMTVSGGGGWVSNDDRKGGNDRDNGKGGSAVDFSKNPEKQAIVNPYLAIAIPMPV
YPLYGKLGFTINTTAIETELANVRAAINTKLATLSAVIGRSLPVVGRVFGVTAAGMWPSS
TAPSSLDSIYNQAHQQALAQLAAQQGVLNKGYNVTAMPAGFVSSLPVSEIKSLPTAPASL
LAQSVINTELSQRQLALTQPTTNAPVANIPVVKAEKTAMPGVYSAKIIAGEPAFQIKVDN
TKPALAQNPPKVKDDIQVSSFLSSPVADTHHAFIDFGSDHEPVYVSLSKIVTAEEEKKQV
EEAKRREQEWLLRHPITAAERKLTEIRQVISFAQQLKESSVATISEKTKTVAVYQEQVNT
AAKNRDNFYNQNRGLLSAGITGGPGYPIYLALWQTMNNFHQAYFRANNALEQESHVLNLA
RSDLAKAEQLLAENNRLQVETERTLAEEKEIKRNRVNVSTFGTVQTQLSKLLSDFYAVTS
LSQSVPSGALASFSYNPQGMIGSGKIVGKDVDVLFSIPVKDIPGYKSPINLDDLAKKNGS
LDLPIRLAFSDENGERVLRAFKADSLRIPSSVRGVAGSYDKNTGIFSAEIDGVSSRLVLE
NPAFPPTGNVGNTGNTAPDYKALLNTGVDVKPVDKITVTVTPVADPVDIDDYIIWLPTAS
GSGVEPIYVVFNSNPYGGTEKGKYSKRYYNPDKAGGPILELDWKNVKIDHAGVDNVKLHT
GRFKASVENKVMIERLENILNGQITATDTDKRFYTHELRELNRYRNLGIKDGEVPSSIQE
ESAVWNDTHTATLEDYKINEKEQPLYTDAALQAAYEQELKDALGGKHG
>BAC196
TITLSTCAILSKPLGNNGYLCTVTKECMPSCN
>BAC197
TTPATTSSWTCITAGVTVSASLCPTTKCTSRC
>BAC198
ATYTRPLDTGNITTGFNGYPGHVGVDYAVPVGTPVRAVANGTVKFAGNGANHPWMLWMAG
NCVLIQHADGMHTGYAHLSKISVSTDSTVKQGQIIGYTGATGQVTGPHLHFEMLPANPNW
QNGFSGRIDPTGYIANAPVFNGTTPTEPTTPTTNLKIYKVDDLQKINGIWQVRNNILVPT
DFTWVDNGIAADDVIEVTSNGTRTSDQVLQKGGYFVINPNNVKSVGTPMKGSGGLSWAQV
NFTTGGNVWLNTTSKDNLLYGK
>BAC199
KPAWCWYTLAMCGAGYDSGTCDYMYSHCFGIKHHSSGSSSYHC
>BAC200
KNYGNGVYCNKHKCSVDWATFSANIANNSVAMAGLTGGNAGNK
>BAC202
KYYGNGVSCNSHGCSVNWGQAWTCGVNHLANGGHGVC
>BAC203
MLAKIKAMIKKFPNPYTLAAKLTTYEINWYKQQYGRYPWERPVA
>BAC204
ATYYGNGLYCNKQKHYTWVDWNKASREIGKIIVNGWVQH
>BAC205
FVYGNGVTSILVQAQFLVNGQRRFFYTPDK
>BAC206
VNYGNGVSCSKTKCSVNWGIITHQAFRVTSGVASG
>BAC208
TNYGNGVGVPDAIMAGIIKLIFIFNIRQGYNFGKKAT
>BAC209
MFLVNQLGISKSLANTILGAIAVGNLASWLLALVPGPGWATKAALATAETIVKHEGKAAA
IAW
>BAC210
MAAFMKLIQFLATKGQKYVSLAWKHKGTILKWINAGQSFEWIYKQIKKLWA

>BAC211
ACQCPDAISGWTHTDYQCHGLENKMYRHVYAICMNGTQVYCRTEWGSSC
>BAC212
AIKLVQSPNGNFAASFVLDGTKWIFKSKYYDSSKGYWVGIYEVWDRK
>BAC214
VTTSIPCTVMVSAAVCPTLVCSNKCGGRG
>BAC216
GNAACVIGCIGSCVISEGIGSLVGTAFTLG
>BAC218
ATPATPTVAQFVIQGSTICLVC
>BAC219
IGGALGNALNGLGTWANMMNGGGFVNQWQVYANKGKINQYRPY
>BAC222
VTSWSLCTPGCTSPGGGSNCSFCC
>BAC223
MKTILRFVAGYDIASHKKKTGGYPWERGKA
>BAC224
IVWLANKFGVHLTNHLTNSILNAVSNGSSLGSAFAVIAGVTLPGWAVAAVGALGATAA
>BAC225
VFHAYSARGNYYGNCPANWPSCRNNYKSAGGK
>BAC229
LTANLGISSYAAKKVIDIINTGSAVATIIALVTAVVGGGLITAGIVATAKSLIKKYGAKY
AAAW
>WP_061432710.1
MKNPTLLPKLTAPVERPAVTSSDLKQASSVDAAWLNGDNNWSTPFAGVNAAWLNGDNNWS
TPFAGVNAAWLNGDNNWSTPFAADGAE
>CAX48972.1
MASILELQDLEVERASSAADSNASVWECCSTGSWVPFTCC
>YP_003491235.1
MGPVVVFDCMTADFLNDDPNNAELSALEMEELESWGAWDGEATS
>WP_013079675.1
MTKKNATQAPRLVRVGDAHRLTQGAFVGQPEAVNPLGREIQG
>ACR33052.1
MSALAIEKSWKDVDLRDGATSHPAGLGFGELTFEDLREDRTIYAASSGWVCTLTIECGTV
ICAC
>sp|Q09T02.1|MICA_CLAMM
MNDILETETPVMVSPRWDMLLDAGEDTSPSVQTQIDAEFRRVVSPYMSSSGWLCTLTIEC
GTIICACR
>NP_391616.1
MKKAVIVENKGCATCSIGAACLVDGPIPDFEIAGATGLFGLWG
>sp|O87236.1|LANA1_LACLL
MNKNEIETQPVTWLEEVSDQNFDEDVFGACSTNTFSLSDYWGNNGAWCTLTHECMAWCK
>sp|O87237.1|LANA2_LACLL
MKEKNMKKNDTIELQLGKYLEDDMIELAEGDESHGGTTPATPAISILSAYISTNTCPTTK
CTRAC
>sp|O88038.1|LANSB_STRCO
MNLFDLQSMETPKEEAMGDVETGSRASLLLCGDSSLSITTCN
>AAK33966.1
MNNTIKDFDLDLKTNKKDTATPYVGSRYLCTPGSCWKLVCFTTTVK
>YP_444120.1
MRTLTLNELDSVSGGASGRDIAMAIGTLSGQFVAGGIGAAAGGVAGGAIYDYASTHKPNP
AMSPSGLGGTIKQKPEGIPSEAWNYAAGRLCNWSPNNLSDVCL
>AAL73241.1
MSNTQLLEVLGTETFDVQENLFTFDTTDTIVAESNDDPDTRFKSWSFCTPGCAKTGSFNS
YCC
>sp|Q2QBT0.1|LANNU_STRUB

MNNEDFNLDLIKISKENNSGASPRITSKSLCTPGCKTGILMTCPLKTATCGCHFG
>ANP43731.1
MLDVIKNRKKIEEKLELPEILLEEVEEHSAMGGINTWNTTATSTSIIISETFGNKGKVCT
YTVECVNNCRG
>CAA84399.1
MVTKYGRNLGLSKVELFAIWAVLVVALLLATANIYWIADQFGIHLATGTARKLLDAVASG
ASLGTAFAAILGVTLPAWALAAAGALGATAA
>CAA63706.1
MTNAFQALDEVTDAELDAILGGGSGVIPTISHECHMNSFQFVFTCCS
>sp|Q52053|Q52053_9ZZZZ
MLNKENQENYYSNKLELVGPSFEELSLEEMEAIQGSGDVQAETTPACFTIGLGVGALFSA
KFC
>BAD72777.1
MKEIQKAGLQEELSILMDDANNLEQLTAGIGTTVVNSTFSIVLGNKGYICTVTVECMRNC
SK
>ANP43734.1
MLKEEKLEKITGLIPESELEEHLSGESSGAGTPAITTAISAIIAATAQSPCPTSACSKSC
NK
>AAT87775.1
MERRMSFMKNSKDILTNVIEEVSEKELMEVAGGKKGSGWFATITDDCPNSVFVCC
>BAD05046.1
MSTKDFNLDLVSVSKTDSGASTRITSISLCTPGCKTGVLMGCNLKTATCNCSVHVSK
>AAN86036.1
MFLVAGALGVQTAAATTIVNVILNAGTLVTVLGIIASIASGGAGTLMTIGWATFKATVQK
LAKQSMARAIAY
>AAL15569.1
MSKFDDFDLDVVKVSKQDSKITPQWKSESVCTPGCVTGVLQTCFLQTITCNCHISK
>AAL15567.1
MTNMSKFDDFDLDVVKVSKQDSKITPQVLSKSLCTPGCITGPLQTCYLCFPTFAKC
>AAK32702.1
MNKDLNALTNPIDEKELEQILGGGDGVFRTISHECAMNTWMFIFTCCS
>ARW80050.1
MSMTMTLQQAVVDDEFRSVLLADPAAFGLSVESLPGAVERQDHEAIEAFTEAVVASEIYA
CASTCSFGPFTIACDGTTK
>BAB04173.1
MTNLLKEWKMPLERTHNNSNPAGDIFQELEDQDILAGVNGACAWYNISCRLGNKGAYCTL
TVECMPSCN
>NP_478384.1
MKNELGKFLEENELELGKFSESDMLEITDDEVYAAGTPLALLGGAATGVIGYISNQTCPT
TACTRAC
>NP_478383.1
MKSSFLEKDIEEQVTWFEEVSEQEFDDDIFGACSTNTFSLSDYWGNKGNWCTATHECMSW
CK
>NP_834755.1
MSEIKKALNTLEIEDFDAIEMVDVDAMPENEALEIMGASCTTCVCTCSCCTT
>AHJ59549.1
MSKGYKFTKEELVEAWKDPQVREKLKDLPKHPSGKALNELSEEELAEIQGASDVQPETTP
LCVGVIIGLTTSIKICK
>WP_015792833.1
MTEEMTLLDLQGMEQTETDSWGGSGHGGGGDSGLSVTGCNGHSGISLLCDL
>CAG43551.1
MEKVLDLDVQVKGNNNTNDSAGDERITSHLFCSFGCEKTGSFNSFCC
>AAC69560.1
MSKKQIMSNCISIALLIALIPNIYFIADKMGIQLAPAWYQDIVNWVSAGGTLTTGFAIIV
GVTVPAWIAEAAAAFGIASA
>WP_067999479.1

MSAQGKDPNEIRRRFEELPMEVFQLDGSGLPIESLTDGHGMTEVGASCTSCVCICSCCT
>EFI65094.1
MTNEEIIVAWKNPKVRGKNMPSHPSGVGFQELSINEMAQVTGGAVEQRATPATPATPWLI
KASYVVSGAGVSFVASYITVN
>EFI65095.1
MTNEEIIVAWKNPKVRGKNMPSHPSGVGFQELSINEMAQVTGGAVEQRATPTLATPLTPH
TPYATYVVSGGVVSAISGIFSNNKTCLG
>BAN83916.1
MTEKTQITDVQAFEDLVAKVQEMDGPAQASSTVAALAGLDAAELQNFLEEKSGISPDEEA
QGSVMAAAASIALHC
>WP_013079674.1
MTKTHRLIRLGDAQRLTQGTLTPGLPEDFLPGHYMPG
>SED43766.1
MTEQSEQTPTEYIPPMLVEVGEFTEDTLGNWHGTSPDWFFNYYW
>EGD17355.1
MDTSNNDARTTALDQDLIVLGVASLDTQGGPLAGEEMGGITTLGISQD
>EDY58505.1
MLISTTNGQGTPMTSTDELYEAPELIEIGDYAELTRCVWGGDCTDFLGCGTAWICV
>EFE76491.1
MQKSVGHNGRQPRRREGVMKQQKQQKKAYVKPSMFQQGDFSKKTAGYFVGSYKEYWSRRI
I
>WP_043998581.1
MDKKNILPHQGKPVLRTTNGKLPSHLAELSEEALGGAGMDASFFPCSYDGADASFFPVCS
YDGADASFFPCSYDDGDA
>CAP64339.1
MRITPMDKKNLLPNQGAPVIRGISGKLPSHLAELSEEALGGNGAEASATVSICAFDGAEA
SFTGCMCAFDGAEASITGCICAFDGAEASITGCICAFDGDEA
>sp|Q07642.1|LANSB_STRGR
MALLDLQAMDTPAEDSFGELRTGSQVSLLVCEYSSLSVVLCTP
>NP_604414.1
MISSHQKTLTDKELALISGGKTHYPTNAWKSLWKGFWESLRYTDGF
>NP_345049.1
MNTKMLSQLEVMDTEMLAKVEGGYSSTDCQNALITGVTTGIITGGTGAGLATLGVAGLAG
AFVGAHIGAIGGGLTCLGGMVGDKLGLSW
>AAB91455.1
MNTITICKFDVLDAELLSTVEGGYSGKDCLKDMGGYALAGAGSGALWGAPAGGVGALPGA
FVGAHVGAIAGGFACMGGMIGNKFN
>ZP_04066356.1
MRTMEEQIFNSMIQQGAFAALFVWMLFTTQKKNEQREEQYQKVIEKNQQVIEEQAKAFSS
LSKDLSDVKQKILGNGDEK
>ZP_04066940.1
MRTMEEQIFNSMIQQGAFAALFVWMLFTTQKKNEQREEQYQKVIEKNQDVITKQAEAFGD
LSKDVSEIKQKILGSGDVQ
>NP_345050.1
MDTKMMSQFSVMDTEMLACVEGGGCNWGDFAKAGVGGGAARGLQLGIKTGTWQGAATGAA
GGAILGGVAYAATCWW
>NP_345056.1
MDTKIMEQFHEMDITMLSSIEGGKNNWQTNVLEGGGAAFGGWGLGTAICAASGVGAPFMG
ACGYIGAKFGVDLWAGVTGATGGF
>NP_345057.1
MNTYCNINETMLSEVYGGNSGGAAVVAALGCAAGGVKYGRLLGPWGAAIGGIGGAVVCGY
LAYTATS
>NP_345058.1
MDTKMMSQFAVMDNEMLACVEGGDIDWGRKISCAAGVAYGAIDGCATTV
>YP_140101.1
MATQTIENFNTLDLETLASVEGGGCSWGGFAKQGVATGVGNGLRLGIKTRTWQGAVAGAA

GGAIVGGVGYGATCWW
>AAG29818.1
MNTKTFEQFDVMTDEALSTVEGGGKGYCKPVYYAANGYSCRYSNGEWGYVVTKGAFQATT
DVIANGWVSSLGGGYFGKP
>AAC95138.1
MHKVKKLNNQELQQIVGGYSSKDCLKDIGKGIGAGTVAGAAGGGLAAGLGAIPGAFVGAH
FGVIGGSAACIGGLLGN
>AAC95139.1
MKKELLNKNEMSRIIGGKINWGNVGGSCVGGAVIGGALGGLGGAGGGCITGAIGSIWDQW
>AAG28763.1
MKKIEKLTEKEMANIIGGKYYGNGVTCGKHSCSVDWGKATTCIINNGAMAWATGGHQGTH
KC
>NP_297555.1
MRELTLTEIDNVSGADLGSRLSAAIVGGVAAFFAGSIWGGTRGGDGGGILGVGSIGQGVG
MVYGGIAGAIGGAIAGFVLDKDVIYSYTNGFMSSIFNGTFAK
>CAA90906.1
MIKREKNRTISSLGYEEISNHKLQEIQGGKGILGKLGVVQAGVDFVSGVWAGIKQSAKDH
PNA
>AAZ29031.1
MKKQILKGLVIVVCLSGATFFSTPQQASAAAPKITQKQKNCVNGQLGGMLAGALGGPGGV
VLGGIGGAIAGGCFN
>AAZ29032.1
MKIKWYWESLIETLIFIIVLLVFFYRSSGFSLKNLVLGSLFYLIAIGLFNYKKINK
>ZP_03980216.1
MTNFGTKVDAATRSYDNGIYCNNSKCWVNWGEAKENIAGIVISGWASGLAGMGH
>AAF44686.1
MKHLKILSIKQTQLIYGGTTHSGKYYGNGVYCTKNKCTVDWAKATTCIAGMSIGGFLGGA
IPGKC
>NP_863263.1
MKNIKNASNIKVIEDNELKAITGGGPGKWLPWLQPAYDFVTGLAKGIGKEGNKNKWKNV
>AAD28234.1
MQNVKELSTKEMKQIIGGENDHRMPNELNRPNNLSKGGAKCGAAIAGGLFGIPKGPLAWA
AGLANVYSKCN
>AAQ95741.1
MKKLTSKEMAQVVGGKYYGNGVSCNKKGCSVDWGKAIGIIGNNSAANLATGGAAGWKS
>BAA07120.1
MISMISSHQKTLTDKELALISGGKTYYGTNGVHCTKKSLWGKVRLKNVIPGTLCRKQSLP
IKQDLKILLGWATGAFGKTFH
>BAA82353.1
MKNFNTLSFETLANIVGGRNNWAANIGGVGGATVAGWALGNAVCGPACGFVGAHYVPIAW
AGVTAATGGFGKIRK
>NP_964623.1
MKLNDKELSKIVGGNRWGDTVLSAASGAGTGIKACKSFGPWGMAICGVGGAAIGGYFGYT
HN
>NP_542216.1
MDNLNKFKKLSDNKLQATIGGGMSGYIQGIPDFLKGYLHGISAANKHKKGRLGY
>NP_542217.1
MESNKLEKFANISNKDLNKITGGGFWGGLGYIAGRVGAAYGHAQASANNHHSPING
>YP_288875.1
MNALKRTCATLLISAGLTAGAVGVAAAAVEYVGGGIWDHGLTSSIVYSDYYHGSVCHGST
AVGTKTVRASAPAGYWSLADAPRAIANNQAYWRTTC
>NP_862432.1
MKTKSLVLALSAVTLFSAGGIVAQAEGTWQHGYGVSSAYSNYHHGSKTHSATVVNNNTGR
QGKDTQRAGVWAKATVGRNLTEKASFYYNFW
>ACR43769.1
MKELSEKELRECVGGGTWDDIGQGIGRVAYWVGKAMGNMSDVNQASRINRKKKH

>NP_268769.1
MIKFAEEIQKEELFHIIGGYSATDCKNHLIGGITSGAIAGGVGAGMATLGVGGVAGAFAG
AHVGAIAGGLTCVGGMLFNGK
>ACR43770.1
MKNNNNFFKGMEIIEDQELVSITGGKKWGWLAWVDPAYEFIKGFGKGAIKEGNKDKWKNI
>AAT72009.2
MNKTKSEHIKQQALDLFTRLQFLLQKHDTIEPYQYVLDILETGISKTKHNQQTPERQARV
VYNKIASQALVDKLHFTAEENKVLAAINELAHSQKGWGEFN
>NP_784211.1
MKIQIKGMKQLSNKEMQKIVGGKSSAYSLQMGATAIKQVKKLFKKWGW
>NP_784217.1
MLQFEKLQYSRLPQKKLAKISGGFNRGGYNFGKSVRHVVDAIGSVAGIRGILKSIR
>NP_784216.1
MKKFLVLRDRELNAISGGVFHAYSARGVRNNYKSAVGPADWVISAVRGFIHG
>NP_784205.1
MTVNKMIKDLDVVDAFAPISNNKLNGVVGGGAWKNFWSSLRKGFYDGEAGRAIRR
>ZP_04015571.1
MKIKLTVLNEFEELTADAEKNISGGRRSRKNGIGYAIGYAFGAVERAVLGGSRDYNK
>NP_784207.1
MKSLDKIAGLGIEMAEKDLTTVEGGKNYSKTWWYKSLTLLGKVAEGTSSAWHGLG
>AAG02566.1
MTKTSRRKNAIANYLEPVDEKSINESFGAGDPEARSGIPCTIGAAVAASIAVCPTTKCSK
RCGKRKK
>AAX99121.1
MKKKFVSSCIASTILFGTLLGVTYKAEAATVHVAGGVWSHGIGKHYVWSHYSHNKRNHGS
TAVGKYSSFSGVARPGVQSKASAPKAWGGNKTFYSLH
>NP_345048.1
MNTKMMEQFSVMDNEELEIVSGGRGNLGSAIGGCIGAVLLAAATGPITGGAATLICVGSG
IMSSL
>NP_720757.1
MNTKMMEQFETMDAETLSHVTGGGLYDGANGYAYRDSQGHWAYKVTKTPAQALTDVVVNS
WASGAASFAAYA
>YP_279852.1
MILFFMIFCTSSRLQRDKFKNYEKKLFDMEIKKLETFHQMTIEKLAKVEGGKNNWQANVS
GVIAAGSAGAAIGFPVCGVACGYIGAKTAITLWAGVTGATGGF
>AAP44569.1
MEAIKKLDLQAMKGIVGGKYYGNGLSCNKSGCSVDWSKAISIIGNNAVANLTTGGAAGWK
S
>AAP44566.1
MKNVQSLSKEELVLVVGGYTAKQCLQAIGSWGIAGTGAGAAGGPAGAFVGAHVGVIAGSA
VCIGGFLGQ
>AAP44567.2
MKTANIKLLTNQEMIEIFGGKTNWGSVVGSCVAGGLVGALGGTPISIGAGCLVGAGQDWI
SQK
>AAY68489.1
METAVAYYKDGVPYDDKGQVIITLLNGNPDGSGSGSGGGGGTGGSKSESSAAIHATAKWS
TAQLKKTQAEQAARAKAAAEAQAKAKANRDALTQHLKDIVNEALRHNSTHPEVIDLAHAN
NAAMQAEAERLRLAKAEEKARKEAEAAEKAFQEAEQRRKEIEKEQAETERQLKLAEDEEK
RLAALSEEARAVEVAQKNLAAAQSELAKVDEEINTLNTRLSSSIHARDAETNTLSGKRNE
LDQASAKYKELDERVKLLSPRANDPLQSRPFFEATRLRARAGDEMEEKQKQVTASETRLN
QISSEINGIQEAISQANNKRSTAVSRIHDAEDNLKTAQTNLLNSQIKDAVDATVSFYQTL
SEKYGEKYSKMAQELADKSKGKKISNVNEALAAFEKYKDVLNKKFSKADRDAIFNALEAV
KYEDWAKHLDQFAKYLKITGHVSFGYDVVSDILKIKDTGDWKPLFLTLEKKAVDAGVSYV
VVLLFSVLAGTTLGIWGIAIVTGILCAFIDKNKLNTINEVLGI
>YP_025360.1
MSGGDGKGHNSGAHDSGGSINGTSGKGGPDSGGGYWDNHPHITITGGREVGQGGAGINWG

GGSGHGNGGGSVAIQEYNTSKYPNTGGFPPLGDASWLLNPPKWSVIEVKSENSAWRSYIT
HVQGHVYKLTFDGTGKLIDTAYVNYEPSDDTRWSPLKSFKYNKGTAEKQVRDAINNEKEA
VKDAVKFTADFYKEVFKVYGEKAEKLAKLLADQAKGKKVRNVEDALKSYEKYKTNINKKI
NAKDREAIAKALESMDVGKAAKNIAKFSKGLGWVGPAIDITDWFTELYKAVETDNWRSFY
VKTETIAVGLAATHVAALAFSAVLGGPVGILGYGLIMAGVGALVNETIVDEANKVIGL
>sp|Q47502.1|CEAK_ECOLX
MAKELSGYGPTAGESMGGTGANLNQQGGNNNSNSGVHWGGGSGHGNNGGQGNSNSSGSTS
TVMKTGESYLTPWGDVVINNDGLPVMNGIVMTEENSTLVDNPFGGVSRVLNSLISDMPSL
FAESSGNNNNNTASVNTAPTNAQVSDMDKSSKVVSNVINEKQKQKNKIATQISEKQKKIE
EMKKVFKHHSYHGITDLERDVDELQKKSNQLDADISKLNSYKNTLQSKIGDVNKQKEAEE
KARENAEVAEHETLNEEKQAVAEAEKRLAEAKAELAKAESDVQSKQATVSRVAGELENAQ
KSVDVKVTGFPGWRDVQKKLQRQLEAKQAEYSAVENELKNAVSFRDGKAAEVKEAEQKLK
EAQDALEKSQIKDAVDTMVGFYQYITEQYGEKYAKIAQDLAEKSKGKKIQGVDEALAAFE
KYKNVLDKKFSKVDRDAIFNALESVNYDELSKNLTKISKSLKITSRVSFLYDVGSDFKNA
IETGNWRPLFVTLEKSAVDVGVAKIVALMFSFIVGVPLGFWGIAIVTGIVSSYIGDDELS
KLNELLGI
>AAD35867.1
MVNMEFLKRSFAPLTEKQWQEIDNRAREIFKTQLYGRKFVDVEGPYGWEYAAHPLGEVEV
LSDENEVVKWGLRKSLPLIELRATFTLDLWELDNLERGKPNVDLSSLEETVRKVAEFEDE
VIFRGCEKSGVKGLLSFEERKIECGSTPKDLLEAIVRALSIFSKDGIEGPYTLVINTDRW
INFLKEEAGHYPLEKRVEECLRGGKIITTPRIEDALVVSERGGDFKLILGQDLSIGYEDR
EKDAVRLFITETFTSRLSTRRP
>CAA44310.1
MPGFNYGGKGDGTNWSSERGTGPEPGGGDKGHSGDRDRGGAGVGNSPEQQQIAAIQNDPA
LRMKLEAVIKAARRINPDAKLHIESVSPSGTLSLSATGLTADQAKHIGLGGLVMGVNAKG
VTVAIGDIETGHARKPSPPGKGGNNGLNAGQIGASSLGSFVTDSHRDRPVSGWHGNGKTG
EFSTTRTTGSYYGFHHLKVEKQDGLATYSLYYKANKNRPAFIAVVRGDNLNAMEVKYANG
KPVKSPGSVKTIVKEFVEYQNAELKAIKDGVSLAAGINKDIAEKIGAKYAKLAKDLEAGI
QGKYIRNVQDAEKTYEQLTKGLNKKLKAQDKAAIVAWLKMIDAEQYARNARVLGKVFTGV
DWAIKGADLVNAAIEGFSTGNWKAFRNQLEALGLSIGAGYTLSAIAAFFAPTLVSSTVGI
FAFAYLFGWATSYIDAERAGELEKWVADL
>CAA72509.1
MPGFNYGGHGDGTGWSSERGDGPAPGGGMQGNGGGHSGNNDSGSNSVSQQISAIQNDQKL
KQKVVNMLIAARKMNPDAKMILGSIAPSGVMQVTIEGVTSTQARQLGLGGLVMGYNASGV
IGAVGEIDTGHRLNASGASTPGSETSVDSFVNGQKPAEEWHAVAKDSWTGAGPVNTGLVN
NAIKSVRIIKKGYVTGVLTPEEVMNKAEYKAMRQAFDSLPLAKQGEAVRQIVAAWSLAYQ
DFPVNLKKEMGRVTERIVDAINLALILNQTESRLSESQKNVDVANQIISDTVKAINDVNK
KIAEKRNQQVSLTDLMNKKQKEVEDLKKIFKNHSYHRIRDAQREYDDARNKYALLASDIN
ALQAQVSGLTARKQQAEQNKAAAEKAKADAAAKAAAEKAAAEAKAKAEAEKARKEAEEKA
NDEKAVLTKASEIIISVGDKAGEYLGDKYKVLSREIADNIKNFQGKTIRSYDEAMASVNK
LMANPDLKINAADRDAIVNAWKAFDAEDMGNKFAALGKTFKAADYVMKANNVREKSIEGY
QTGNWGPLMLEIESWVLSGIASAVALSFFSAIFGTFAMLGVFSTSLAGILAVILAGLVGA
LIDDNFVDKLNNEIIRPAY
>NP_061654.1
MVCFKSQRGMNMPGFNYGGYGDGTGWSSESGGPAPGGGMHGNSGGQRGDNANSSNSVSQQ
ISAIQNDQKLKQKVVNMLIAARKMNPEAKMILGSIAPSGVMQVTIEGVTSTQAKQLGLGG
LVMGYNASGVIGAVGEIDTGHRLNASGASTPGSETSVESFVNGQKPAGEWHAVAKDSWTG
AGPVNVGLVNNAIKSVRIIKKGYVTGVLLPEEVMNKAEYKAMRQAFDSLPLAKQGEAVRQ
IVAAWSLAYQDFPVNLKKDMGRVTERVVDAVNLALILNQISSGMSASQKDVDAANRIINE
TVKAINDVNLKIAEKKKQQVPLLSLMKQKQKEVEELKKVFKNHSYHRIRDAQRAYDDARN
KNDLLVSDINALQAQVSGLTARKQQAEKNKAAAEKAKADAKAKAEAEKAAAEAKAKAEAE
KARKEAEAKANDEKAVLTKASEIIISVGDKVGEYLGDKYKALSREIAGNIKNFQGKTIRS
YDEAIASVNKLMANPDLKINAADRDVIVNAWKAFDAEDMGNKFAALGKTFKAADYVMKAN
NVREKSIEGYQTGNWGPLMREVESWVVSGIASAVALAIFSATLGAYLLAVGASAAVVGII
GIIIASFIGALIDDKFIDRLNNEIIRPAY
>sp|P04480.1|CEA_CITFR

MPGFNYGGKGDGTGWSSERGSGPEPGGGSHGNSGGHDRGDSSNVGNESVTVMKPGDSYNT
PWGKVIINAAGQPTMNGTVMTADNSSMVPYGRGFTRVLNSLVNNPVSPAGQNGGKSPVQT
AVENYLMVQSGNLPPGYWLSNGKVMTEVREERTSGGGGKNGNERTWTVKVPREVPQLTAS
YNEGMRIRQEAADRARAEANARALAEEEARAIASGKSKAEFDAGKRVEAAQAAINTAQLN
VNNLSGAVSAANQVITQKQAEMTPLKNELAAANQRVQETLKFINDPIRSRIHFNMRSGLI
RAQHNVDTKQNEINAAVANRDALNSQLSQANNILQNARNEKSAADAALSAATAQRLQAEA
ALRAAAEAAEKARQRQAEEAERQRQAMEVAEKAKDERELLEKTSELIAGMGDKIGEHLGD
KYKAIAKDIADNIKNFQGKTIRSFDDAMASLNKITANPAMKINKADRDALVNAWKHVDAQ
DMANKLGNLSKAFKVADVVMKVEKVREKSIEGYETGNWGPLMLEVESWVLSGIASSVALG
IFSATLGAYALSLGVPAIAVGIAGILLAAVVGALIDDKFADALNNEIIRPAH
>sp|P05819.3|CEAB_ECOLX
MSDNEGSVPTEGIDYGDTMVVWPSTGRIPGGDVKPGGSSGLAPSMPPGWGDYSPQGIALV
QSVLFPGIIRRIILDKELEEGDWSGWSVSVHSPWGNEKVSAARTVLENGLRGGLPEPSRP
AAVSFARLEPASGNEQKIIRLMVTQQLEQVTDIPASQLPAAGNNVPVKYRLTDLMQNGTQ
YMAIIGGIPMTVPVVDAVPVPDRSRPGTNIKDVYSAPVSPNLPDLVLSVGQMNTPVRSNP
EIQEDGVISETGNYVEAGYTMSSNNHDVIVRFPEGSGVSPLYISAVEILDSNSLSQRQEA
ENNAKDDFRVKKEQENDEKTVLTKTSEVIISVGDKVGEYLGDKYKALSREIAENINNFQG
KTIRSYDDAMSSINKLMANPSLKINATDKEAIVNAWKAFNAEDMGNKFAALGKTFKAADY
AIKANNIREKSIEGYQTGNWGPLMLEVESWVISGMASAVALSLFSLTLGSALIAFGLSAT
VVGFVGVVIAGAIGAFIDDKFVDELNHKIIK
>sp|P17998.1|CEAD_ECOLX
MSDYEGSGPTEGIDYGHSMVVWPSTGLISGGDVKPGGSSGIAPSMPPGWGDYSPQGIALV
QSVLFPGIIRRIILDKELEEGDWSGWSVSVHSPWGNEKVSAARTVLENGLRGGLPEPSRP
AAVSFARLEPASGNEQKIIRLMVTQQLEQVTDIPASQLPAAGNNVPVKYRLMDLMQNGTQ
YMAIIGGIPMTVPVVDAVPVPDRSRPGTNIKDVYSAPVSPNLPDLVLSVGQMNTPVLSNP
EIQEEGVIAETGNYVEAGYTMSSNNHDVIVRFPEGSDVSPLYISTVEILDSNGLSQRQEA
ENKAKDDFRVKKEEAVARAEAEKAKAELFSKAGVNQPPVYTQEMMERANSVMNEQGALVL
NNTASSVQLAMTGTGVWTAAGDIAGNISKFFSNALEKVTIPEVSPLLMRISLGALWFHSE
EAGAGSDIVPGRNLEAMFSLSAQMLAGQGVVIEPGATSVNLPVRGQLINSNGQLALDLLK
TGNESIPAAVPVLNAVRDTATGLDKITLPAVVGAPSRTILVNPVPQPSVPTDTGNHQPVP
VTPVHTGTEVKSVEMPVTTITPVSDVGGLRDFIYWRPDAAGTGVEAVYVMLNDPLDSGRF
SRKQLDKKYKHAGDFGISDTKKNRETLTKFRDAIEEHLSDKDTVEKGTYRREKGSKVYFN
PNTMNVVIIKSNGEFLSGWKINPDADNGRIYLETGEL
>sp|P09883.4|CEA9_ECOLX
MSGGDGRGHNTGAHSTSGNINGGPTGIGVSGGASDGSGWSSENNPWGGGSGSGIHWGGGS
GRGNGGGNGNSGGGSGTGGNLSAVAAPVAFGFPALSTPGAGGLAVSISASELSAAIAGII
AKLKKVNLKFTPFGVVLSSLIPSEIAKDDPNMMSKIVTSLPADDITESPVSSLPLDKATV
NVNVRVVDDVKDERQNISVVSGVPMSVPVVDAKPTERPGVFTASIPGAPVLNISVNDSTP
AVQTLSPGVTNNTDKDVRPAGFTQGGNTRDAVIRFPKDSGHNAVYVSVSDVLSPDQVKQR
QDEENRRQQEWDATHPVEAAERNYERARAELNQANEDVARNQERQAKAVQVYNSRKSELD
AANKTLADAIAEIKQFNRFAHDPMAGGHRMWQMAGLKAQRAQTDVNNKQAAFDAAAKEKS
DADAALSAAQERRKQKENKEKDAKDKLDKESKRNKPGKATGKGKPVGDKWLDDAGKDSGA
PIPDRIADKLRDKEFKSFDDFRKAVWEEVSKDPELSKNLNPSNKSSVSKGYSPFTPKNQQ
VGGRKVYELHHDKPISQGGEVYDMDNIRVTTPKRHIDIHRGK
>AAG29099.1
MKNILLSILGVLSIVVSLAFSSYSVNAASNEWSWPLGKPYAGRYEEGQQFGNTAFNRGGT
YFHDGFDFGSAIYGNGSVYAVHDGKILYAGWDPVGGGSLGAFIVLQAGNTNVIYQEFSRN
VGDIKVSTGQTVKKGQLIGKFTSSHLHLGMTKKEWRSAHSSWNKDDGTWFNPIPILQGGS
TPTPPNPGPKNFTTNVRYGLRVLGGSWLPEVTNFNNTNDGFAGYPNRQHDMLYIKVDKGQ
MKYRVHTAQSGWLPWVSKGDKSDTVNGAAGMPGQAIDGVQLNYITPKGEKLSQAYYRSQT
TKRSGWLKVSADNGSIPGLDSYAGIFGEPLDRLQIGISQSNPF
>AAT85004.1
MADNQPVPLTPAPPGMVSLGVNENGEEEMTVIGGDGSGTGFSGNEAPIIPGSGSLQADLG
KKSLTRLQAESSAAIHATAKWTTENLAKTQAAQAERAKAAMLSQQAAKAKQAKLTLHLKD
VVDRALQNNKTRPTVIDLAHQNNQQMAAMAEFIGRQKAIEEARKKAEREAKRAEEAYQAA
LRAQEEEQRKQAEIERKLQEARKQEAAAKAKAEADRIAAEKAEAEARAKAEAERRKAEEA

RKALFAKAGIKDTPVYTLEKTKAATTLFLTPGVRLLNRAPAMIQLSALAAEINGVLTTAA
SAVMTATAEFSGWIASALWRGVAGVATASTVGPMVAAASTLFFSPRAGGGSDSKVPGRDI
EMLAAQARLFTAGKLSIEPGMKSVNLPVRGFISSETDGRQSLMLVKTGSDGVPSTVPVLD
AVRDSTTGLDKITVPAMSGAPSRTILVNPVPIGPAAPWHTGNSGPVPVTPVHTGTEVKQA
DSIVTTTLPIADIPPLQDFIYWQPDASGTGVEPIYVMTSQPRKGVKDYGHDYHPAPKTEE
IKGLGELIESRKKTPKQGGGGRRDRWVGDKGRKIYEWDSQHGELEGYRASDGSHLGAFDP
NTGKQLKGPDPKRNIKKYL
>AAL73547.1
MKTNNVTGTMKKVISTLAATGCMFSMAAAIPANSTIGSAVLGNAVVADAAVISVNTVVDA
KNGNADLVQGKFYKSPSQNYVLVFQNDGNLVIYHYNKTTDKAYSPIWSSQTENRGGTKCV
LQGDGNFVIYRSDGKPIWNTQTNGKKGAYLTISDEGEIKITSRNYNYATTWSSKNNHGYS
INQGPIITDPVDGQLSPHFHSREFACDCGNTHTIDQNLINKLEQLYTKLNCSKIIVNSGY
RDPNCSVAVGGGYDDAHTRGLAADVVCYDKNGNVIPCLTVAWAAEQIGFTGIGLMYGGAI
HLDVRTTSNYKNGHWFGDERKEYKNDYISTFKNYVPHKA
>AAT90329.1
MSDITYNPEDYNNGIPPEPGLVWKPGGSFPNGSYVPGSWGWPTRGYDVPPLPGDTEMLTV
TPKGTPADTWPKRPDIKEWYVPGEKPFDPSTGNGWVPDVDGYAESLPAGIPAVVQAAISK
VKGAPLKGGMSAVDIWKLKPATEYPGRFNSTDPAFSWFPVRALTDTDISAMPVAPETVPV
HTRILDNVHDGVQFVSAVFAGSMQYNLPVVKAQATAGSDYYTIGRLPGIMSAFTFSFYTK
GTPQDSRFFRDTVKAGGDLREAGFTVGANTSDFIIWFPQGSGLEPLYFSMTMNMPAGPLQ
RRQEAENKARAEADRLRAEAEAKIRAEAEARAKAEAERKALFAKAGIQDTPVYTPEMVKA
ANAALSAGGSMALSRAPGMIQHSAAGVGTLPFNSSLAGWEAGALWRGVDVLARIAPVASA
VATVATVLTLVRAALDIPAAGEGSDRVPGRNIDMLAAQASLYTAMKTNIQPGMKTVDLPV
RGYISYDGNGRQSVNLVRTGTGGVSATVPVLSAVRDKTTGLDKITVPAVAGAPSRTILIN
PVPVGPATPSHTGSSTPVPVTPVHTGTDVKQADSIVTTTLPAADIPALQDFIYWQPDATG
TGVEPIYVMLSDPLDSGKYTRRQLQKKYKHAIDFGITDTKINGETLTKFRDAIEAHLSDK
DTFEKGTYRRDKGSKVYFNPKTMNAVIIQANGDFLSGWKINPAADNGRIYLETGDL
>NP_889019.1
MNNLYRDLAPISAAAWAQIEEEVARTFKRSVAGRRVVDVKDPGGFGLAAVGTGHLRGIAA
PQKGVDAKLREVKALVELTVPFELQRDEIDAVERGANDADWQPAKDAATELAYAEDRAIF
DGYKAAGIVGIREGSSNSRLELPTDAADYPAAVGRALEQLRLAGVDGPYSVLLGADAYTA
LSEGSDDGYPTIDHIKRIVSGDIIWAPALNGGCVLSTRGGDFELHLGQDLSIGYQSHTDK
VVRLYLRETLTFLMLTSEASVPVAPKG
>CAE09438.1
MDILRRENAQFPASIWSAIEKEAGLVFGKHLTGRKVVDFKGGLGIGFSSLPTGRVISSKE
KLGEASVGVRMNTPVIELKIPFSFPESEVEAILREANAFDISSIEKAAKKVCVAENELVF
YGLKKEGIEGLIPSIPHKPIKAKGDEILPAVAEGIKELVNSEIEGPYALLIQPQYFGKLF
GVAGNSGYPLTLKLAELLQGNNIIVAPALKSGALLVSLRGGDYELYSGMDIGVGYSEKKS
TNHELFFFETLTFRINTPEASIAIEW
>YP_426062.1
MNDLMRDLAPISAKAWAEIETEARGTLTVTLAARKVVDFKGPLGWDASSVSLGRTEALAE
EPKAAGSAAVVTVRKRAVQPLIELCVPFTLKRAELEAIARGASDADLDPVIEAARAIAIA
EDRAVFHGFAAGGITGIGEASAEHALDLPADLADFPGVLVRALAVLRDRGVDGPYALVLG
RTVYQQLMETTTPGGYPVLQHVRRLFEGPLIWAPGVDGAMLISQRGGDFELTVGRDFSIG
YHDHDAQSVHLYLQESMTFRCLGPEAAVPLRGLSQAATKA
>YP_366690.1
MNNLHRELAPISSSAWEQIEEEVARTFKRSVAGRRVVDVDGPEGPELSAVGTGHLVEVAA
PREQVNARLREVRTIVELTVPFELSRDAIDSVERGARDADWQPAKDAAQRLAFAEDGAIF
DGYAAASIVGIREGTSNNKLTLPADVSAYPDAISDALEALRLAGVDGPYSVVLGSDAYTA
LSEARDQGYPVLGHIKRIVSGEIIWAPAISGGCVLSTRGGDYELHLGEDVSIGYTSHTDK
VVRLYLRETFTFLMLTSEASVAVAPQANTTA
>sp|Q45296.1|LIN18_BRELN
MNNLYRELAPIPGPAWAEIEEEARRTFKRNIAGRRIVDVAGPTGFETSAVTTGHIRDVQS
ETSGLQVKQRIVQEYIELRTPFTVTRQAIDDVARGSGDSDWQPVKDAATTIAMAEDRAIL
HGLDAAGIGGIVPGSSNAAVAIPDAVEDFADAVAQALSVLRTVGVDGPYSLLLSSAEYTK
VSESTDHGYPIREHLSRQLGAGEIIWAPALEGALLVSTRGGDYELHLGQDLSIGYYSHDS

ETVELYLQETFGFLALTDESSVPLSL
>CAA90860.1
MSDTMVVNGSGGVPAFLFSGSTLSSYRPNFEANSITIALPHYVDLPGRSNFKLMYIMGFP
IDTEMEKDSEYSNKIRQESKISKTEGTVSYEQKITVETGQEKDGVKVYRVMVLEGTIAES
IEHLDKKENEDILNNNRNRIVLADNTVINFDNISQLKEFLRRSVNIVDHDIFSSNGFEGF
NPTSHFPSNPSSDYFNSTGVTFGSGVDLGQRSKQDLLNDGVPQYIADRLDGYNMLRGKEA
YDKVRTAPLTLSDNEAHLLSNIYIDKFSHKIEGLFNDANIGLRFSDLPLRTRTALVSIGY
QKGFKLSRTAPTVWNKVIAKDWNGLVNAFNNIVDGMSDRRKREGALVQKDIDSGLLK
>YP_050090.1
MFTDEIIWHDVITKYSVNNLSQDMLNDPSETMFVLGDVYKEQALEYYGYLRSELLKSKEL
ISNAEKSLIIALESRVKAEQDKKSADQKLKDEQEKDKGKAPELKLDDKIREQLGNRGWTE
QDVRDTVSKGAKGSAEDKCSPKKTPPDFLGRNDPASVYGEFGKYIVVNDRTGEVVQFSDK
SDPEWVDDSRINWGDKNE
>AAM95702.1
MAGRTRIPFNGVGTSVLPAYQTLSAGQYLLSPNQRFKLLLQGDGNLVIQDNGATVWVANE
QQPFSSTIPLRNKKAPLAFYVQYGAFLDDYSRRRVWLTDNSTFTSNDQWNRTHLVLQDDG
NIVLVDSLALWNGTPAIPLVPGAIDSLLLAPGSELVQGVVYGAGASKLVFQGDGNLVAYG
PNGAATWNAGTQGKGAVRAVFQGDGNLVVYGAGNAVLWHSHTGGHASAVLRLQANGSIAI
LDEKPVWARFGFQPTYRHIRKINPDQKPIDIWTWHF
>prf||1912296A
MSDVFDLGSMTTVATATGQYSFYTPPPPTPIPYLTYIARPGINKFDLPEGAKIKDLIKRY
QYIGSQIPAAIMIRGVQEEIKKSTNTALANVGAIVDGELAYLASQKKEKLNPAEATPLQM
ASAEKAAAVELLASKQKELADARTIANAFFGYDPLTVNYVNVMNEIYGRREDKDFSFDNW
SKSYSAAQKIRLIEAKISVLNSRSSALDGKVAELTRLQRLEDAQHAAEAARQTEAERLAQ
EQRQAEARRQAEEARRQAEAQRQAELQRLAEAEAKRVAEAEKKRQDEINARLQAIVVSES
EAKRIEEIYKRLEEQDKISNPTVTTPPAVDAGSRVDDALAHTGTRVTSGGETGATGGSGR
DVDTGTGQGGITARPVDVGSVSIPDRRDPKIPDQPRRDLGSLVPTFPDFPTFPSFPGVGV
PAAAKPLIPAGGGAASVSRTLKTAVDLLSVARKTPGAMLGQVAAVVATMAVSSFWPKLNN
GERQASFAIPVAELSPPLAVDWQAIAAAKGTVDLPYRLKTLNVDGSIQIIAVPTEPGSAA
VPVRALTLDSASGTYKYTTTGPGGGTILVTPDTPPGQIDPSSSTPAVPRGPLIMPGTLLI
PKEPQIESYPELDQREFNDGIYVYPEDSGIPPLYIVYRDPRDEPGVATGNGQPVTGNWLA
GASQGDGVPIPSQIADQLRGKEFKSWRDFREQFWMAVSKDPSALENLSPSNRYFVSQGLA
PYAVPEEHLGSKEKFEIHHVVPLESGGALYNIDNLVIVTPKRHSEIHKELKLKRKEK
>AAA23073.1
RFAHDPMAGGHRMWQMAGLKAQRAQTDVNNKQAAFDAAAKEKSDADAALSAAQERRKQKE
NKEKDAKDKLDKESKRNKPGKATGKGKPVGDKWLDDAGKDSGAPIPDRIADKLRDKEFKN
FDDFRRKFWEEVSKDPELSKQFNPGNKKRLSQGLAPRARNKDTVGGRRSFELHHDKPISQ
DGGVYDMDNLRITTPKRHIDIHRGQ

Non-bacteriocin
--
>WP_001030800.1
MNKDSTQTWGLKRDITPCFGARLVQEGHRLHFLADRAGFTGSFSEVQTLQLDEAFPHFVA
HLELMLLSCELNPRYAHCVTLYRNGLTGEADTLGSHGYVVYIAILNRHGFNRHLRVI
>WP_050443533.1
MNFEQMKAVYEMVKAIYNKEERLVIGKEKLHLTHGINKNSFADFYRAFQKMLDGELHTRG
ISTDLRDFYLSQIYEDYGTKKLETALNAYMDFIIYYEKKHNNIKKKNERKIYQKHYELIK
HQSPERKGRVKVVEFYEGEFEQVFITKHERNTEARNKCIQAKGVKCVVCDFDFEKTYGEL
GKGFIHVHHINPISTKDGNYAINIENELVPVCPNCHAMLHRRKDKILSIEELKRIFHNK
>WP_159117600.1
MAKDTPSMAKNKIKRCLWAIYDQHPKKSEVDSLWTYFESKCAYCGVEIERSSRTGHVDHL
IPSAEGGSNSIHNHVLACARCNGDEKREEDWLTFLSKKSGKSSIFEQRRSNIEEWLSLMP
PNGTNTALKSEVEKVVDKALKDFDSAVAQVRSLINVNDRG
>WP_130071231.1
MSRNPYYIKMINSQRWKNLRCDKLRANPVCEVCEANGLSTLATEVHHKSPVESVSHELGM
KHLMFDRTNLQSLCHACHSEIHRRVFSHSKEAIQANNRRATERFADKFLK

>WP_169167565.1
MKVLKLSAQGVPQSWITLEQAVIHSAAGDVRWVAGSEVAVFHGGHNAVTGLQSVIAVNSI
IGTRGVSRINPFELKPGLANNKLFARDRNVCAYCGGHFDEHDLTREHIVPLAQKGADQWM
NVVTACRPCNHRKGPRTPEQARMPLLYAPYVPSLWEDFILRNRRILADQMEFLAAHLPRS
SRLLN
>WP_160213701.1
MALFLLEWWRMAKPFSDAFYHSKAWGRAREDALKRDSYLCQRCLAGGEITPATMVHHIEE
LTPANIDNPDITCGLDNLVSLCDLCHKKTHGWARAGATRQGLAFDADGNLICLAE
>WP_160212293.1
MPSNNVRYRNWKARTEQRRRILRECDGRVCPFCGRPMDASLDWWTDPADGRRKRHPYSIE
VDEIVPVSKGGSPIDPANLQGAHRICNQRAGAKNRRPKPRGDVTGGGLPASREW
>WP_159494819.1
MLVLRLNKAGMPQEWIDVEHAAKLYSQEKVLFELGSDAITLKGGWNHEGLQSQLTLSSII
ACDGKVTDMSGKVALTNRFLFRRDSYLCLYCGQKFSPKQLTRDHIIPRSRGGKDTWTNVA
TACQRCNHAKAAKTPEEANMPLLAVPFRPNIYERFYLMNRRILSDQMAFLKGHFSHKRNW
TCLD
>WP_142428358.1
MIDVTSKQARAKFYGSSEWRRLRQQCLERDHYECQWCKQEGKLTTQYDSVLEVDHIKELE
HYPQHALDIDNLRTLCKDCHNKRHGRFNYRESKRKRKWDDEWW
>WP_142426822.1
MIEVTTKTDRAKFYSSSQWKKLRLKALERDHYECQWCKEQGKVTTINDAILEVDHIKELE
YHPEFATDIDNLRTLCKECHNKRHSRMNYRGAERKKKFDDEWWGD
>WP_142422844.1
MTDEFYRWLLQLIREDRLVKFYQSPKWRRLREKAMKRDHYECQECRRLGKYHRVENVHHI
KEVKDRPDLALDLDNLICLCVEHHNEVHGRYLTALDKQEKKIESFANFDASERW
>WP_142422626.1
MGKKWNTEMFSEFVNSTYPDFEVRGEYVSSKNNILIYHKKCDREFSVIARNFKTRGTCSL
CNGKFKSNTSEFKDKVNTLTNDEYEVIGEYVTCKDKIELAHKKCGTIYFATPDDFINGGT
RCPRCFGNNRKTSKRFKNEVFNLFKNEYIVLGEYKNNKTPLLMKHDSKKCNHEFMVSPDA
FLRGSHCNKCGTEKRSGENHYKYNFSLTEEDRMARDMQNGEIRKWRDKIYLRDDYTCQVC
RIKGYKLNAHHLNSWDFYERERFDTDNGITLCEDCHRKFHKKYGYGHNTKKQFTLYLEEN
KPTTSIL
>WP_121698255.1
MSERRISEKELILPTLYLAVCNGGRITTSELIKQLTAMMRPSGIDAEILSGRNDTYFSQK
VRNLRSHNTLVAPGYAIYDDKGYAITQLGRDFVEARMDSLRYLLSSDFDYEDVRGHLDDV
TDGKVIPYDELVSEGETITMTATSHERSRKLRDAAVAHYTQDGVLKCCCCGFDFGSFYGD
KYGSSCIEIHHIKPIFMYEGRSEEQTIEEALDNLMPVCPNCHRAIHRNHVMRDELPDFIA
AIKASRKS
>WP_081722951.1
MERGSPHTWEVVDVATSRTGTAQYKHWRKRVLIAARDAGIAQCPHCGVRLDYTRGLQPNS
AEPDHILPVRWGGKNTLENGRVLCRRCNQSRGDGTRPKVKPRRAASVDVDW
>WP_001372261.1
MIEKICEVIDGEYVCDIDISVEEWKILLRDKKVFDDKSIAALKKWFIEPDHSCTCFDIGK
KYDLHSMSANGVINGLGGRVQKQLGRFEVKGVGKIASGTKFITVMKSREIKGNPKRNLWT
IREELVQAIKELDFFSTNESSSIDFYSDNDLITALEESNHFDVTQTFEYSEKAKPKKAAI
EVKNGLSYPRSKSVSKNALNKADYKCEINCDHPTFRRRNSPLNYTEPHHIVPMSKQDYFE
NSLDVEENIISLCCNCHKQIHLGKGFEDMLRKIYAERKDVLKKAGIEILLEDLILFYKME
GN
>WP_102372778.1
MSGNSRNAAQPEFREGSRCEVTLDRYERSEAARKACIAAHGATCAICGFDFSHTYGPTFA
GIIQVHHIVPLHVTGKEHEVDPMHDLIPVCPNCHVALHSKPDGTYLPDELRALMR
>WP_160214295.1
MFGFSVTLYLFIAGMGAGLYIASCLVEREMERARPPRDMQLLHQKALIISLLLVCAGSAF
LILDLTVPQKMYLVFKRPFGSVISFGAWLIALLTLMLAVRNGFWRIFATSRSPLIRFLKA
ATFLLACGVTLYTGFFLIGLKAISFWESLLVVALFAISSLSSGIACFSVLAAFSLRRSTT
PPVVCKADQVDTFLLAAEIIVLGIFVVSQLFGDAASAASSSRLISGELAWAFWLMLVGIG

LLFPFGLSVFGRANHKLSPLVVKGISACIGCFFLRYCIMEAGVRSFSLA
>WP_160213053.1
MTYEPIWGPIIAWYLFLAGLGGGAFVTSVFIRFRHPECTRLIRTGRIIAPAVVIIGLCLL
MFDAHAGFMHPLRFALLLTNFGSVMTWGVVFLAAFVVLALVALLLDLLKKPVWQWLDIAG
MVMGLCVAIYTGCLLGVCQGFPLWNNALLPVLFLVSAVSTGMAAVLLAGVFVAPEEFNAV
VSLKKFHFWLPVVEIALVMALLFITASNPSPAGWNSVVTLLCGDWAVAFWVLFIAVGLVI
PIALECWMLWIATPVVEESRTGQMISGFSDLGVLVGGFVLRLMIVSAALPITIVQPWIF
>WP_160213014.1
MQLLTKNKLLTGIFAVLGVAGIAAWAYQLAGGLGVTGMSNANSWGLYIAMFMFFVGLSAG
GLIVASSASVFHTTEYKKVAMPAIILSTVCICCAGAFVLIDLGGIQRIWHLFASPNVASP
LVWDICVITLYLVINVLYLRFMHKGAERAVSVLSRFALPTAVLVHSVTAWIFGLQIAKEG
WFSAIMAPIFVASAMDSGLALLLIVLIALNAAKLFETPKKLIASLAGLLATCIAIDGFLI
FCEVITMAYPGAEGAATLAVMVSGPTAPFFWAEVVGGLLVPFLMLVFAKNRQNTALVTVA
SVLVVAGVLCKRLWLLLTAFVIPNIVGAPGIMSDAWMMGGSYAPTAIEFLIVLGVPSLGA
LAFMAIGSKLLVPATAKEHAPARSGAAADLDLEAQVA
>WP_160213011.1
MSDLIAAYLFCAGAGSGAAFLAAVFECFVRAGAFRRARFADRRQAVSMRAVALSVYGAAL
VLLAFGMLCLVFDLGRPDLALKLFLRPNLTLSTFGAFALAVLALALMVLVALRLGRENQG
AIRRRIDGLSRAVVIVASAAVMAYAGLLLGQADGMPLFETPWLAVLFVASALASGLAVVM
LAVAVAGNGHVEAVRYLKRRLTVRLDVALIVLEAVAAGLYLAAIALGPAGTVALAPLLTG
AQGGLFVGGFGLGGLTVPLVLDLLQWRRPLPGWAYGLAAVATLLGALALRFALVQAAGPL
LSWAPVA
>WP_160212856.1
MLDTFVTVYLFLGGCAAAVVLVTCAWSLAVRAACGRRQPAPPVFGRLRVRCLLAGFVLLV
LAVLCLLLDLGRPQLFWLLFARPTSSLISIGSFLLMATLLVSGFLLGASVPGAPRSSRRV
LCSAEVVCCALSAGVMLYTGLYMACLEAVPLWNNPALPVLFALSSLSSGLSVVLIAASFA
DDRFLLAADCRRLRLAHAVSLAGEMVAVGAYLALAWGDGFARPGLEALLSPNDLGSWFVV
GFLGLGVALPLGAEVFAAMARRPMEAIPLDALCIIGGLVLRFCVVIAA
>WP_160212813.1
MFDALVIAYLFLGGTGAALGGLLGLLTLGQVLGLGESGRGQHLNGLSSGQHRRFFGFGNV
LAAAVCLLGAVCLLFDMERPDKVLVLLTSPNTSLVAMGAYSLGAVLLLSALAGVLHLHRR
TLPPAAGALLCAAQLVAACVTMTYTALLLMGFRAVAFFQTWALVGLFFCSSASCGLALAT
LTGMVLRVFPLERGHERTAAAEAALSLLEGLFLVLFMIHAHYAAPQAFAQLATGPLAWAF
WTVVVGCGVASPVAAWALPRFVRGYRLHGGLSPALVLLAGFALRFCVIAVV
>WP_160212742.1
MDFNEGGREAASAAGRSAERAKEKAQAWGGAALNAAIGVSGVLAVLGIVLWGIQLSGGMV
QTAMRNLDSWGLYITMFMFFVGLSAGGLIISSVPKAFGIKGFGGISKVAVYSSIACTVAA
IGFVVVDLGQPMRLWELFVYSNLGSPLMWDIIVLGTYLILSCVYLWAQVQSEKGKVSAAA
LRVISVIALVCAVLVHSVTAWIFGLQVGREMWHTALLAPWFVSSALVCGTGLVMLVCMGL
SKAGYLEFSRENLVKLAKLLGAFVCVDLYFFGCDLLTEAFPAAGGMEVVTMLVSGPLAPF
FWVEIIGCILCAAVCFVPSLRKPGLLAVGAVLAIAGIFCKRVQLLVGGFQLTNLDMPGPV
TSLSVTNWESGFSGAYSGLVYWPTPLEFGVALGVVALAVFIFCLGVKFLPLRPKED
>WP_068921157.1
MSVQADSVVAHRWALRSGVYRATAANGDLMLAAWPHTAMLGHASPQLLALLDALAEGPVP
VDEPGMSATLDRLRAGGWLSRTVSCAGRDLYTVTPLAAPTEAPAPAGELRLSRFAVLRNT
PEGLVLEMPGSWCDIRVHDPAVAALLADPSGDAGLPADAAAAVRADLVAAGMLVAEEEER
EPFERRQWSTHELWFHERSRLGNRGWFGGAHFGGTFWARGVHEPPPARPSPYPGEAVPLA
RPDLATLRRTDPTLTTVLEDRESVRDHDDDAPITAEQLGEFLYRCARVRLLRTIEGFEYS
SKPYPGGGSAYELEVYPIVRLAADLTAGMYHYDAHDHLLRPVQPLGHPSVRRLLKVATES
SVTKAPPQVLLVISARVGRIMWKYEAMGYALMLKHVGVLQQTMYAVATAMGLAPCALGSG
DDLAFTGATDRDRLTECAVGEFMIGSRRKELATWQL
>WP_121705446.1
MKRKTALLVAACAALMALGGCQKVNEAPTTEAAPQAETGAATKKDPAGEKAEEGKGVNKV
AYITAQRLGDDGPVDMVYRGIKAGCDEAGIEVHVVEAKKGEYEESMQAMVSEGYNLIFAV
FPELIDSVKAVSQQNPDVSFIHAICATKGDNLEGICCYEQQSSFVMGVLAAMTTKNNKVA
FVGGVDNPDTHRYLDGYKEGIEYVNPEIEVQTSWIGSFEDPAKAKELALVHYQNGADVLW

GSGGKSALGLYEAAKEMGEGYYVMGCTDDNNGRLPGQVLASHYEAWDTAAKDLVIDWNDG
IFEPGLKVLTLENGYAYCKLADESQCEIPQEVRDKVEEVTEQIKSGEIVVKSMPTYEEVI
ATLE
>WP_000146146.1
MASGDLVRYVITVMLHEDTLTEINELNNYLTRDGFLLTMTDDDGNIHELGTNTFGLISTQ
SEEEIRELVSGLTHSATGKDPEITITTWEEWNSNRK
>WP_007792748.1
MSEVTRYVVTVKFHEKSLTDINELNNHLTRGGFQLTLADDDGKIHELGTNTFGLVSALSE
KEVAELAEGLGEAALDQKPQVTVTTFENWLRDNDTV
>WP_188061558.1
MRPVKKLAVTSAVAMLSLGMTACGSNSTNNNSSAPAGNSGASGSSGGSAAGALKVGLAYD
VGGRGDHSFNDSAAKGLDEAKAEFGIKPTEVAATNGENDAARVSRLQQLAQSGNQAIVAV
GFSYAAAIGKVAKQFPNVKFAIIDDASPDSKGDNIDQITFTEEQGSYLAGAAAALKSKSG
HIGFVGGVEVPLIKKFQAGYVAGAKKVNPNIKIDSTYLTQAPDFSGFADPAKGKTAAQGM
FQNGADIVYHAAGKSGDGVFDAAKAAGSGKWAIGVDSDQAQTAPAGVRPIILTSMLKGVD
VGVKSFLKKVHDGNFKGGNSVYALKDGGVSLATTGGHIDDIKAKLDELKKGIEDGSIKVP
SA
>WP_083706534.1
MTRAPVIGWTMLAGAALAASAGSLPIQLGFAILAIGILGMAHGASDLAIVAPGRRPLFLF
LYVSVSLICLAWWTGYPEIALPLFLAASAIHFGVEDAPHGSLPERAARGISLVATPAILH
REGYGDILAFAAGHGISTTVLFLLIAAGAVATALVLIMAIRRRDGRLLIGTGALLVLPPL
IGFSIGFLVLHALPQTDQRREEIGCVSHRAYFRAVAPILLAALLIAAAVGAFFVYREGTG
VRALFAGIAALAMPHLLVTPWFEGRAGRPVAYSCPAISGRAQHPQT
>WP_076714729.1
MDWMAFLIGFTIMSLASLAIYAKGSKTSPSLHHTLLHAAVPFIAATAYLAMAFGIGTLIN
IDGSVTYLARYADWSVTTPILLASLVLLAFHERGKMGEVGGYLTAIIVLDVLMIVTGLIS
SLALVPVLKWVWYLWSCAAFVGVLYLLWVPLRAMAAERGEALGTAYRKNVVFLTVIWFLY
PIVFLVGPEGLKIISDPTSVWAILIMDVLAKVVYAFYAAANLKTALHDHRA
>WP_056438944.1
MARLSIFPLAGAILFPGMPLPLHIFEPRYRALVSDAMARDRRIGMVQPSGEGDTPSLYQM
GCVGRIAEVEAMEDGRYNLVLEGVSLFRIVRELEVTTPFRQVEAELLPVIDEDLLSLGRR
ASLEQESKRFADLQGYAVDWDAVGRLDDESLVNGIAQIAPFDVAAKQALLEAPDLEQRAE
LIIQLMQFFGRHDGEDRVTLQ
>WP_056438392.1
MRIDLTPYRRSTIGFDRLFDLLEANSRAASAENYPPFNLERLADDRYRITLAVAGFARDE
IEITAQQNMLLVTGKKDDKAGSPNFLHVGIANRSFERRFELADFVFVEDARLNDGLLVID
LVREVPEAMKPKTIAIKTGQPLAAVEHHAGEADEAKAA
>WP_162547883.1
MKNILLALAASAAAIVGVAAPAAAQDKTKVCFVHVGSKTDGGWTQAHDIGRQQLQEHFGD
KIETPYLENVPEGPDAERAIERMARSGCALVFTTSFGFMDATLKVAEKFPDVKFEHATGY
KTAANVATYNSRFYEGRFINGQIAGKMSKTGVAGYIASFPIPEVVAGINAFLHGARTVNP
EFKLKVIWVNTWFDPGKEADAAKALFDQGVDVLTQHTDTTAPMQVAEERGLKAFGQASDM
IAAGPTAQLSAIVDTWAPYYIKRTQAVIDGTWSSAQTFDGLKDGILSMAPYTNMPDDVKA
MAMDTEAKIKSGELKPFSGPINKQDGTPWLKEGESADDGTILGMNFYIEGVDDKLPQ
>WP_015068113.1
MTEAVKTPYPRTFSHIGISVPDLEAAVKFYTEVLGWYLIMKPTEIVEDDSAIGEMCTDVF
GAGWGKFRIAHLSTGDRVGVEIFEFSNQENPENNFEYWKTGIFHFCVQDPDVEGLAEKIV
AAGGKKRMKAPRYYYPGEKPYRMIYMEDPFGNILEIYSHSYELHYASGAYE
>WP_169634528.1
MTNLQKRSLLQTVALTAVAVAALVGCGKKEEVAPAAAPGAEAPAKSEPLKIAFMYVSPVG
DGGWTYQHELGRRAIQEKFGDRIETSFVESVPESADSERVMRDMAGQGSKLVFATSFGYQ
EFVQKAAADLKDVKFEHATGYKTAGNVATYDTKTFEGAYLAGIVAGGMTKTKTIGVVASV
PIPEVVRNINSFVLGAQSVDPAIKAKVVWVNEWFSPPKESEAATSLINGGVDVMYQNTNS
PAVLKTAQERGVRAFGKDGDMSAFAPQAHLGSAVIDWTPYYTKVTQDTLDGKWEGGSFWW
GVKEGAMDLVKIADDVPQEIKDRVAKAKAGMKDESFHVWTGPIQDNAGKEVLPAGKVGDN
AFLTGIDFYVHGVEGKVPGAK

>WP_159120456.1
MEHYLSLFVRSIFVENMALSLFLGMCTFLAVSKKVKTAMGLGVAVIVVLGISVPVNQIIY
VNILAPGALAWAGFPEADLSFLNFLTFIGVIAALVQILEMSLDKFFPALYNALGIFLPLI
TVNCAIFGGVAFAVQREYNLTESVVYGVGSGMGWAIAIVLLAAVREKLKYADMPDGVRGL
GSVFMIAGLMALGFQSFTGIQL
>WP_159120259.1
MDLATVIGMLGAIGFIVMAMILGGSLSMFIDVQSILIVFGGTLFVILSQFTLGQFFGAGK
IAGKAFMFKIESPEELIEKIVEMADAARKGGFLALEEAEISNEFMQKGVDMLVDGHDIEV
VRETLSKDISMTSERHDFGASFFKGMGDIAPAMGMIGTLIGLVAMLSNMDDPKAIGPAMA
VALLTTLYGAFFANVICLPIAFKLSVRAGEEKLNQSLVLDGIVGIADGQNPRVIEGVLKN
YLAASKRGSAEEE
>WP_159120252.1
MSLERFPLFPLSAHLLPEGRMALRIFEPRYVRMVKQACAENSGFVMCMLNSNGDKETNKH
IHKIGTYAQVVDFDMLDDGLLGIKVAGSHLVEVSNIEAEKDGLRTGDCKTIPQWQCDLAP
QQIAPMDERLKEIFGSYEELAALYESPKFDNPNWVLNRWLELLPVDGSQKQHFLAQRECT
SLLNYLSGLIG
>WP_105932012.1
MRTIDLSPLYRSFIGSDHLASLIDAASRAEKQSTYPPYNIELLGDDKYRVTMAIAGFSKD
DVSIQVEENTLTITGTKKAETEDKESKERKFLHKGISERNFERKFQLGDHVKVLAADMEN
GLLHIDMERVIPEAKKPRQIEIGSRLLENQ
>WP_105930623.1
MTQEETQIKSIPAKAYSVLEEWMNSITHGLGLIAAIIGLVFMVYRADNPLALTTAVIYGS
TLILMFLSSTLYHAISHDKAKGWLKLFDHSAIYLLIAGTYTPLLLVSIGGVLGITMTAVI
WCLAIGGVAFKLVAQHRFPKVSVMTYLLMGWIALGLIYPLYLALPGAGLWLLVAGGLCFS
LGVCFYVAKKVKYTHAIWHLFVIGGCSCHYFSIYYFVF
>WP_018697113.1
MESLNIFIRSIFIDNMVFAFFFGMCSYIAVSKSVKTALGLGAAVTFVMVMTVPLNYLLYE
FVLKAGALSWAGLPDVNLDFLTFIVFIATIAAFVQLVEMAVEKFSPTLYSQLGIFLPLIA
VNCAIMGGSLFMQQKVDALELTSLWQSIVYGLGSGLGWWLAIVMMAAIREKTTYSQIPAA
LKGPGIAFIITGLMGIAFMIFSGIQF
>WP_005856736.1
MFILMPVIFVLGILAIALEDKIKINKAAIALFMAISMWMILMFDAYNIFVERSSTIFQEF
LTQNPEMASLPPHEQFINFISNRAIVYHLGNVSETLFFVMCSMLIVDIVDKHGGFRAVTG
YIRTPNKRKLLWYISFATFFFSALLDNLAAAIVIMAVLRKLVPDRTDRLKYACMVIIAAN
AGGSWSPIGDVTTILLWVGKNISAMHQISHVFIPALVNMLVPLTIAHFWLFKKGSTLRVL
SEEEQGDEYIPEIPNRSRRMIFVIGVLSLALVPVFQMVTNLPPFLGVLLGLVILWFYTDL
MYSKLHMHESQKLRISQLLPNIDLATIFFFLGILMAVGALETSGQLGIMSAFLDKHVHEP
YLISFVIGALSSCVDNVALVAATMGMYPIVEQVADLSPYAQFFVSDGGFWTFLAYCAVTG
GSILIIGSATGVTVMGLEKIDFMYYTKRFSILALIGYCCGAGVYMLLFA
>WP_169169348.1
MNALVGRGGLFDEFFKDVNPGFYVRPLHGDPLPTPGQMKVDVKENDSGYTVCAEVPGVPK
EDIQVSVEGNVVSLRAEVRQQDQQTEGEKVLRSERYFGAVARSFQLPADIDAAQCKAKYD
NGVLTLTLPKKQGGNAQRLSIE
>WP_159490586.1
MTEVKQQTKTISAKAYSVLEEWLNSITHGIGCIAAIVGLIFMLYRAEDKLALTTAAIYGS
TLILVFLSSTLYHAISHQKAKGWLKLFDHSAIYLLIAGTYTPLLLVSIGGVLGITMTAII
WSLAMGGVAFKLIAQHRFPKVSVMTYLLMGWIALGLIYPLYLALPGAGLWLLVAGGLCFS
IGVCFYVAKKVKYTHAIWHLFVIGGCSCHYFSIYYYVV
>WP_142437480.1
MIKKISRYEMLNEILNCVTHGLGFILSIIALIALTTKAANLKSSIHVIAYLIFGIAQVLL
FFSSTIYHSLMFTKFKRVFQIIDHSSIYLLIAGSYTPYCLLAIGGTFGWGLYSFIWTCAI
AGIVYKNITMSKENKIPKYSMITYVLMGVFAILIIEPLYKSIGLTGVLLLVSGGLFYFLG
TYFYRSKNMNFSHPIWHIFVILGATYIYFSIFLTT
>WP_131622846.1
MNTLLIIILVVLMGLVVVSLVRGIVAFLQSHKADIDAGGQRQQDMQLLQNKMMFNRIKYQ
ALAIVVVAIIISIAR

>WP_121708026.1
MVGNKENKKRMEGWDITSLEAEGLHKCIRPGVQDCRAVSVYRLNLKQGSRFTLESGELEM
NPVLIRGRAKLSGAGLDGELEKLDSFYIPGDTGVGLEALEDCVFYIGAAPCEGYGKPFVR
KFDLSLPLGDIHQIHGHGVGQREVFFTLNHQVEASRLICGLTWGANGAWTSWPPHQHEKD
LEEVYCYFDMDAPRFGFHISYLKSGEVEDIVAHTVRSGSMVLAPAGYHPTVASPGTRNTY
FWILAAHSHASRRYDLAVLDPVYADT
>WP_121707743.1
MMFKVKDPGSALTHFIAMLLALAAATPLLVKAARSPEQTHILALTIFIISMVLLYAASTV
YHTLDISPKVNQILRKADHMMIFILIAGTYTPVCMLVLGDYTGWMLLALVWGIAFFGILI
NALWITCPKWFSSLIYIAMGWVCILAFGKIIAALPASAFGWLLAGGIIYTIGGVIYALKL
PLFNSRFKNFGSHEIFHLFVMGGSLCHYIMMYAFVA
>WP_106897292.1
MGTYMREPINGLTHLFGAILSFVGLLAMVIKASTTADSTLTIISVIIFGISMTLLYAASA
TYHLVVAKAHVIAFLRRLDHSMIFVLIAGTYTPLCLISLNGMTGWVLFTIISAIAVAGVS
FKLIWFHAPRWLSTALYIAMGWIVVFFSSSLAPVLGTNGMALLIIGGLIYTVGAFIYWLK
PKFMNFKHFGFHEIFHIFILLGSLFHFLCVYLYVL
>WP_016292657.1
MQITIREPGSAITHFIGMMMAIIATAPLLVKAAMEPGAASLASLAVFMLSMILLYGASAT
YHSVNFSERAIKIFRKIDHMMIFVLIAGSYTPVCMITLGGKLGYTLLAVVWGIAILGMSI
KALWITCPKWFSSIIYIAMGWVCVAVFGPLWRTLPASAFLWLLTGGIIYTIGGIIYALKL
PLFNSQHTHFGSHEIFHLFVMGGSICHFIFMYLYVA
>WP_032850333.1
MENKRYNNVEEWANTLSHGAGILLGVIAGYFLLAKAAAGAEPKWAVACVTVYLFGMLSSY
VSSTWYHGSRPGKLKELLRKFDHGAIYLHIAGTYTPFTLLVMRHAGGWGWGIFSFVWLSA
IVGFILSFKKLKEHSNLETACYIAMGACILVAMKPLMDHLAEMGAGPAFWWLIGGGVSYI
IGAVFYSLRKPYMHATFHLFCLGGSIGHIIAIWLIL
>WP_121698083.1
MKHKAIFIRDDNGTEVSAQSPVIVSASRGTDIPAFYADWFFRRLEKGYVRWRNPFSGQDS
YVSFENTRFIVFWSKNPAPLLPYLPMLKERGIGCYIHFTLNDYEAEGLEQNVPPLSQRIE
TFRRAVEALGRGAVVWRFDPLILTDKINIDTLLEKIAHIANALTGYTEKLVFSFADIESY
KKVSRNLRQSCINYREWDEESMCEFASRLSTKNHDNWNLRLATCAERIDLSEYGIGHNRC
IDPELISRLTPHDAILQNFLYNAKTDNGQRKACGCILSKDIGAYNTCPHGCLYCYANTSS
ASAFANYKEFATNPLTDLII
>WP_149888968.1
MSLKLVAAVAAVASAFALSACGDKKEAAPAKPATPAAPAAQTEAAEPLKVGFVYVAPIAD
VGYTKQHDIGRIYAIDKVGKDKVTTTFVENVPETADAERVIRQMVADGNKLIFGTSFGYM
NYMQKLAKEYPDVKFEHATGYKTAPNMTNYNIRFYEGRYLAGMLAGGATKSNIIGYVAPF
PIPEVLQGINAFTLGAKSVNPNIQVKVIWTNAWYDPPKDTDSAKTLLGQGADILTQHTNT
SAVASAAEAAGKMVIPYNSDMKSVAPNAQIAALVLNWGPYYAKKIQQTIDGKWDPTPVWM
HYKDGAMSLEGVRTDKIPADIVKKMEEVKAKIESGEFHPFTGPIKTNDGKEAAKAGEVLK
DNQLQTMNYYVDGVIGKVPN
>WP_010714134.1
MTKNKLFHLTDKSNINSIRKHGLVGVKKAKDLLRRETGTDHTLFNQQTYSILNTYGWKGY
DLRCATFMFEEDSCLGYELLQLMNDDPYILEIEIDRLNKDKLFVFNTEIASHLLNYSKQD
QHRLAKFYWNTAIPYNTYVQNKDKVNDAFQLGNIMYQAEYVYFGEISPKYIKNYERGKKY
DSGRY
>WP_007273123.1
MLMRTDPFRDLDRIAQQVFGTPARPAAMSMDAWRNGDTFEVEFDLPGIKPDSIDLDVERN
VVTVKAERPALDKDLEMLASERPRGVFSRQLVLGENLDTENIEASYEAGVLRLRIPVAEK
AKPRKISITAPSQDREAIDP
>WP_006681612.1
MREVVEYLEDRGVEHLVHFTPITNLGGIKKRGILPRNEIDGFPDIVFEALDEVRLDERTD
MSCFSISFPNFLMMYRYRTKLWSREEDVALLFIPISVLSDLEYDQVVFCPSNAASRECRR
TDPQDLLGLAAVEKLFVEEMTTRSGVVFSRQSEDLPDFLTTNPQAEIQIAATIPWEKVSF
VVVNDYETKQSLLQSGIHRNVYAKWEVNNDSSLNVFKYPSYWRAWVDAAGDLHG
>WP_121699091.1

MENLNIFIRSIFVDNMIFAYFLGMCSFLAVSKNVKTALGLGAAVTFMLVISLPINYLLET
YVLRAGALQWLGPEYADVDLSFLSLIMFIAVIASLTQLVEMAVEKFSPSLYSSLGIFLPL
IAVNCAILGGSLFMQQRDFPDVWTACCAGAGWGLGWLLAIVAIAAIRERLQEYSNIPKPL
RGVGITFILTGLMGIAFMSFLGIKL
>WP_121699089.1
MNKQSNTYTIIYIVALVIIVGTALAFTALSLKPLQTANADADKMKQILASVHIAPAKSDI
ITDFDKYITDRFVIDAEGKRVEGDAFAINVSAQSKLPQAERKLPVYECTLTPGDVKYILP
VYGAGLWGPIWGYVAVNSDGSSIYGAYFAHQGETPGLGAEIEKPAFSDQFTGLNLFKEGA
FKPVNVIKAGQAPMNGEDYVDAISGGTITSKGVASMLDNCLSGYKTFLESLTNKGQ
>WP_167508517.1
MSGSSRLAKFRTAQADKQNWQCFYCGFPMWEGDLALPSEHRRLPIGLLDRFLCTAEHLEP
KMNGGKNRPDNLVAACRFCNQTRHKMRDVLSPAAYQQHVRRRIRARKWHPIECHRLFG
>WP_160582050.1
MPYKPKRPCSYPGCPKLTDGRYCGEHQKIVTAHYNKHERDPASKKRYGRAWKRIRDRYIA
AHPLCEECRKAGKVTPAEEVHHIRPLSKGGTHAEGNLMALCKPCHSEITAREGGRWERRR
>WP_135855810.1
MIEFIDDTEPWRPTTSGERLLFLLLANHKEARFLSEFLAGSGLRFARRHSDWSNFLVGAS
LLHLQQRLIKLGNDVREKSPSDRMDQLKELRARTCDIVQLTPIEYEGDFGELVQEVLVSA
EQMHKEPPQNLKRSVLRSSPSCYSCGRNFGSVYENDEDAKEGLRATADHVWPRALGGDST
EDNLLPACTSCNSTKGHLATWHMAWLQPIVFSDVDGEHAPPPVPREVQMALHMRAATSYA
RANGTTLRDAFLAIGPRDRPEKIDSEQGYDFFNMRVHNETRTMVKWIPG
>WP_120438463.1
MPGWNLKNGELQKCQISEDEYWSLFNFVFSDACMKRNTYKFGLIKSIMDNLFNCTQDDYG
NYRLSYSAIFEKFTINYWNLVLKYHLKQMRSDGRTEVSKIESILLAASEENDLIKTLDFN
SLSNSDRSKVVRQVSIACRKNVIGALYNDMEGKLYGFDLKEKGIMLGERAYDFMLKYKTE
LEKLNYYAWAKFMEKINEDDVLVKVLDKLELSTPKRDDLSVYREVLYEEFEACNCFYCGK
KLSLSNRGIHVDHFIPWSYVKDDKLWNFVLSCPKCNERKNNKIPSKKYLEIMLKRNEYMK
GVVDEFVEIEFKNYDSSQFMRLWKYAQLSGMKQFQQEFF
>WP_120438225.1
MESYILHVRSVKESMSREVINTYQLEEILPKIIDYEEENYYNNIGVDSNLEKDLLEDLQM
LDNKYKEITKSVIIRYQKIVEHIKQTRGRKCQICQYSFIMDNGNEYCEAHHIQYLSKNGS
QSSDNVILLCPNHHRMFHYAHDAVFVDDLVDGKRKVLIENVEHLIDFS
>WP_140970856.1
MKEYKTKQQKRKFYDSGEWKSIREQVKKRDNYECQECKRNGRVQTDTNEYSESAKRKKIQ
LVVHHIKELEYHPELALEKDNLETACVDCHNKEHGRFFEKKPNKWENDEKW
>WP_169253183.1
MKTLVLNAGYEPLSIVPFTRAVVLVLTGKATVLAAEDIPVRSEHMSLDQPSVILLTRYVR
PPSNRRVSLSRRGVLRRDGHRCAYCSKPAYTVDHVLPRSRGGANTWENLVACCRECNNRK
GNRTLGEIGWKLSFLPQEPRLGQLWMRGIDKPVEKWRPFLEYSSAA
>WP_083793542.1
MMRLFNKRQRRILAWVAGGQCTICQRPLNSNFHADHVLAHSKGGATTTDNGQALCAPCNL
KKGAK
>WP_040799872.1
MAVAQTRRARAARRRKRRVDAADNDLTAEQWKELKREWGGCAYCTATDTVLQKDCVQPIS
RGGSYTVGNVVPACGSCNASKSNSEVTSWMRRKRLDERAFLTQYVHVRQALGLV
>WP_007230052.1
MVELEIEVGDEIKNEDLVRMFGCGPQGGMRRSHATNTLVLTSKHVDNVYDDRWVADVFHY
TGMGLEGDQSLTYSQNKTLAQSASNGVEVHLFEVFKPKFYTYMGAVELAAEPYVENQKDQ
NGLERKVYVFPLRPISGGQPTLPFKKIESAIHNKQKMAHKLSDQELLNRAASKSSAGASR
SVETKYYERDPWISEYAKRRAGGKCQLCESDAPFISKAGEPYLETHHIEWLANGGEDSIS
NTVALCPNCHRKMHNIADNNDVVKLKSRNASYE
>WP_007225374.1
MSTFLLTWSPDKWGYENLQEYLDARKSEEFVQRWSSGRTKKIPIGSRVFLTKQGKGNKGI
FGSGHVTKEPAEEPHFNEEQLKLGKKALFVMVNFDQLYDPQSEIPITHSELQAFDSKVWD
SQSSGITIPEETASKLEQLWLERTGAVEISYADEVPKDNSLKEGAAKKIWVNAYERNPDA
RERCIRKWGLNCVVCNFHFEQCYGHLGKRYIHVHHLKPLAEIQKEYEVNPEEDLRPVCPN

CHSMLHRNKNSVLSIEELQTLVNMYSR
>WP_169253656.1
MSTSEYDSYRPPEPEGGRKKRRRGGRGGPSGPDGPRGRRGGRGGGWKNRGADGNREMPMV
EDVEFTSYYGRPIVKAPPWGDEISAYLFLGGLAGGSSLLGYGAQLTDRPGLRIASRMTAI
AATGIGGVALVADLGRPERFLNMMRVVKVSSPMSLGVWILSGFGVGSGVTFAIELDRITG
EKLLPLGPLRKVLHGLETPAAVESAFFATPLAAYTAVLLGATAVPTWNAAGRNGLPYVFV
SSASMAAGGAAMALAPVGQTGPARLLALAGTAGEAYAMSAMKKRMHPAEVDPMDDGEPGH
KLHRAEKLLIAGTIGTAVAEVGARVFAKKLGGGWKTRAVLRGLSVVSGAALAAASAYTRF
GVLEAGIESTKDPRHVVEPQRARLEERRARGITDDSITTGR
>WP_141265030.1
MLVKALTGSKRYWGWITLLLVLIGTGFTCYLWQLDKGLTITGMSRDVSWGLYISQFTFLV
GVAASAVMVVLPYYLHNVKAFGRITILGEFLAVAALIMCLLFVLVDVGKPMRILNMIFYP
TPNSMFFWDMIALNGYLLLNIIAGWHALEAEYKAVPPPAWTKVLVYISIPWAVSIHTVTA
FLYAGLPGRHYWLTAIMAARFLASAFASGPALLILLCYIIKRVSKFDPGREAIQKLAAIV
TYATIVSTFFIGLEFFTAFYSQVPAHGIYTLKYLFAGLDGHSRLVSWMWAFAILVVFALV
LLINPGTRTRDSYLQLACAAVFVSMWIEKGIGLVIGGFVPNPFERVTEYVPTLPEILIAL
GVWATGFLVLTFLYKIAISVKEETV
>WP_160582917.1
MKLKIKDPGSALTHFIGMVLAILAATPLLVRAAHTPGPLHIAALAVFICSMILLYTASTV
YHTFDISESVNRLLRKIDHMMIFILIAGTYTPVCLIVLGNPAGYRLLALVWGIAVLGILI
NALWINCPKWFSSCVYIAMGWVCVTAFREIVAALSPAAFGWLLTGGIIYTIGGVIYALKL
PIFNSRHKNFGSHEIFHLFVMGGSFCHYMMMYGYIAA
>WP_160582365.1
MLMPSIFGEDLFDEMMGFPFDNRFFARRNPVYGKAATAVMKTDVKDMDGMYEISMDLPGF
SKENINAELKDGYLTVNATTSVNQDDSSEGRYIRRERYCGSMSRSFYVGDAVKKEDIKAR
FENGILALTIPKVPEQPKVETPNYIAIEG
>WP_160581716.1
MIISASRRTDLPACYPDWLFQRLKEEYVLVRNPMNAHQISRIDLSPKVVDGIVLWTKNPL
PLFRHLNELEKYSYYVQFTLTPYGPEAEPGLPSKNRVMIPAFCRLSREIGRERVVWRYDP
IFLSNVYTMEYHTKYFRVLASRLGEYTEKCTVSFLDLYQSTARNGRPLGIHTETGEQQLE
LMERFAEIAEEWGITIDTCAEQGDFGRFHVGRASCIDKDRLERIRGCQLKVKKDPNQRPE
CGCAASIDIGTYDTCRIGCLYCYANHHRDTVFKNSQRHNPASPLLFGEIGENDRIMERRM
ESCMDGQMSFHDWHG
>WP_135902375.1
MRSPEYAPLFRSTVGFDRLFDMLENSVRTDWPPYDIEKKGENEYRITMAVAGFSQEDVEL
TQHGPELTVTGQKSTAENGVQFLHRGLASRNFKQVFRLADHVKVANATLENGLLSIELVR
EIPEELKPRRISITSTATADPQPQISQDVKPGRKVA
>WP_135898580.1
MRHVDFSPLYRSTVGFDRLFTMLDSLAQPDGAQTYPPYNIERTGEDSYRISMAVAGFSDD
EISIEAHRNVLTVKGERKDEGTGEGSELLYRGIASRAFERRFQLADHVDVVGAALKNGLL
FVDLKRNIPEELKPRKIAINSAPAKAKQIEAKTAA
>WP_135873972.1
MPAQTDAAADWSGWRLAHAMAARFVRWAVDTEGSSRSSALIRIGLAMLFWSRWAGELLLY
MDQSPAGLFLAANFFVATTLLFVGYQSRVAAVWTGAVGLAMYHYFGFQLGREPWTHHHTY
LLAVSALLIALTPCGASYSLDRYLAVMRAERMGLPPPAERGNLLGLRLIVVQLSVLYFFA
AFDKSGYAFSSGARIEAIFLWYYAGSDYPAIPGLAWLATIVSLAVVALEYSLAFGLPFRA
TRRYLLLPGLAFHAIIYVTLPVYTFSATMVLLYLAYFDADAVDRVIARLQGIGPTAGEET
S
>WP_120424976.1
MTLKIKDPGSALTHFIGMLLALFAATPLLIKAARSPEQTHVLALTIFIISMILLYAASTT
YHTLDISPKVNQILRKVDHMMIFILIAGTYTPVCMLVLGDRTGWALLGLVWGIALAGITI
NALWITCPKWFSSLIYIAMGWVCVLAFGKITAALPKSAFGWLLAGGIIYTIGGIIYALKL
PLFNSRYKHFGSHEIFHLFVMGGSLCHYIMMYAFVA
>WP_135901799.1
MDDVPGNAARYRTGLAVRRSVLGDPHVDRAEIAATDFDQPFQELITEAAWGTVWARPGFS
KRERSIVTLALLAALGHDEEVAMHVRATANTGASRSDICEAFLHVAIYAGVPAANRAFKI

AKEVFSEMDGGKAVHAR
>WP_135901798.1
MPFVTLGGITLHHRYVEANGKTPAVVFINSLGTDFRIWDQLLSELDGEMPLLVYDKRGHG
LSDIGDIRSIDDHVDDLIGLIDHFGLDRLVLCGLSVGGMIAQGLYARRPEIVAAMILCDT
AHKIGTAESWNARIATVQANGIRAVADAVLKVWFTPPFHSERQPELDGYWNMLTRQALPG
YIGTCMAVRDADFTETARRIAVPTLCVVGDQDGSTPPDLVRSLADLIPGARFEIISDAGH
IPCVEHPAALVALIRDFVASLPSGETHG
>WP_135895629.1
MQRSLADSRVKFRHLQCFLAVAQFGSVQRAAGSLSITQPAVSKTVAELEDILGVKLFERG
RHGAVPTREGQLFMPHASACVSALRQGVDLLARAEGAAAATLEVGVLPTVAGALIPPVLK
RFASLWPRVIVRLATGANPELLERLKAGTIEFAIGRLADPERMVGLSFEQLFSEPLVAVV
RAGHPLDVTSGLPPAALQDFPVVLPPFGTLIRQSADSLLSAWGVLPLSAFVEVLSVSTGR
ALTLENDAVWFVPLSAVEYELTHGMFVRLPLPFAGTDEPVGLIRRSDTQPSPVGRAFIDA
VREVAQARMAASGGKAAGKVARKRGRGRTPAS
>WP_135891186.1
MPYAAVNGTELHYRIDGGRHGNAPWVILSNSLGSDLSMWTPQVAALSKHFRVLRYDTRGH
GHSEAPKGPYTIEHLAGDVLGLMDTLKIARAHFCGVSMGGLTGVALAARHASRFERVVLA
NTAARIGSPEVWVPRAARARTEGMLALADAVLPRWFTADYIEREPVVLAMVRDVFVHTDK
EGYALNGEAIDATDLRPETHGIKLPVLVISGTHDVAATPAQGRELAQAIPGARYVELDAS
HISNIEKADAFTKTVIDFLTESK
>WP_135872108.1
MAESRIRFRHLQAFLEVARQGSVARAADFLHVSAPAVTKTLRELEEALGVPVVERDGRGI
RVTRLGEIFLGHAGSAISALKRGVDSVRQDGALNRDPIRIGALPTVSARVMPLAMTLFLE
ENTGAALKIVTGENAVLLEQLRVGALDLVVGRLAAPEHMTGFFFEHLYSEQVLFVVRAGH
PLAEPGTDIFARLDEFPVLMPTRESVIRPFVDRLFITNGMTAPATEIETVSDSFGRSFMR
QSNAVWIISAGVVANEIASGAFVALPVDTEETKGPVGLTMRTDTAPSPAFTILLKTIREA
ARHHA
>WP_135855689.1
MHFLRCGETVIHYRVKGLDSGKPVIAFINSLGTDFRIWDAVTEVLGDDYAYVLHDKRGHG
LSDIGRPPYSIDDHAGDLIALLDHLGVKSAVIWGLSVGGLIAQGLYARRPDLVRALVLSN
TAHKIGTADMWNARIDKISADGLGSLVDPVMERWFTPAFRTPDNAAYAGARNMLAQQPEA
GYSGTCAAIRDADFTAAAGRIAVPTLCVVGDQDGSTPPELVKSLADLIPASRFVTIAGCG
HIPCLEQPLAYAQAACIFLKTLPEN
>WP_120446043.1
MAKGSAEKRRNMKKLLEEMEKNQELKAKIKELDENPKSTTKDYIQVAAENGLELTEADFQ
PAGSVGELADDELEAVAGGKDACTCVVGGGGQAYDEKTCVCVLGGGGEFTDGEARCICVA
IGTGQHG
>WP_120446041.1
MKRLLEEMEKNQELKAKIEELDKNPESTPKDYIRVAAEYGIEIKEEDFKPAQGELTDDEL
DAVAGGEPCACVFGGGGTANQSDDTCACVFGGGGEYSDGSCRCACVGGGAGDGHHYVPVS
DLFR
>WP_169251451.1
MDLTASVLAAPAAPADDARVLVVLPSLGTSAAALWQQAATELTALSPETTVIGIDLPGHG
RSAPIDVPAGTSVAPRITMSDLAEAVLATLDRVLPDVAAPGAPVDLAGDSIGGATALQLA
LDHGDRFGRIAVFCTGAKIGEATAWEERAQTVATSGTPTQVIGSAQRWFGEGFMDREPDA
SAALLHSLQDADRFSYAALCYALADFDVRARLPEITRPLLAVAGSQDQPTPATKLAEIAG
DVPGALLEVIDGAAHLVPAEAPVVTAGLLADFLQGKGLGAGAGGTGGAGATAASDSPADL
PTASGPREASRDEVREAGMTVRRQVLSDAHVDRANAKVDDFTSDFQDLITRYAWGEIWTR
PGLERRMRSAITLTAMIAGGHEAELAMHVKAALRNGLTRDEIKEVLLQSAIYCSVPSANT
AFSVASRALAEYEAEENAD
>WP_007228512.1
MKRTLLLLLAISIAACATGTDPAVTASAIDDFQPDARPTAKTMVYECDDAEFITRVGPGE
MALWFEDRYLILSQVRAASGTKYQEGDVVFWSKGDQVIFSAAGVRYANCQINHVRAPWED
ARRRGVDFRAVGNEPGWHLEVRGDQHLLFVGDYGATKIMFSNVHTTEDREQLHYLSQDDD
NSINVTVIESACIDTMKGDQFPYNVQVQLNDRNYQGCGRTLDHPWE
>WP_086005900.1

MSARLARFVKDPKHRLELLQDIHAASAQADLPPELVLSLIEVESHFDRFAISRVGAQGMM
QVMPFWKNEIGRPDDNLTLNKTNFAYGCRILQFYLQREKGDLHKGLARYNGSVGRRVYSD
KVYRAWNDHWRTEPLDWGD
>WP_040812715.1
MKQRVVLIEDELALAASYQDFLQRAGYDVTIYRTAESAVKGVEDCHPDLILLDIGLGHDP
EAGFELCRTLRARDALVPIVFLTARDEEVDVISGLRLGADDYLTKDISRSHLLARISGLL
RRVVALRNPENQEQVLKRGDLELNSERLTTTWGKSQIALTYTEFWMLYVMAKNPGHIKSR
EQLMEAARVVLDDSTVTSHIRRIRRKFETADKQFKHLETAYGLGYRWRGDA
>WP_007226915.1
MKNIQRSIGKSITALIMVLLGSSMIYQAGALMLEDTVDGKDNLYSSEWGHWFTMPGDGAL
AAYAPQSTAASAIVDSSNNAYDFSSWDYLDIVVTGSVTDAGSYETDAGGCTDPTASCWFG
DGQFRYQDVYSVIGIWSSSADEISWLDTIYDNWVDAVFTVGSDASIEVPEIEGAYLFLAE
NDGFFADNSGFYTATITTSVPEPASALLMLTALLGLFAIRRQRRAL
>WP_078486412.1
MRINIKLAAGFPLSRLESTFHGIETTRDENNTHTVRLTKSEVTADRDFELTWSPVPGNEP
HAALFSEQWAGDNYSLMMVIPPHQEGQGGALPREMVFVVDTSGSMHGASMGQAKAALKMA
LSRLAPDDRFNIIQFNSSTQALFGRAVGASPRNLARAEDYVDSLTASGGTEMLPALRRAL
TGEKELDRLRQVVFMTDGSVGNEAQLFEVIEQKLGASRLFTVGIGSAPNSFFMTRAARLG
RGSFTYIGKVSEVRSKMKALFNKLESPVLADVEIDWGEDVQVDMWPRRIPDLYMGEPLVL
AVKGEVDGKTVVIRGRSGDKPWQQRVTLHGGGSRGGIRLLWARKKIADLMDQKARGRTED
EVRHEVLVVALGHKLVSKYTSLVAVDKTPSRPLDQALIGKNVPVQLPKGWSAEKVFGSMP
QTATPALLNLLLGVLAMIGSWMVSVFGKRNRKNVADEVRHLNGEMYR
>WP_007229091.1
MQQHIAIVEDEAAIAANYRDHLQRQGFRVSLFADRDSAADAFAIQLPDLAIIDVGLGKEM
EGGFELCRDLRARAPGIPIVFLTARDSELDIISGFRLGADDYLTKGISQAQLTARINALF
RRVKALQKPEQEKHLVVQGALELNKERMTANWRGLPLELSVTEFWMVHTLALHPGHVKNR
QQLMDCANVVLDDNTITSHIKRIRRKFQALDADFGSIDTAYGVGYRWKG
>WP_082785960.1
ANIEVRLAKGVDKSTIASPYHQIKLDEPHHGIINVSLTNSVVANRDFVLQWRAKQGMSPM
ALVFNQQGKTHGDGASEDNVSENRQSDDHYSLVMVLPPKTDEHALSTLPRELILVIDTSG
SMAGDSIVQAKSALLYALNGLKAEDSFNIIEFNSELTQLSPTSLPANQTHLARARQFIHR
LQADGGTEMALALNAALPRGINRLSESSQSLRQVIFMTDGSVGNEQALFDLIRYQIGESR
LFTVGIGSAPNSHFMQRAAELGRGTFTYIGNVDEVEQKISLLLSKIQYPVLTDINVRFDD
GGVPDYWPSPIPDLYRGEPVVVSLKRSEREPQELVISGRQGHKNWQQSLSLKDSHGGAIT
EPDAGLDLLWARKQIAALELSKNGANDDKVKQQVTALSMNYHLVSPYTSLVAVDLTPIDS
SAMTRDAVVRQHLPLGWKPFGVLPQTATSSRFDMLLGAVTLILALLLAGSMLRQRRKERA
VILAIPYKQTL
>WP_007229132.1
MPDLMARALLPLLILLALQQLGSAGLIKAKAGLAPLMLAKAWEQSLASQGRPVKPWPWAD
TWPVAKLQVPSMGISQFVLAGDTGNALAFGPGHNLASAALGAAGPAMIGGHRDTHFQFLQ
HLRKGQRIVLQLPDGLLRHYRVKQMTVDTASGDMLWPNVGEQLLLVTCYPFDAFVTGGSE
RFVVTAEPESLPLQTLDALGGEPQRILL
>WP_007230524.1
MEDNIFQLQYALDTFYFLICGALVMWMAAGFAMLEAGLVRAKNTTEILLKNVALYAVSCT
MYMICGYMIMYGGDLFLSSITGDGVAGAEEAATYAPSADFFFQVVFVATAMSIVSGAVAE
RMKLWAFLAFAVVMTGFIYPMEGSWTWGGNAVFGMYTLGDLGFSDFAGSGIVHMAGAAAA
LAGVILLGARKGKYGPQGQINAIPGANLPLATLGTFILWMGWFGFNGGSVLATASVESAN
SVAVVFMNTNAAAAGGLIAALLVAKIMFGKADLTMALNGALAGLVAITAEPSTPTALQST
LFGGIGGALVVFSIVTLDKLKIDDPVGAISVHGVVGLLGLLLVPLTNDGSSFSGQLIGAV
TIFGWVFVTSLIVWGVLKAVMGIRVSEEEEYEGVDLAECGMEAYPEFTTK
>WP_007224114.1
MENEIFQLQYALDTFYFLICGALVMWMAAGFSMLEAGLVRSKNTTEILTKNVALYSISCI
MYMVVGYSIMYGGGDLTFFLDGIVGDGVTGAEEPATYAPSADFFFQVVFVATAMSIVSGA
VAERMKLWAFLAFAVVMTGVIYPMEGAWTWGGEAVFGMYTLGDLGFSDFAGSGIVHLAGA
SAALAGVIMLGARKGKYGPQGQTNAIPGANLPLATLGTFILWMGWFGFNGGSVLATASVE
SANSVAVVFMNTNAAAAGGLVAALIVARVLFGKADLTMALNGALAGLVAITAEPSTPTAL

QATLFGAFGGVLVVFSILSLDKLKIDDPVGAISVHGVVGLLGLLLVPITNGENSSFSGQL
IGAATIFVWVFGTSLIVWGVIKALVGIRVTEEEEYEGVDLSECGMEAYPEFITSK
>WP_007225392.1
MTSWDIAAIAAVLLVGVPHGGFDGAVARRLGWSKGIGGWLGFHLGYLALAAGVVWLWVQW
PVVSLAIFLAISALHFGHSDIADVPAPTTGSPSNRWLPLIAHGGLVSIAIPSLQPLAVQP
IFALLVGDEGAVMLLQAIRTLFLPWLLSFAGYAIYAVINPVWRKSLNSLIILLIVVFLMP
PLISFALYFCLWHSRGHTLRTWHRISAGSERRRSAIEAIIYSVMAWTAALVFFLYAEASL
SASLLQLTFIGLAALTVPHMLLVDLADKLNPQRPLP
>WP_140969888.1
MLGKLGELNEGYNVLTEMNGQCSDMLMDIGIYKMSNGKEELLFDNKNETAVLLLEGTIRL
EWEGMEQVIQRQSVFEENPWCLHVSKNVKVTITALSDSEVLVQKTDNDQEFASKLYTPNE
CQSVVAGDGVWEGTAQRVIRTIFDYNNAAYSNMVVGEVISYPGRWSSYPPHHHDQPEVYY
YRFNKPQGFGCAMVGEDAYRVVHNSFITIPGELDHPQATAPGYAMYFCWMIRHLENNPWN
DRIMEEDHKWLLEPNAKIWPEKE
>WP_140969579.1
MTEKMTRMTQFVKEEIANAITHGIGAILSIPALIILIIHASKHGTASAVVAFTVYGVSMF
LLYLFSTLLHSIHHPKVEKLFTILDHSAIYLLIAGTYTPFLLITLRGTLGWTLLAIIWTL
AIGGIVFKIFFVRRFIKASTLCYIIMGWLIIVAIKPLYENLTGHGFSLLLAGGILYSVGA
IFFLWEKLPFNHAIWHLFVLGGSTMMFFCVLFYVLPTA
>WP_040822867.1
MDKEMKILIVDDFSTMRRIIKNLLRDLGFTNMAEADDGSTALPMLRNGDFDFVVTDWNMP
GMSGFDLLKAVRADEKLKTLPVLMVTAEAKRDQIIAAAQAGVNGYIVKPFTAAVLKEKID
RIFERVGN
>WP_007224228.1
MARVLIVDDSPTETHRMTKILDKHGYEVITADSGEDGVAKAKETLPDVVLMDIVMPGLNG
FQATRQLSKNASTSHIPVIIVTTKDQETDRLWGQRQGAKGYLTKPIEDSALLNTISDVLG
>WP_007224227.1
MEGNFENLKVMVIDDSKTIRRTAETLLKKVGCEVITATDGFDALAKIADTKPNIIFVDIM
MPRLDGYQTCALIKNNSEFKQIPVIMLSSKDGLFDKAKGRIVGSDEYLTKPFSKNELIGA
IEAHVG
>WP_040541578.1
MALEYILPGTVSQESPAVMHTLEFKEEPGRVLLVLPTWVGDVVMATPFVRALFMRFPDAE
ITLLMNHHLYPLLEGSPWVQHCEFWAPRKKTAEAKQQQRELLNRLKARRFDLAVMLPNSL
RSAWLCFRAGAKRRVGFSRDGRGLLLTDKVEVPNRVAGGYQPLPLCDYFAVLGDALGMEH
PGDRLALFLTDQANDAVQSRLLKDGVLPEQPLVVLCPGANFGASKCWDPKRFAAVADRLV
NRHNAAIAISPGPGEEPLAEAIRDNMDAPSFLLTQPCLTLGELKSLIVRADLLLGNDTGP
RHFGRALDTPRVTVFGPTEQRWTETSHGDETIVNVDVPCGPCHKKVCPLDEQVCMTQVTV
EMVSEACEEQLSASC
>WP_169253931.1
MIAASALVLSACGSGEGGESGSDYLACMVSDSGGWDDQSFNQSGREGMENAKKNLGIEEK
LAESQGDADFGPNVDNMVQQGCNLTFGVGFLLEDTIQEAAEANPDLNFALIDSTFSDADG
KPVTIDNAKAVVFNTAEAAYLAGYVAAATSESGKVGTFGGIQIPSVTIFMDGFADGVDKF
NEDNKKDVKLLGWNKEKQDGSFSGDFENQGQGQELTKQLISQGADVIMPVAGPVGLGAAA
AAKEAGDVNLVWVDSDGYESTEYGDIILTSVVKQISQAVEDTIKEGTEDNFSNEPYVGTL
ENEGVGLAPYHDFEDKVPEDVKKDVEELKKQIIDGSLVVESESTPK
>WP_169253893.1
MNMLAMRDHSSDELRKKLLKRDLMPEAIDVLIEKLKNSRLLNDEEFAHRFARAQRENRKL
SRSVLKRELSKKGISPELASEAVADIDGEEELAREVAEKKAASTRRLDYAVRERRILGML
ARRGFPSAICIKVTRDVLTDD
>WP_169253863.1
MIPLALAALLIFFLFNSRRKQKARAEQIKSGLVPGATVMTTFGVFGTVLSIDEENNQVTI
ESGPGTVLRVHRQAIGQIENNQAAAPVDAPGADAPAADVDAAADDEKPAITDAELDAMNE
RKRAEKDTADDETAEDISADESAAKTEDADAVAEAEDVDIDAAETDADSAAADDDSTDST
DSDSDKKN
>WP_169253834.1
MGLDDHIFNRLLKERIIWLGSEVRDDNANAICAQMMLLAAEDPDADISLYINSPGGSVTA

GMAIYDTMQYIKPDVSTVAMGMAASMGQFLLSSGTPGKRYATPHARILMHQPLGGIGGTA
TDIKIQAELILHMKKQMAELTAQQTGKSLEQILKDNDRDHWFTAEEALEYGFIDKMVTRA
SDVENN
>WP_169252912.1
MKHLITGRRVTWAAIGMVGALALSGCGGAGGSSAGGGGDEVNVLMVNNPQMQDLQKLTAD
HFTKDTGIKVNYTVLPENEVRAKIGQEFAAQAGNYDVASLSNYEIPTYANNKWLTPMDEG
VADAEGFDQDDILAPMAESMTVDDQIYGEPFYGEGSFLMYRTDIFDKAGVEMPEKPTWDE
VAKLAAKVDGAEKGTKGICLRGLPGWGEMFAPLTTVVNTFGGTWFDEDWNAQVDSKEFKE
ATNFYVDLIKDHGESGAPQAGYTECLTNLQQGKVAMWYDSTAATGTLEADDSPVKGKIGY
VAAPVKETDSSSWLYTWAWGIQAAGKNQDAAKQFVAWASSKDYEETVAEELGWQHVPAGK
RTSTYENPEYQKAAEPFYQQTEDAINSADPESPGVQPRPTLGVQFVTIPEFADLATGISE
DVSSAIAGRTTADKALEKGQKEAEKVGDKYKK
>WP_169252886.1
MSLLGEPLSILALGLFNGFALCVFVLSLPPLRRPTLSSRIAPYLRDQESLVDIYAPPTPR
ADGFWGLAKSWLVSSTLWVTSRITTDATLRLRIDRLGGNATIERFRISQVLSILVGMIVA
GGLAGALSAQRGFSPIVTVVLIISGGVAGHVFNDWRLSQAIARHESRVLAEFPTVAELLA
LSITAGEGIVEALERVCRTCSGDLIDELRAALAATRTGTPLVEALDTMATRIAIPEIVQF
VDGLAVSMARGTPLAEVLRSQAADVREQSRRRLLELSGRKEIGMLVPVVVFVLPVTVIFA
VFPSLTVLDLSP
>WP_169252885.1
MSYSQFALLSGACLGAGLFLIWTSLWQRPQSKRTQSRWVRELDDMLTGAGFPRLRPAHLL
LISVAAFCIVTVVSTVLTGSWAIALCFGLFASWLPHRALQHRARSRQVMRRELWPETLDH
LNSGVRAGLSLPEALSSLAHRGPEPLRPLFEVFAEEYRASGSFALALERFRQVSADPVAD
RIVVALSVTRQVGGSDLGTMLRALAQFVRDDARTRNELSARQQWTVNGARLAVAAPWVVL
AFLSTRPETAVAYNSRTGLILLAAGFVVSLLAYQAMKRIGRLPAEPRVIEGSSLSAAHRR
FADNAGMSSGFTADSDDSADGRAA
>WP_169252597.1
MVTSAPRPPKLSLGDLVPGYLGDFGPEEFLEGMEFTDLDLAEADATQATFLDCRMTNVNF
GDAEAQIDLSGVRISGTEITDCRADTWTIPRGNLLHTDVSGTRIGAGVAYDSVWEKVRFT
NCRISYLNLRESKLTDVEFRDCKIDEIDLDRAKASRVAFPGSSVGVFQCEGATLGNVDIR
GLEPHKISGVHSLRGAIIDDTQLMLFAELFASELGISVE
>WP_169252584.1
MSTFTKVLDRWLTVTTAAAIIVMMLHTVTHALARSIFHAPIYGTNEIVEYWYLPIVALLG
IPAAQLQKEHITVTMAIERAKPATAALFTVFACILGALVSAAFAWFGLMKALENTAIGST
ADVSAVITWPVYYLVPIVFVLLVVLYILDAVAILRRRRTETGEEQ
>WP_169252403.1
MNWKSLVNRAARIGVREGLRYLRQSQSKKNSKGASQPTDARRPDTARSGGGGAASAGTAD
RSASSGQGGSGSYPGDYTGSITVSYSPDLDGDADPGEVVWGWVPFEEDHTQGKDRPSLIV
GRDGRWVLALMLTSKDHIPGGVGEVRQDRHATWMNIGTGDWDSQGRPSELRLDRIIRLDP
DSIRREGAIMPRDVFDRVAEHISG
>WP_169252333.1
MDILSSTLAAGGNAAAASESPIPVWFMITSGIIVVAILVFDLLLVVKRPHTPSMREASIW
VAFYVALALVFAGALFAIGDAQHGSEFLTGWLLEYSLSIDNLFVFIIIMGSFSVPRKYQQ
EVLMVGIIIAIVFRGIFILAGAAIISAFVEVFFIFGIFLLIVAYRQAFSSEDEGDGENGL
IRFLRKRINVVDEYHGNKLRVTLDDNKKYWTPMFIVFIAIGSTDVMFALDSIPAIFGVTQ
NAFLVFTANVFALMGLRQLYFLLGGLVDKLVYLHYGIAAILGFIGVKLIIHALHSSDWEF
LSWGHSIPEVPTWLSLTFIVFAMVVATLASLMKMKKDGISFRDMSSEESEGA
>WP_169252137.1
MNFMPGSTASNMPQSRYVMPQFEERTPYGFKRQDPYTKLFDDRVVFLGAQVDDTSADDVM
AQLLVLESQDPDRDITLYINSPGGSFTAMTAIYDTMQYVKPEIQTVCLGQAASAAAVLLA
AGTPGKRLALPNARVLIHQPAMQGQGQGQASDLEIQAAEVLRMREWLEATLAKHSNKEAT
QISNDIERDLFLTAAQAKDYGLVDQVLSSRKDAN
>WP_169251947.1
MNASLTDSSPAVPDGSGPVGSAGADDLPPADDHRIARRRSALRAELTKLAGQVKPKLRGW
FHAGAFPLSMIGGLALVIISPTIESRIAAAIFAVTGMLLFGTSAVYHRGRWRTRARLVLR
RLDHANIFLITAGTYTPLAVLMLTTDQAILLLSVLWGAAALGVAFRTIFTTAPRWLFVPI

YVGFGVAGVGYIPQIWATNFAVGLLVVLGGVCYIAGAVIYGIKRPNPSPKWLGFHEIFHI
LTILGYGCHLAALIIAAVAAY
>WP_169251583.1
MIVLTAALIAAGIVLAAPSDPGARLRELLHRAEDGESVPKGVRAVFRRGGHTADDQAERL
WAIAAVENCAHLLKVGMTPQAVMTTLSRHNDALAPISRAISLGEEPGRAIATRSSALSEA
AAHVLTGMAAVWTVSERSGAPAAEMILRYAAAQRDALDAERERRIAMAGPRSTVRVLSWL
PLIGVGLGLLIGVRPLELISGLPGQLSIGAGLLLYGAGRWWMRTMMLRAQR
>WP_169251255.1
MNGKQARTGRGRIAWTAAALALLAIAAVLITAGLLTDHSPPQPDADSAAEQSESRTTPSA
DAQPTDPSTPRTEQSSDGDQQAGMEASAPTRLRIPAIDVDTSLMDLGLTADDELEVPPLG
KDAPAGWYKRSPTPGEVGPSLIVGHVDSASEGPAVFYRLGALEPGDTVSVTREDGSEAEF
TIDDVTDYGKDSFPDYRVYGNTEDPEIRLITCGGEFDDDTGHYEDNIVVTGHLSDGG
>WP_169251032.1
MNIIDKYLSKVENVLAGGCLIAATALAVFAVLLRNITGDVLFWSEEAVIYLIIFSTFFGA
VVALRHNDHVAVDIMPTLLKGKAKKFFVVLGGLATLVYAGFIAYLSWALITEPFSRTTIT
PALKLPLWVVELSLAVGMTLFFIRAAEMTVRALRTPAAELDKDVLAEEAAAVGIAVEDIA
IVEDGRDRANRADGNDGGDRLGDGETDEKGDDR
>WP_169251018.1
MTRTLRRPRTLAAAAGIAALTLLATACGGGGGGGGGEEGEQSADFITIATGGSSGVYYQVG
ATMSEILADELGADTSVQSTGASVENLTLIQDGGAELAFTQGDAVDQALAGEGAFDGKQI
DSLPVVANLYDQYVQLVTIEGSGIDSIEDIKGKSVSVGDQNSGVELNARTVVDAYGLSYE
DFSADYLPYAEAIDQMRNGQLDAAFVTSGLPNSAVTDLATSDDVKVVPFTGDGREKLLSE
HDYFGEGEIPAGTYGDSKAAETLTIPNLLVVSPSLSDDAVYDITKTLFDSIDKIQSSHNA
AKDITVDNAQDVPVSELAPGAKQYFDEQG
>WP_169250942.1
MRVIRFFEDWVVIAAFMVIVIVTFVNVLSRYIFKASLAFSEEITINLLVVLTMMGAVVGI
RLGAHLGFTYLVENTKAGTRRALLFTGTGLIVVFLAVLLIWGGEMTIAQGVRGRATPSLG
IPQWLFTLSIPLAGLLGIIRSIQALRTALGEDTSAEAVVDRLAGEATPTVDSSEFSADVK
GGRK
>WP_169250805.1
MSTSHSADQQVGHSAGRSADDSAAAGDQPLPSRRELRRREAEAAAAQGEPTAVYSDAPPV
YEQPQPTHAQPTHAQTTQAMPAVPQGHGGQSTQGRLGEDQPQNRPVTRRRRRRQPAEKEP
LGPIRGTVRTFGELCITAGLVLILFVVWQLWWTDIQANRDNEVLADELTQDWANQDPNEL
PDDPDEPVVAEPVGKNEAFGIFYIPRFGDDYYRTVAEGVDLEPVLNRMGVGRYPNSAMPG
EVGNFSVAGHRVTYGKPFNQIANLRPGDEIIVQTKDGFYTYTFRNFDIILPDAVEVLSPV
PDAPDFKGKDRILTMTACNPMFSARERYVAYAELTDWTPAGNGAPDSIKDSKAYDKVSKN
GGA
>WP_169253925.1
MRARTTQIAALAAVGVLTLSACSVTAPEALEEERLGCLVSAPAGFDDHSAGALTLEETEL
ARGAGVFSGTSSQRVSGGSATSAALDRMHGHDCALTTVIGPGGADELADFAAAHPDDLFL
GVSPGTDDLPKNVLSMDFDLVPPAYIAGFTAATASETGKVGIVVSHGFPQADRILAAFDA
GVDLYNKEKDEDLPKAESYHPSSDRAASANGSPRAIDDTRDAGKDYFERSFDADVDVLVP
FGSAAAMGVVTSADEKRTDLTAATEDPESGDDGPSLPKVIWYGTAGGFSKAIVATVEPNV
RRGLRTMFPDWPQSRNPDEVAPAPKTEPEEIGGFEVSERRYEGTIDNGGVRIVAEDGFLS
RVSDAGRGITDLRERIKSGEIDPEKG
>WP_169252724.1
MRQVEPSVELLAKPDIDWEAMRTYLDEVGGTSWADRVEDAESPDAEDLLEFSGRMCYRSW
EPGLNPNVSRVRTDSAQYLGNVVKSQHGSVLEHANFTFVLHNVSRVLTHELIRHRAGSAF
SQESLRYVRLTDIPFWFPEWAREDPELMERSLQVLDTLEEHQKWMAEHFELDEEGTKFSH
KKHMTSFMRRFAPEGLATGIVYTANLRSLRHVIEMRTAKGAEEEIRLIFNKIGEVMREEA
PAVFADYEVVDGEWIPGTRKA
>WP_169252371.1
MSPITDEFSSDDGLTRAEVRAKKRRRMLRRRRTTTIVIICILVFGIGGFFGVRAAGGVFD
DLFGPKGDYEGQGTNEVSIEIAPGSSARTVANQLVEAGVIMNSEPFLDEIERREATIQAG
TWTMREKMSSEAAVEALINPIAPPKITVAEGKQVEEIKAIMIESGMNADEVDKAIDDKTP
KDYGLDIEAPSLEGYLYPATYDLNKQKTAEDIVQEMVDKTETELDELGIESKDANRILTL

ASLVEKESPGDPEVRSKVARTFLNRISEKSKTGGLLQSDATVAYIHGARSDLTTTKKERQ
SDSPYNTYKKKGLPPGPINSPSRGAVEAALEPADGDWQFFVATNPDTGETKFADNYEDHK
KNVEIYRKWLREHRKDNG
>WP_169252360.1
MRIVLGVAGGIAAYKAAHIIRRLRELDHSVKVVPTANALKFIGAPTLEALSGQTVTTDVF
DEIDTVNHVRIGQDAELVIIAPATADLIAKIAAGRADDLLTASVLTTTAEVVVAPAMHTE
MWLNPATVANIATLRSRGIHVLDPAVGRLTGPDSGPGRLPEPEEIVDFALSVKDGTADDS
ADALNRPSGALSGRRIVISAGGTREPLDPVRFLGNRSSGKQGIALAKAAHAAGASVELVA
ANIDTGLLSGLPADITVTPVESTLELQEAMHTAQARADAIIMAAAVADYRPAETADSKMK
KSGDDGLTLRLTQNPDILRGLVAERSGQTGLRRQIIVGFAAETGDSDTTALDYARAKFER
KGVDLLVFNDVSDDRAFGHDDTMVQIISSDRGDIVVGEEFHGSKDHVSQKVIAAVSDQIT
HVESTT
>WP_169252217.1
MFSSAEELVNFISDNDVKFVDVRFCDLPGVVQHFNLPAASYGTEEITEGLLFDGSSITGF
QGIHESDMKLLADVTSAYIDPFREAKTLVVTHSIVDPFTDEPYSRDPRQVAAKAEAYLQS
TGIADTAFYGAEAEFYLFDSIQYENTPGNSFYRIDSEEAAWNTGADEAGGNLGYKTPHKS
GYFPVSPQDHFADIRDEMSLTLEQIGFEMERAHHEVGTAGQQEINYKFNTLQHAADQLLD
FKYVIKNTAFANGKSATFMPKPMFDDNGSGMHCHQSLWKNGEPLFYDENGYGGLSDLARW
YIGGLIEHAGAVLAFTNPTINSYRRLVPGYEAPVNLVYSARNRSAAIRIPVTGSSPKAKR
LEFRVPDPSSNPYLAFSAQLMAGLDGIRNRIEPPEPIDKDLYELPPEEAKDIKLVPGTLD
EALIELEKDHDFLTAGDVFTPDLIETWIRIKRENELDVARLRPTPTEFELYYAL
>WP_169251582.1
MSEWLDASLVADVRKTLLDKPGPVTTAAVAEAVQRTGRVLGSSALLELVTRLSAQLSGAG
PLQSVLEVPGTTDVFVNGPREIFADTGGGPRLLDLSLGSEEEVRSLAVRLAALGGRRLDD
SSPYVDVRLPDGVRMNAIVPPISGDTTTISFRVPKRSGFLYSQLCDSGFVPDEISPLIAE
AVTSRANILISGGTGTGKTVLLGALLSLVETIQRIVIVEDSRELIVTHPHTVQLAARQAN
VEGGGEVTLTDLVRNALRMRPDRLVVGECRGAEVRDMLTALNTGHEGGCATIHANTAEAV
PSRVAALGALAQMSPDAVYSQFSTAIDLVIHLRRCGSERGITELAIPTRAGAEAVVMEPV
WGRPSPSTTGTVNRRALARFEAVLEQKSAQ
>WP_169251567.1
MSVNIGVVGATGQVGGVMLDLLADDPGFEIGSLRLFASARSAGKTIDFKGQPITIEDAAE
ADPSGLEIALFSAGGATSKAQAERFAAAGVTVVDNSSAWRSDPEVPLVVSEVNPDDLDEP
PKGIIANPNCTTMAAMPVLKALHDKAGLTRLIVATYQAVSGSGLSGVEELAGQLEAGLPD
ARKLATDGTAVNLPEPNNYVEPIAFNVLPMAGSVVDDGQNETDEEKKLRNESRKILGLPD
LLVAGTCVRVPVFTGHSLSIHAEFDSDISPEQAAEILSQAPGVSLDEVPTPLKAAGQNAS
FVGRIRADQSAPVGKGLVLFVSNDNLRKGAALNTVQIAALLAAKLEAKAA
>WP_157361095.1
MGDPVSGGGGWRHADRVVAVESVVSGTVECMARKTLTAAPRLEKLRLEAIAVGDADHLQA
HESYSGGRYVASDLRERELSGISFSECEFVELEASETDLRAATFVDTRFERLNAPIFTAP
RSSFRDVSFEGSRLGSAEFYEANWSSVHFVHCRIGYLNLRGARLEDVLFTDCLIDELDLG
AATANRVSFIDTQINNLDLTRSTLTNFDLRGVELRQLGGVEYLKGATLNSYQLSELAPLF
AHHLGIVLDE
>WP_148224521.1
MGNTTFSDGAIKGLAAALVVCLHFACASLASAEEDASSPGPREVVAAATDNIMALAREAP
AYFDTDPDRYTVAVGEELDRVVDFRGFARGVMGRFASKELYQGLDEAGRNQLREHLMKFT
EVLRSGMVNTYSRGLLAFGGSQVELGEVDMAPGSTRVASVTQRVFGDDGKIYTVKYQMGQ
YRDGRWKLRNLIIENINLGEIYRGQFEAAALEAGGDLNTVIANWDDNRVKSLSTEE
>WP_007228891.1
MYYGEKLNSISHLVGAVIALIALGALLALGIQTGDPLVIVGFTAFGLALVLLYTMSTLYH
SFQEPRLKKAFQLLDHISIYLLIAGSYTPFMLVSLGGSEGKMILSLVWTLAIIGILSEVF
LSGRVVKTIQLVIYLSMGWACSLEFASLKAALPEVGFFWLTAGGIAYTAGIVFYLLDKMN
RLDHSHGIWHFFVLVGSVCHLIAVIGYVR
>WP_040823262.1
MVDVALFPVPNSVNFPGVPCSLHVFEPRYRQMVRQCIDQNLLMGVCHTEKVLHRKEREQT
LEEALNSNQTTYKPRGIFSAGPVELLEELEDGRMLIQVNNEVRLQLGEEKQTLPFGIWAC
EELVDEALDETGELALNQSQSKILQRLLAVTHGNEDAQDMLNSIHWRSMPAQTFSFAVAG

LLGMPPETSQALLEMTSAQMRLDTVLEMINTMGTALS
>WP_009774169.1
MSIQHKLPRSSGSALTILMVLALAIAGCSVNSSPRSEASNASAIPPGAFSATVNYVHDGD
TLYLDTGREELKVRLIGIDTPELAGQQRPDAEECYGAEARALLRDFLPEGTQVWALEDRE
PEDRFGRSLLYVYLDDGTFVNLAMIELGAAEAIKVGLNDQYWPELRDAEDAAHSAQLGMW
GAC
>WP_009774019.1
MKRARSQLPEVASVTQRLAVLLNAGLTPSSAWFHVARGRSADGVAALVALAGEEPGGAAE
RIVRAAEGLAPLDRQAWNSLAAAWFVAGQVGAPLAAALRTHARALRMLVHVQREVATALA
APVATARMVLALPAIGLVFGALLGFDTIGVLVSTPIGWGCLVVGGALIAAAVRWNRRLVR
SATPTQAAPGLECELMAIAVSGGGSLVNARVVVAGALERFGLMGDGDHLEGVLRLSHEAG
VPAAELLRAEADELRLAADADARAGAAALSVRLMLPLGLCVLPAFMVLGVLPLMVAVISS
TVAGL
>WP_009771703.1
MSLILGIILAVGVTLVAAPFLWPAQGERRVRGRSAWSLRLRERLVQAGLPTTSPSIVLIV
SVVFSVAVAAVTFVLTSVIVVVLCSAALALVLPTLAISWRARARRTATQIVWPDVVDQLV
SAVRSGLALPDSLMTLSQTGPLVTRSAFAAFAARYRATGNFSIAVDELKVALADPVADRI
LETLRMSREVGGSELTNVLRSLSVYLRQEAAIRSEIEARQSWVMNAARLGLAAPWVVLFL
LTTRPEAAAAYNSASGVALIVAGLILSLVAYRIMAGIGRLPQQPRWFA
>WP_007234989.1
MTEIPLFPLSSALVPYGYMPLQIFEQRYLDMVAACMRTGTGFGVVWLREGSEISGGSHNT
PDVGKYGTHARITDFDQLPNGLLGITIRGEERFDIAEVWRDSSGLIRAKVSMEAPLAPAS
MTDEWRSLEIVLRGLESHPHIQRMNLTIDYNNAWEVAFTLIQLLPFDEAIKYELLGLSTL
DELIVELDILLNQISGEDG
>WP_007234840.1
MFVCICNGVTDSSIRREMEAGATSFADVQNRLGVARQCGSCEHLARAIVNEFSKPDPRYF
YNACSDESMAAVA
>WP_007234584.1
MTEDKEYSRMRWASRRGLLELDLLLEPFMEACFRALEPQLRDDYQQLMGHEDQDILNWIM
GREALADETLTAITEQIRVHNRSKLR
>WP_007230552.1
MNQTLLIAQVAAFIATSCTVLFIGFSIRDALKRREQLWIKIRASHVSKAEAGAEFVRTVL
LETVRPMSSVQKLLRQTQKNLLRAGVGRNAEDYVADGLWQGLLGGISIMLAVSLLSSPVS
GLLLGTAGGVLWAMWIKPSMLDSNATQRSRLIYRRIPYALDLSVLVLETGGTLREGLEEI
SLQNDPLAEELRITLLEMDSGSTQAAALRGMGQRVGLESLETILTAINRGEETGAPMVAT
LITQAEMFRERRLQEIEKMAVEAPTKMTFPNMMIMVSVLLLIIGPLLIQLVSSGLF
>WP_007230536.1
MGLIIAYALIAVSVTALVYAGLQSFVPVAVTRWRSDQEEIDDKLQNIFYTSSEARTFLIL
KYGGTLAAFFIGLWFMNSLVFGIFLGIVIYLLPEVLLDNILRRRRERLEAQTADVMTALS
ASIKSGMTIEQAFSEMVDNMYPPISEEFALIRERIDAGQPVIAAVKSADERLQVPRLSLI
FQTICISLERGGRLASLMDRLAESTREIERVEERVRTETAGLRLSARIMFLMPFFICGLL
YLIEPDQVMLLFDNLVGNIVLVIAIAMDISAYFIMKKLIELDI
>WP_007227341.1
MAPSAIQSLDALEQRFREDAVEAPVQDLERMEWVGTCLSIAGVPLLIGEGELEEIIETPN
VMAIPGTKPWVQGVGSHMGGLIPIISGDVFFRKRPYSGRVRDYCMVLRRPGFYFGITLSG
LERDMKFLVETRDMTVTTDPDFAEYTLGGFPDQDRVLAVLDIDKLIADSDLSNAAANDPD
SPEERTND
>WP_007226034.1
METIPLFPMHAVLFPHGRMFLQVFESRYLDLIGQCMKEDSGFGLVWLKQGGQEVYRSNELV
DPQLAQIGTYAKIVDWDSLPSGLLGVTIEGSDRFRLLTSYQRKDHVHMGEVEWIETAGAT
ELPENYAELWGLLQTLLDHPHVDRLKLNPVVNDVNAVSCLLAQLLPIEERVKFNLLAAAE
PLDRMARIMTLLDQYSE
>WP_007224867.1
MQKIRVYWRDIPSQVIIKRGRLRAKAPLTQRFQVAIDRAAMTAGRGSSEAYVADWRRETT
SISGDGDLGQLAESEALMLENQYDDDRLKQLVANRGLEAE
>WP_007235031.1

MKHSGILVVDDESVIAEELCEFLSSFDYTCQKALSVNEALALIETNLHITLILTDMRMPG
RDGAELIQALQEMPGRQFEYLMISGHLDADEDLKHINNEGVTLMRKPIDIDALLLFLEER
EFTAVPNEN
>WP_007234880.1
MQKEMDTSSLKVLVVDDEAFVLKLTVRILSKLGYDNVVTADNGVVALGEIDNVTTPFDVI
ICDLNMPEMDGIAFMRHAADRNVSAGMILLSGEDERMLETARDLAAAHKLHILGVIPKPL
KPDALSNLLNTFQPTAVVEKQGWHQEGISETELLDGMNSDQLHLVYQPKVNISTGEVTGV
ETLARWMHPEKGLLGPGAFIPLAEETGHIDQLTCAIYRKAMHQAGDWLAQGITMKISVNI
SVNSFTAPGFTDFLIETAQNEGMDLSNVVLEVTETQVMDNALGILETLMRLRMKRFALSI
DDFGTGNASMEQLKRIPFSELKIDRAFVFGAAENAGARAILESSVTLAKSLKMDIVAEGA
EGREDWDLLASLGVDTVQGFYCAKPMNNADLMSFLEDWTGPH
>WP_007235808.1
MVKHSNTEDPWALKVLAAVAELGPQRYYDPVITSEVVKNDLVSLIRASQKLKEKVAIASP
EARHDRRNIIGAIRGYSEMLLEDSEVLPAAVRAHLLQILAAAKNEPKPASESATPTKSVT
LLPSEEPGVILAVDDLPENRELVSRLLQKTGHTVISAESGEEALELLDTMGVDVVLLDLV
MPGIGGAEVLKRLKEDERLRATPVVMISGQQDMDQIVMCIEAGADDYLLKPFNPVLLQAR
ISAGIERKRWHDREELYREQLERREQFIRATFGRYLSDDIVDEILERPEGLELGGDLREV
TIMMSDIRGFTTLVEHLPPQQVVTLLNRYLGRMTEIILEFGGTIDEFLGDAVLAVFGAPR
RNDDDPDRAVRCALVMQEAMADINIANSADGLPEVEMAIALNTGSVVAGNIGSERRSKYG
FVGHAMNVTSRIEDVAKPGEILISQTTYEKLESDYKFGNSRSLSVKGIEAELRVHAVLGG
IQ
>WP_007225300.1
MDTAEQQLLLIDNDEVERKSVAAYLKGAGFIVLEASNVSQGLDILADHQPEVVLCDLNAT
GTDSGPLQAIKSDFADTPVIVMATDGVMSDVVWALRYGAADYLIKPIADMEVLEHAISRC
QEQRQLRQQNLDYRQKLEQANQGLQESFKVLELDQLAGREVQLKMLPPTVKQFGKYQFSH
RIIPSFYLSGDFIDYFTVGDDFVVYFIADVSGHGASSAFVTVLLKNMFARKRSDYLHQND
ASILSPAAMLDIANRNLLTTDIGKHATLCVGVIDLRTDTLSYSVAGHLPLPMLTVDGEPQ
YLQTEGMPVGLFEAAEYTEATISLPPSTVLTLFSDGILEAISVKGVLGQERFLLEQLSRG
PNSIDGVLEALKLNEIGEVPDDIAVLLITKDVHLGDSIIHADTGSDSE
>WP_050756508.1
MDLILASTSPYRRQLLERLQIPFRCESPNVDETAHPGESPAALAQRLAAAKALDIASTNP
GAFVIGSDQVASLSGSCIGKPGSHAAASKQLHDSAGQRVDFYTGLSLINLSIDYHETLIE
RFSVVFRELESLEIETYLQKEKPYDCAGSFKCEGLGIALFEKMIGDDPTTLVGLPLIATC
RLLKAAGAPVLEH
>WP_009772888.1
MRLYLASTSPARLATLRAAGVDPVLLASGVDEDAVAAAAPGPLDGPALVELLARAKAEAV
VGSRINNEPIDGFILGGDSAFELDGELFGKPHEPEIARRRWHAQRGRTGVLHSGHWLIDH
RGGQLRGATGAVSSASVTFASNITDAEIDAYVATGEPLKVAGSFTIDSLGAAFIERIEGD
PHAVVGLSVSLVRQLMRELDAEWTDLWNIERPTL
>WP_040811778.1
MTMKKILVVDDEPDLRDMLRFALETEGFEVLEAADTQKAYWLITDQDPDLVLLDWMLPGG
SGIELLSRLKKEEATQSLPVIMVTAKAREEDIIQGLDMGAHDYITKPFSLKELLARIRTI
FRHTEDDDVNHQLRVGDLVLELDNRRVTLGSQVLMLGPTEFKLLQFFMLHPERAHARKQI
LQHIWGNNACVDARTVDVSIRRLRKTLQSAHPVYSELIQTVRGTGYRFSPRDLVAA
>WP_007227708.1
MSEPTVLVVEDEKAIRDMLRMALEVAKYRFIEAENIRDAHVLIVDERPDIVLLDWMLPGG
SGLELLRRLKREDNTREIPVIMLTAKAAEDNVIQGLEVGADDYVTKPFAPRELIARIQAL
LRRSAKDSAQGRIGLNGLVLNSDSRRVFAGEIALNLGPTEFNLLQFFMSHPERAYSRSQL
LDQVWGANVYLEERTIDVHIRRLRKALQTDHADYGELIQTVRGIGYRFASREHS
>WP_009773595.1
MKFQVNRDVFSEAVSFAVKLLPQRTTLPILSGVLIEATDEGLTLSSFDYEVSARTQIKAE
VDEPGRVLVSGKLLAEIASRLPNAPVRFSTEDNKITVACGTGHFTLSSMPVEEYPTLPQI
SDQVGTLKADLFSAAIAQVAVAASRDDVTPVITGVQLEVSQNNISLVATDRYRVAVRDIE
WDAGASGVESATALVPAKTLVEVGKTFGNSGEISVAITSTDERELIAFHADNKTVTSLLI
KGNFPPVRRLFPETVDNFAVMNTAELIEAVRRVSLVLEREAALRFTFTTEGVTLEAIGSE
QAQASETIDAFLTGDDTVVSLKPQFLIDGLSSVHSEFVRISFTKTENPNKPGPVLITSQS

SKDQPGSDNYKYLLQPNLLLR
>WP_007225086.1
MVETLLIPLMLIVLAVFFLLSPVMMNRSLQRSSRSGVNIEFFKSRLSELESDRARGILDD
DEFEQLKIELERRLLDEADSGHTAPSAHVKTSFKTAIMLALLIPIVAVVVYQQTGAKADW
DIAQTLKNMRLKTADGEAAETDVKQLIRQVEERLEQRPDNGSYLMLLANQQMGLRNYPAA
AAAYQRLRTIYPDDASVLAQYAQAMYLSSDRTLTTKVTDMAELALRQDPQQPTVLSMLGM
AHFEQGDYQRAIDYWQRLLPSLGPVSPNRKIIMAGIEQAKSRLGSSDTTSIDRPDVIKNA
SIQLSVSIDEGIIASSDSVVFVFARSASGPRMPLAVAKLTVADLPVILTLDDSMAMAPGL
NLSSQKEIEVVARIAKNGIANPGPGDIEGRVGPIKLEEVDGVVAIAINKTL
>WP_007225370.1
MSYVINFEAVPEELKTLPQWVCWKAVVRPNGKITKVPMNPLTGIKASSINSKTWASFDKA
ATGMNRHGYDGIGFVFVRGDGLVGVDLDNCMRSHGQLETWAQDIVDRLDSYTEVSPSGNG
VHIICYSGASGLSYNKDGREMYSEGRYFTVTGNEYYVRGHSNEN
>WP_007229947.1
MTLLNTKIHLTDNQMHQNQDILGNISTAVVSLDSELRVVSLNSSGQDLLEASEARSLGQP
MHKLVANPEALMEVLRQVRADRSPLARRGMPLMLLSGREIHADLMLTPVSNSEHGINILL
ELQPVDRLLKISREESLHHAQETTREMIRGLAHEIKNPLGGVRGAAQLLARELSSAELEE
YTNIIIREADRLRDLVDRLLGPNQQMDSQCMSIHEVLEHICNLVRAETDNRVELVRDYDP
SLPDIIGDRSQLVQAVLNIVRNALQAAPSEEDCVITLRTRPQRQFTIGNQLHRLLCRLDI
EDNGSGIPVDMLHSVFMPMVTGRAEGTGLGLTIAQSIITRHGGMLECSSEPGHTRFSIYL
PMDLNHA
>WP_040821591.1
MSTANTVWIVDDDRSIRWVLEKALNQAGITTQTFDSGETILNSLRQNTPDAIISDIRMPG
MDGLELLGKINETHPDLPVIITTAHSDLDSAVASYQGGAFEYLPKPFDIDDAVAMTERAL
LHANEKTADSTEPEENSSSTEIIGEAPAMQEVFRAIGRLSHSNITVLINGESGTGKELVA
HALHKHSPRSSQRFIALNMAAIPRELMESELFGHEKGAFTGATSLRPGRFEQADGGTLFL
DEIGDMPSETQTRLLRVLADGEFYRVGGHVSIKVDVRIIAATHQNLESLVKEGRFREDLF
HRLNVIRIHIPSLRNRREDLPKLMQHFLLKASEELNTETKLLTPESEHYLSKLDWPGNVR
QLENTCRWITVMAAGREIHLEDLPPELLDQTIADGGTDDGNWQDNLRRWADQELSLGKSN
ILDTAIPIFEKLMIDTALKHTHGRKRDAAVLLGWGRNTLTRKMNELGMNTTIIDSTES
>WP_007229945.1
MHSQAKVWVVDDDSSIRWVLERALKQAGINNESFSDADQLLKRIVSETPDVIISDIRMPG
TDGLELLSQINASHPELPVIITTAHSDLDSAVASYQKGAFEYLPKPFDLDEVVAITERAL
AQVRERSIEAPVLEELPETEIIGEAPAMQEVFRAIGRLAHSNITVLINGESGTGKELVAH
ALHRHSPRAASSFIALNMAAIPRDLMESELFGHEKGAFTGANAKRAGRFEQADGGTLFLD
EIGDMPAETQTRLLRVLADSEFYRVGGHTPVKVDVRIIAATHQDLEELVRRGDFREDLFH
RLNVIRIHIPKLSERREDIPRLMQHFFQSAAEELGGEAKILLPATERFLSNLDWPGNVRQ
LENTCRWITVMASGREVHITDLPPELSRDVVPDPQSTDSDWRAMLQNWANNELGQGKQQI
LEQATPAFERVMIEVALKHTQGRKRDAAELLGWGRNTLTRKMKDLEM
>WP_148224536.1
MPKLLIPIVVTLLGTVVLTIGLSFGALEQRKTQLAVSAQDMVSINATLSPLLHFRNQSAI
REALLLKLRLETKPQKIWLRVLHVDGEILAKAPRRDSDRGTTQFIRTFRNRLEVLTAVDL
FAHNSEAHYRGALAAIPFMDAQFKMSTPVFSLIDPLRTDVPRSAYQQTLLQKADQPLPFV
AGYIEQGIFLGDILETIIPTLWQALIMSMIISATMLLAFYYFAVRSRVSISQRTAQTERS
DQKNLPQKMESARSVPTNDETTSAPEDRLTEPSLDETRSNIGDPATLDPVTSLPDRHQLL
EHMAQGMRVAAAEHRCMGLVLIEVCSIRDILRTRGREVSDNVLREMTSRILNSIRRSDFA
SRGYDALGEAILDADQFCIVLCDLDNIQGVGSAAERLLGQLRLPVTVADEALCLNVVASA
ATAPQHSKTPEGLIIAAKSALIQARESRAPNTILFSS
>WP_148224448.1
MFAPTNLDLRGRTPPGYIAVEGPIGVGKTTLARRLAEAFNYQVLLEDAHENPFLDRFYQN
RKEAALATQLFFLFQRSQKIADLRQTDIFEPVRVSDFLIDKDPLFARINLDPDEYSLYEK
VFQQLTIDAPLPDLVIYLQASPDRLLERILSRGVSSERGIDREYLEQINEVYSEFFLYYD
AAPLLIVNANEIDLSQGDEDFSQLVNYLLDIRSGRHYFNPTFFG
>WP_148224378.1
MALLTVLVVILRYGFGVGAIAAQESVIYLHGALFMLGASCTLQAGGHVRVDVVYQRFSPR
ARAWVDALGHVIFTLPLCAMVGFASQDYVFESWVARETSPEPGGIPAVFILKTLLPVMAI

LLALQALSEIIKAVKTLISEVSHCD
>WP_040823729.1
MNDLAPSNIPYAYGGPLISGSLRDQPADFFVEENLGFEPEGEGEHVFLWIEKTDINTQQL
AGDIARLAKLPTRQVSYAGMKDRRAVTRQWFSVHLPGQDNIDWQALNSGQVRLLKQVRHL
RKLRRGAHRGNRFVIIINDVSGDTSQLASAVATIARRGVPNFFGEQRFGYGGSNLMRARQ
LFSGQFKPKKHQRGLYLSAARAYLFNQVLAQRVEANNWDQLTSGELLMLNGSHSVFAQGD
TIDLDARLLDGDIHLTGPLYGKSGSLAPTAEVAAMEADILQATPDFTAGLLQAGLKAERR
ALRLLPVDLQAQLSERQLTLSFALPTGCFATALVRELVNYTETHNHV
>WP_040811348.1
MYPLTRSLLVLLLSAFVSACATSPPSDTSNVCAIFREKSGWYNDAKKARARWDTPISVMM
AIMHQESRFVATAKPPRKKIWGIIPGPRPSDAYGYSQAKDATWEWYERSSGSYGADRDDF
GDAIDFIGWYNDMSFRQNGIAKDDTFRLYLAYHEGHGGFKRKTYRDKQWLVDVARKVDGR
ANTYNTQLKGCVKSLEDDKWWDFF
>WP_040542916.1
MIRIMLSVAVCVAQQAAADDINGVWKHADEPGWIEIQLERGSGTVERNDKFPERVGREIL
KDLATGGEAQTWSGLIYVEKMGEYKNADIILASPDRMKITVKVGFMSRTIGWQRVDEVPA
AP
>WP_040542885.1
MRILLVEDDVQLGESLEAALRLEHYAVDWLRSGEPVRATIGATPYDLMILDLGLPEVPGI
QVLRQTRADKHDIPVLLLTARNTLDDKVDGLDSGADDYLTKPFEIDELFARVRTLLRRRG
EGRSQQLEARGITIDPVDRLVIFEGEMLDLTAREYAILEILIRNAGRFVSRPRLEEGIYS
WGEEVGSNTVEVYISRLRKRFGSDCIETMRGVGYRISQ
>WP_040542483.1
MSFVHKLVQTIDAFTDRSGRVLAWLALAMALLITAIVIMRYGFNTGSIFSQELVTYMHAT
LFMLGTAYALKHGAHVRVDIFYRQFSARGKSWINALGGVVFLIPLCLFIVGVSWNFVNES
WAMRETSSELGGIAAVYLLKALIPLMGINLLLQALAETLRSTLELVEGNT
>WP_040541029.1
MNRVTVLLSAIALLIAAISVYLSFRLLDPTPPKTLILATGTAGSAYEEMGQSYRKILKES
GVEVQLLASGGALENLELLKSGQADIGFLTMGYPAGQDAVNLRSLGAMFFEPLWVFTQDN
DLLEGNLDSLRRTRISIGPSKSRSNSASRKLFELNGLEISDLNLFELDPTTAAQQLKQGT
LDTLFITGNSISPVIKQLLSSRETVLVDFKRADAYVALFPELTKLVLPAGVGDLALNMPS
SDTRLLAFTAMLGVNKDLHPITQSLVLEAAERIHAKPDLFHQAGVFPQARDQLIILSDSA
KAYYADGRPLLLRLLPYPVAVLFMQLIAAAIPLLGIAYPMFKLLPSAFHWIMRHQFYRVY
SELRQIDRSIGNITEAELKTHLERLENLEQKVTGLKVPITYSTMLYALKGHIGSVLKRVR
DALG
>WP_040541869.1
MNEALQQVINRNDTWQGHLAGQVLSANGDSHWDEDRLSTGYSTLDKELRSDGWPLGSTVE
VLSDGCGLGSMGLFLPAMEKLSAEGRWQVFIAPPFTPYAPLLKARGIDTDQILLVHPKSR
EDLLWATEQALRSTTSSAVFSWLGADEYSYSELRKLQLAAASGDSLSVLFRPQEAARNHA
PASLRLQMREYRKVHILKQRGGNQYIDVTLPPSEDVPEHPQLWEVPSWQASPGQASPGQA
SPKAQPAFSFA
>WP_009773271.1
MISHHYRRAMLPIATLGIAGMVLSGCASGSGGTGGPGDAGDPDGIVTIYGTIADTEAELL
EESWADWESENGIDIQYEASKEFEAQISIRAQGGNAPDLAIFPQPGLLADLASRDYIQPA
PAGVQANVDEFWSADWAAYATTGDTLYGAPLMASVKGFVWYSPADFADWGVEVPETWDEL
LALTQTIADKTGTAPWCAGFGSDAATGWPGTDWVEDLVLREAGPETYDKWVSHEIPFSDP
AIVSAFDSLGEILLNPEYVNAGFGDVKSINSTPFGDPARALGDGTCALHHQASFFDGFIQ
DPKNGNATVGPDADIWAFVTPSVEAGGNAVTGGGEIVGAFSNDEETIAVQEYLSSAEWAN
SRVKLGGVISANNGLDPASASSPILQQAITILQDPDTTFRFDASDLMPGVVGAGSFWTGM
VDWINGKSTEDVLSTIDASWPSE
>WP_009773251.1
MKRTATSVVAAVAAVGLTISMSACSTTSAAGSGDAEGPLTVWVMGDSGANFEMLVADSGI
EVEVVAIPWDSIDEKLTTAVASGSGPDILQIGLSKLRTFADAGALLPLDDEIANHPGIDP
ANFPAGVSGTATSVGGEIVSVPWTSDTRVLFTRTDILSEAGIDAPPATWDELRADAKTLA
ARGDDQYGYYIPQWDAPLPIEMTWSMGGEVIDADGNVNFDTPEFQKAVDVYTGLYADGSV
PVNGDFDQTQGFISGVAPMLVSGPYLGRGIADSAPELDGKWQASPLPAGDGGSISLFAGS

NLGVWFNTDQKETSLDLLEYVSQPEQQLEWYSMTGELPTVSSALEDGDLNSDPNVQVYTD
QLKTAKVLPLVSNWDGAVGTELLNALNAIVLTGADTKSSLDGLYSTTAGLTIN
>WP_009772227.1
MTDQNTNPTAPRRVVVAEDESLIRMDIVEILRDAGYEVVGEAGDGETAVALATELRPDLV
IMDVKMPQLDGISAAERLSANHIAPVVLLTAFSQKELVERASEAGALAYVVKPFTPSDLL
PAIEIALSRYAQIITLEAEVSDLVERFETRKLVDRAKGLLNEKMGLSEPDAFRWIQKASM
DRRLTMHDVSQAIIDQLSAKK
>WP_009771998.1
MHEIICPHCKKAFTIDEAGYADILKQVRDREFKTELHAQLALAEKEKIIAVELAESKIAS
GLGKEAAKKETEIEHLKAELKATDMEKQLAVKDAVSAVEKERDEAKNEREKNVVEKDAAI
ELLRAELKSTELAKQLAINEALSAVERDRDDLVRNLKATEIEQKLLESTLKEQHSTEVRI
LTETIDSYKDFKARLSTKMVGETLEQHCEIEFNRLRSAAFPDAYFEKDNDAKSGSKGDYI
FREHSASNVEIMSIMFEMKNESDMTATKRKNEDFLKELDKDRNEKGCEFAVLVSLLEPES
DLYNGGILDVSHRFPKMYVVRPQFFIPIITLLRNAALSTVQVKLELARVQEQNVDITKFE
GNLRAFKEGFSRNYSLAADQFQESIKRIDEAIKDLEKTKENLLKSSNNLRLANEKADGLT
IKSLTRGNPTMAAKFAELESPDDPENFK
>WP_007235241.1
MKILLAEDDAQVRTELRELLVEQGYDVTTAIDGIDAFEKFRADEMIEILLLDIRMPRATG
IQTLDAIKKIESANERVFETLFITGASDNNAIVSALKLGAFAFLFKPIVVEELLKELSEA
TDSINQKHYRNFQNSMFNPNLESRAPGKSGSIKEGMGATSEIAAIGAEHYAPGIEQHVHR
ISEMALCIAVRLGLETQHCQQIRLASLLHDIGKLGGPTDIYTAERALTEEEFEKTKDHTR
LGAALLEHYDDPIIEVAQNIALQHHENWDGSGYPAGLKGDEISIEAAIVHAVDTYDNLRS
HRPYRAALPHHVAMEILVSGDEKSNPDHFHPGVLQALLSQHREIEAIYERYRPIDVESKI
TDQTPA
>WP_007235034.1
MKVLVVDDESDIREELGEFVEQLDFSVVLASNGEEALGKYFDDPEISIILSDLMMPGLNG
LEMLDNINSAPGAHQRVRRVIFMTGNGNTQSVIRAMHLGAKEFLLKPVDLDQLERHIMSA
KREVATDRARQTEEILLKQQVSLNNAQISSLNRDVEEAYAEALACLAAAAEHKDPETGQH
IIRIGEYAAVLAQALGWDKEWSEMIRLAAPLHDVGKVGMRDSVLLKEGPLDDDELHHMRQ
HPETGYQILSVSNYPTMKMAARIARCHHERWDGTGYPRGLKGSEIPVEASITTLVDVYDA
LRSKRPYKPAFDHKQVMDIILNGDGRTEPKHFRPDVLAAFEAAQDKMADIFERLSDDTDS
SSHTSSSKREVL
>WP_007233471.1
MSELLIDVGNSAVKWAVCHGLNLKSQRHSGSFSDLAEAMWTSAKGDSTVWIASVRDEQSD
QVLVSELHAVGFSNVHLCGTAQKEDGLLNSYAEPSRMGADRWFAMLGARACKRGPLLVID
AGSAVTCDLVASDGRHLGGYIFPGPALMEAALQSNTQKVRYSDSLKLALDPGQSTAECVA
SGISVAMLGAIKQVCDQYPAHQVIFTGGAASGLNAVGLVGDWRPDLVLEGLLSRAHGTEV
AFAT
>WP_007230922.1
AAFHYFYMREVWVMSGDTPTDFRYIDWLLTVPLLMIEFYLILAAITKVAGGVFWRLLIGT
LVMLVPGYMGEAGYLNVTVGFGIGMLGWAFILYEIFFGEASKVAANEAPAAVQKAYTLMK
WTVTIGWSIYPLGYFFGYMAGGTDVGVLNIVYNLADVLNKIAFGLFIWYAANEDTSAKA
>WP_007230850.1
MKILLVEDDLATREEVSDLLETLGHHTVDSDCAEEALQYIRSDGSADLILFDLNMPGTSG
LDMIREVRHTSTQSISTMPAICMTGSRDAHSVVELLKTGITDFLFKPLRLADLKSSLQKV
ESEISRVRAQESQAAALNDKLNEKDKLLEELSLELSESQTESVLCLAYAAEYKDLGTGAH
LRRISKYAERMAELLGWSEERCSSIALAAPLHDVGKIGIPDTVLNKTGTLTSREFNCLKS
HTTLGAEILSASKSPVMRLGAKIAHYHHENYDGTGYPSGLVGSQIPIEAMITAIVDVYDA
LRTSRPYKEAMDHASAIDTMCNGDERTDVSKFHPELLKTFLHNHHDFGEIYRKNTVADVP
PIMLSAVAH
>WP_007230835.1
MSAEFDLKRSCFGDFMKILVVDDEPLIGSETSEYLSLHGYVSDHCHSCDAAMEILSSDPD
IRLVITDLRMPEKDGFTLIEATQPLQRHIEFIVVTGHGGKDEAINAVRTGVSEFLAKPVN
HFELLKAVKNAAQKIADHDHELSVKSSLQGKVFAGEKKIDRLLGNLDTSYAELTYCLATA
SEYKDPETGQHISRIGSYAALIAGLMGWTERKVEMIRLAAPLHDIGKIGTPDSILLKPGK
LGDVELRVMRTHSSIGHAILSQSTSPVLKMAANIALAHHERWDGSGYPNGLSAEEIPVEA

AITALADVYDALRSKRPYKPALDHRTTCDILLYGDGRTEAKHFSPELLQIFKENHDKFDE
IYESMHDQAVSC
>WP_007230577.1
LSLTGTDLEVELVGRVKLASAGDSGELTLPARKLVDICKSLPEGSEISFAAEDSKVTVKS
GRSRFTLSTLPAREFPNVEDSMGTHQFTIKQGQLKRLIDRTGFAMAQQDVRYYLNGMLWE
LKDKQLKVVATDGHRLALCTLPEKIEAGDDAQVILPRKGVLELARLLLAEDEDIAIVIGS
NHIRATTEEFTFTSKLVDGKFPDYQRVLPRSPNKIVLGSRLELRQAFTRTAILSNEKYRG
VRLKLTDNSLDIVANNPEQEEAEEAVPVDYQGESLEVGFNVSYLLDVLAVLSGEQIKLSL
SDPNSSALLEESDEGDSLYVVMPMRL
>WP_007229328.1
MDPIGTALVVDHDTARLEALASLVSSLGFTPENYTDANAAREYLSTRPDLDVILCEMDME
GLTWDSAHRSLQEMDVQIPVILFSDEAQASRMMRALRFGASDFFVRPVDDVEALQRSLDR
CVRQRQVRRELEQSRQRLQAANTELRGTIHVLEQDQQAGRQVQMRMLPATPLVLNDYVFS
HTVIPSLYLSGDFTDYFTVGDHFVTFFMADVSGHGSSSAFTTVLLKNLFARKRSDFLRQN
DDTILSPLALLKRANKEVMDLEVGKFATMVVGVLDMKSNNLRYSVAGHLPQPVLVSGDGA
RYLRGEGSAVGIMDDASYEEHMIDLPDSFMLALFSDGILEILPPKNLIEKEKYFLNVFEE
TANSPEEMVTRLGLDQADTAPDDIAALFISKRN
>WP_007228310.1
MSLCLQLDVGNSSAKWRLLEQGDVLSRGRYSAADANTQRELLESTASVDQIWVSSVAGGD
TEAELKEMLEQRWGVTPWFARTPAATGDLRNSYADPARMGVDRWLAMLGARARCGKRVCV
VDAGSALTIDLISATGQHEGGYIIPGPALMERALLLDTDRVRFTDEVSYDLAPGSSTAEA
VRHGIAVAQVGSVSIVLDGCASEPPALIFCGGAGQVLQQLLDRGGEFIPELVFEGLEIMA
AAP
>WP_007228237.1
MKNVLQHLTRIRDWWIALCTGRDPMRPLDEEHYRQRILAITSFFCLITVIGVPVVIPLVI
DISPQGRFAATTLLAIIGLSVLVSVLILRYLNNRIAALHLLLLVYTGAFAIACAYFGGTR
SPTFALLILAPVMASVVGGTGAGLFWTALVLIIWSVILGLERLGVQFTQIILPQNYNMAI
TLSYGAMGLSVISIIKVYAEMNKHLREALQGANSELEFLSNHDDLTGLYNRRFYEQRMAH
LLERAEITGKTIGLIMFDLDDFKQVNDTHGHGMGDALLKMLGERLRHQVRDIDLIARLGG
DEFVVLMENMRSSDDLPEIAAKLVAAVEQPVKARNEVMALKVSCGFALYPGDGLSRAELE
EKADKAMYRAKKRGSSPDPSLILH
>WP_007227932.1
MTARVLPFVLLLLAGCTTSPPSNVNNICEIFEEKSGWYGDAHDAKKEWGSPIPVMMAIMH
QESRFVAKAKPPRKKIFGFIPGPRPSDAYGYSQAKKSTWKDYKRGGGNYGADRDDFGDAI
DFIGWYNEQSKKRSGISKRDTYGLYLAYHEGHGGYNRRTYKSKKWLTDVARKVERRAGSY
QQQLSTCEKDLEKGGWFFGW
>WP_007226969.1
MILECDIGNTRCKWRVVGEGAEENRGAFDCADGFGELPSLDGIRRVKVSSVAGSTVNEEL
TRTLASAKLEIEFARTSPLKAGVENAYADASKLGVDRWVAMIAGYNRCRGPVLILDAGSA
LTVDLVAANGKHLGGYITPGIQLMKSSLLAETDGVRFDRDNHSSGTAFGTDTASAVHAGV
VAAQVGAAIVAIEEAGRKVSAGFAILLTGGDANVICTNLPATISAEVTMVPELVLDGLQW
VLP
>WP_007225387.1
MTTNLSASDPVGMSFWLISMAMVAATVFFLIERDRVSGKWKTSLTVAGLVTLIAAVHYFY
MRDVWVATGETPTVYRYIDWLLTVPLLIIEFYLILSAITKVPVGVFWRLLAGSLIMLGAG
FVGEVNPDYVVSGFVVGMLGWVWIMYEIFLGEASKINAASGNAIAQKAYGAMRLLVTVGW
AIYPIGYVLGYFTGSTDSATLNLWYNVADLWNKVAFGLVIWAAAVADSE
>WP_007225373.1
MNEIKCPHCKKAFTIDEAGYAEIVKQVHNSEFDQQLHERLELAERETRNAVKLAEEQARS
KLLEAESTKNDEIRKLQSELEAGDYARKLAVSKALKAVEKERDELANKLIQAKNDSTNAS
KLADANHSKKLEKTSAEKDAAIRLLEEKLSASEDTKNHAVTKAVNVAERERDELKSKIYR
TKLENEIAETSLKDNYEAQLKDRDHEIERLKDMKARLSTKMVGETLELHCETEFNLIRAT
AFPRAYFEKDNDSRTGSKGDYIFRDSDEHNTESVSIMFEMKNEIDETATKKKNEDFFKEL
DKDRNEKQCEYAILVSLLEPENSLYNSGIVDVSHRYQKMYVIRPQFFIPIITLLRNAAEK
SLKYKKELAVVKEQNVDITNFESELDEFRSGFARNYELASKKFKTAISEIDKTIDHLQKT
KDALLGSENNLRLANNKADDLTVKKLTKSNPTMEAKFKELNNDEDI

>WP_007225063.1
MNIKQWLNPFDLVVSVRTGFALIIIRRALLLFAVLSAPLAFSADISGVWKHSKNTAWIEI
SLADSSATVLRNDKFPERVGRTILKDLQVDTSTQGLWHGLIYVEKLGDYKDVEVSLPEAG
RMLLKGKVGFMTRTVEWLRVDNIR
>WP_007224727.1
MKLLLVEDDEALAKALLIALRNEGFSVDHVATGNEAIAHGKNNIADIIILDLGLPDIDGL
TVLKELRANKIVTPMLILTARDDLSDKITALDGGADDYLSKPFEIKELMARIRALGRRMN
SSISSVITAGRVSLDSANHHVEVDAIETPLSRREYTVLKALMENVGRIQTKAALENKLYS
WGEEISSNAIEVHISNLRKKMPEGFIKTVRGVGYTIDRSSS
>WP_157361222.1
MAALGVVAALALASCSAGDGGSGESATGDLRVWLVGTDTPQEARDYLIDTFESENPGSTL
TIEEQAWGGLVDLLTTNLSGSDSPDLVEVGNTQAAAFTSAGAFLDLTADYDALGGDDLLP
GFVEAGSYDGKFYAAPLYSGSRLVFYKKDALAAAGLSVPTTLDEYVSNGEALAEANPGAS
GIWWPGQDWYNALPFIWENGGEVATFESSGWKSQFSSPGSIAGLKQVQDVMTNASRAPKD
ANETNPQVGYCEGTTLQLSAPSWVKWSILAPLDAETPGCPDEEANLGVYAMPGKDGGAAQ
VFAGGSNIAVSAKSAHPELAKKALAIILSDGFQEIYGANGLVPAKLSLADTLGTDEVAAA
ISEAAGAARLTPPSPKWADVEASGALTDFFVQIAQGGDVASLAKDLDAKIDSILNG
>WP_157361184.1
MVQNRIGRAVLAAAAAVAVSIAMVGCAPASESEQGEVELRFSWWGTDSRHELTNEALDLF
EEKNPGITVVRDFGGFDGYIDKLLTQAAGNNSPDVFQLYEEVLREFASRGQLYDLNEATS
QGLSLDGWDQGLLDTSTIDGSLSALQFGLTTQAFIFNTELFDQAGVSIPTEGWSWDDLAT
AAKKVSDGTDSGTFGVTDLSTGYQVFEVWANQNGESYLTDDGLGFSAGTLEDFWNYWADL
RASGAATPGSLTSEYPTPFDAIIASKAASGFIFANQMAAVQSSIEAEIAVDRMPGESPEA
GSYLRTAMNIAIGSKTEHPKEAAMLVDFLLNDPEAYAILGIDRGVPANPAVGDAATANVD
DITAKGLTVIDGVREDGAAPPVPPKPGAGNVNALFAELAQEVQFDRMSIKDAVASFIERA
EQELS
>WP_156788299.1
MSRGILGPALVFMVMAIICLSLILPLRQLLIAGTLIVCTDLAVILLYFSGAFVINIDPVQ
AFAAPASWFSYSIVPIYASILIMLVLSRFNKGIEQVLTELEEEKSAAFYLSEHDHLTGLP
NMRVMEIRAHQAILMADRGESKPALLFIDLDHFKVINDKRGHDVGDLVLQEVAIRIQSVI
REGDIVARAGGDEFLVLLPQANDTVDAETVSQKICVTLANPFRLDDDEIYISASFGIAMW
PEHGRDLKSLTRSADQAMYAVKTSGKNGFKVSTSSIENG
>WP_156788178.1
MGDDAGNKTFGSLLKSELRDTDVIARTGGEEFALLIRTDSYSVTIIKAKMIC
>WP_157361215.1
MPVWFEVTSLSILLLILAADLIMAYKRPHIPSTRESALWVGFYVSLALIFALMMFLLGDV
EQAGQFIAGWVTEYSLSIDNLFVFVIIMARFSVPRKYQQEVLMVGILIALVLRGIFILLG
AQLIENFSFIFYIFGAFLLYTAIRQVFENHDDMEETESGIIRFLRKHINISPVFDGGKMR
TVIDGKKVFTPILVVFVALGITDLVFAIDSIPAIFGITTDPFIVFTANIFALMGLRQLYF
LLGDLIDKLEYLHYGIAFVLGFIGVKLFFHALHINELPFINGGEHVEWAPEIGTWTSLIV
ILVSMAVSVIASLVKMNVDKKRELVSIDSE
>WP_157361178.1
MLSSPTFCWVLSRPQDPCQGLPAVITAHYCCGVSDTNNTPDIVPFVARRLLTADQQRTAV
AVAAPVGAPSSASPTSLGVDPGSQQLTAQILHEFGPLAPYVGRPTITDVFVNGAQQVWVD
RGGGLEPVNDLGLTEPELRALAVRLISLGGRHIDEATPCVDVRLAGGVRVHAVLPPISAT
GTLLSIRIPSREPFGLAELDLAGFFTEVPMQRVKGLVDARENLLISGASGAGKTTFLGAL
LGAASETERIVAIEDVAELRVEHAHFVSLEARQANLEGTGSYGLPALVREALRMRPDRLV
LGECRGAEIRELLSALNTGHDGGAGTLHANSLRDVPSRLEALGALAGLDASAIARQAVSA
IGAVLHLDRVGGRRRLTQVGRLILDENERLAIADDE
>WP_157044971.1
MVTAAIGAGDPAQGRALLWLNVAALLLVLLSCFAVIHSTHATRELYTQLQVLESRQWHLQ
EDYGRLLLEESTWASHYRVEKVARTELGMAEPDLAHYKVVRR
>WP_156788350.1
MVLSSLNVARAEVAEIVAAPIEFPSTMAWSAYNLGTTGYNQAVAIGKVLKDHYDTNLRVL
PGKNDVSRLLPLQRGRVQFSANGAATYFAQEGVFQFAEKQWGPMPLRIVMASNGETNQAL
GVAADMGIATYSDLRGKRVPFVRGAPALNVTTEAYLACGGLTWDDVERVDFPGYSAMWTG

IVNDQVDAAYGTTVSGPTRKLEASPRGIFWPPAPHDDAECWARMAKIVPFFQPHMATRGA
AISIANPHEGATYPYPILITLAKTDPDLVYDLAKVIDIHYDEYKSADPGSIGWAMDRQVF
RWVVPFHGGAVRYFKSIGVWDDATQAHNDRLIGRQDVLAKAWRVHKASYPGKEGFADAWG
KARVQALDANGFDPVWR
>WP_156788318.1
MKVPRLLTKLILCALFTSLVAAGFATKNLRDFLNTPMDIQGEGLAYLLEKGGSLSQVGVD
LSLLGVLENRRWLSIYSRISGRGTAIEAGEYWLEPGLTPLELIAKFEQGDVRFFQLTLVE
GWDMSQVLSRLRSADALINTFGADTRVLTADMLGLETSFPSLEGLLFPDTYRYHSGTTDR
ELLLQAYQRMQKVLNDEWSDRSKNLPYDNMYQALIMASLVERETGVAWERAQISGVFVRR
LKLGMRLQTDPAVIYGLGASYTGNLRSRHLKDGSNKFNTYRHHGLTPTPIALAGREAIHA
ALHPADGKTLYFVAKGDGTHYFSETLKEHQKAVRKYQIEQRRKDYSSTPVIKPAG
>WP_007223442.1
MDLATVIGVVGALAIIITSMVLSGGIGMFTNMSAVLIVFVGSMFVVLSKFGMSQFLGAGK
VAAKAFFFKSTDPSAMIDEIVVLADAARKGGLLSLEGKEVGNDFLQGGIQLLVDGHDPDV
VKALLSKDKDKTVERHEQGASIFAALGDVAPAMGMIGTLVGLVAMLSNMDDPKSIGPAMA
VALLTTLYGAMLANMVAIPISDKLILRRGEEEMNKSLVIDALLAIQSGQNPRVIDSMLRN
YLPASQRPQADE
>WP_009774002.1
MVNAGIRIGVVGATGQVGAVVRRLLEERDFPVAEIRYFASSRSAGTTLPFKGEQITVEDA
STADPTGLDVAIFSAGATTSKAQAPRFAAAGVTVIDNSSGWRMDPDVPLVVSEVNPHAID
QAVKGIIANPNCTTMAAMPVLKVLDAEAGLERLIVSTYQAVSGAGLAGGEELLEQAAAAV
AQNTMGLVEDGAAVTMPAPNKFPKNIAFNVVPLAGSIVDDGLNETDEEKKLRNESRKILE
LPGLLVSGTCVRVPVFTGHSLSINVEFARPLSPARATELLATAPGVSLSDIPTPLDAAGA
DPSFVGRIRADEGVPDGRGLALFISNDNLRKGAALNAVQIAELVAAKITAKVSA
>WP_007235113.1
MSEAIDIAVVGATGAVGEAMMEILEQREFPVGKLYALASERSAGKTVRFRGKSITVSDLA
EFDFSKTAIALFSAGGSVSEEHAPRAAASGCVVIDNTSHFRRQEDIPLVVPEVNPGALAA
YRSTRIIANPNCSTIQMLVALKPIYDAVGISRINVATYQAVSGTGKAAIEELAGQTARLL
NGQPTEAKVYSKQIAFNALPHIDTFEENGYTREEMKMVWETQKILEDPDITVNATCVRVP
VFYGHSEAVHIETNTKITADAARKLLQDAPGVTLTDGREDGAYPTAVTDGAGSNPVYVGR
IREDISHPTGLNLWVVADNLRKGAALNSIQIAELLVKEHF
>WP_007227922.1
MSELYDIAVVGATGAVGETMISILEERDFPVGNLYPLASSRSAGKTIMFNGNTVKVTDLA
EFDFSQAQIGLFSAGGSISEKYAPIAAEAGCVVVDNTSHFRRDEDIPLIVPEVNIEALAG
YMTRGIIANPNCSTIQMLVALKPIYDAVGIERINVCTYQAVSGTGKEAIEELAGQTARLL
NGQEAQCEVYPKQIAFNVLPHIDSFQENGYTREEMKMVWETQKIFGDHSIQVNPTCVRVP
VFFGHSEALHIETVDKISAEQARELLQNAPGVQVMDEQADGGYPTAVGDSAGSDPVFVGR
IREDISHPRGLDMWVVSDNVRKGAALNSVQIAESLIATYLD
>WP_009773280.1
MPDTHSERVIEDEIVTDFSERMSYGSYLELDTLLSAQTPQSTPEHHDEMLFIIQHQTTEL
WLKLVIHELTSARDLIANDNLSIALKRIARVKHIQRTLTEQWSVLATLTPSEYSQFRDYL
GSSSGFQSYQYRAVEFLLGNKNAGMLKVFESHPEAHALLSKLLAEPSVYDEFLRYLSRHG
YDIPEAVLNRDVTRGYEQNDDLIETFRHIYDNESEHWLAYEACEEFVDLEDNFQLWRFRH
MKTVMRIIGMKRGTGGSSGVGFLQKALDLTFFPELFAVRTEIGRS
>WP_007236374.1
MDMTIVYGVGMFTAIVLALVMVILAARSRLVSSGNVSIEINGGKTIEVPAGGKLLQTLAD
ANLFLASACGGGGTCAQCKCQVSDGGGSMLPTEESHFTRRQANDGWRLSCQTPVKQDMRI
QIPEEVFGVKQWECTVESNDNVATFIKELVLRLPEGESVDFRAGGYVQLECPPHNVNFDN
FEIGEEYKGDWERFGFFKYGSASEDTTIRAYSMANYPEEKGIVKFNIRIATPPPGSEGIP
PGIMSSWVFDLKPGDKITVYGPFGEFFAKETDAEMVFIGGGAGMAPMRSHLFDQLKRLNS
KRKITFWYGARSLKEMFYVEDYDGLQAENENFTWHTALSDPQPEDNWDGLTGFIHNVLFE
EYLKNHPAPEDCEYYMCGPPMMNAAVIKMLVDLGVERDNIFLDDFGG
>WP_007228144.1
MNAVQETLADWRDSLIDLFPDARPVRWVLLALVAYLLIAIIVGMIWSLPPDHFDPSEKAA
EYAAQDGGEVVTGSTTTAALMGVMETLLEKPGGYLHNDRFPPGIWLDNMPNWEYGALVQV
RDLSRAMREVFSRSQSQSTEDKDLAMAEPRYHFDSDSWILPSSESEYRQAQDYTRGYFRR

LSDSTQAEAQFFARADNLRYWLSTVNTRLGSLSQRLSASVGQRRINTDLAGDAGASQSTA
APREMEVKTPWLEIDDVFYEARGTTWALIHFLKALEVDFADVLAKKNARVSLQQIIRELE
ASQETLWSPLILNGTGFGLVANHSLVMASYISRANAAIIDLRDLLLQG
>WP_007223884.1
MDWQAIKERLVVWREDRFDTATESNSTKVVLIVAAVYLLLAISVGMYWSMMPAQFPVQEN
AIVMAERSQQSVAVGSTTTAALIQVISTLLDKPGGFMSNDVMPPGLWLDNIKNWEYGALI
QSRDLTRALRESFSRSQSQSKEDLDLGKAEPSLNFSSDSWTLPASESEYKSAVKHLNRYL
VRLEDTGNSGSQFYARSDNLRYWLAIVESRLGSLSQRLSASVGKRRLNTDLAGDSAAQQS
TSAPSELEIKTPWTEIDNVYYESRGTSWALLHFLRAIEVDFHEVLKKKNALVSLQQIIRE
LEATQQTIFSPMILNGSGFGVLANHSLVMASYISRANTAIIELRDLLSQG
>WP_009772683.1
MSMFGLKESEIATLVRAVSVDGGGNLDRASIENRFARATWSGLAEPGDRLAGRAIQQLGS
ARSLTAVVEHWDAEQFATELSADGDPVSGDDMRQAIDRWMPRLKSDTALIALRQAARFGS
RLLIPDDSLWPERLHDLDWHAPSALWVRGTDAALAGIVDGIALVGARAATGYGEHITMEA
SAGLVDRGYTIVSGAAYGIDGMAHRAALASHGLTVAFLAGGVDRFYPSGHDSLLSRIVEN
GAVISELPCGSPPTKWRFLQRNRLIAAASIATIVLEAGWRSGSLNTAGHAAALGRPLGAV
PGPVTSAASAGCHRLIRDYDAVCVTNPDQMAELAPLDRAPETDATMTTPESQPPTNLPPE
SSAPKVDADSPPSTETTRLLDALSVRSARTADDIASRSGLALATVRAHLGLLELDGRVVE
SERGWKQASQSRTA
>WP_007234196.1
MSYVLARNAAADQLWFEFLATKMLDRASKRKLLAQGFTPPQLLEPHGTWPFDGAELRASL
RVSKSRQVARLKPLLACHLLHWGRQSSGCDVYPPLLSGLSDAPLALFVSGDINCLSRPAI
AIVGTRRPSRDGLKLADQMGYQLAAAGFLVVSGLARGIDAAAHRGALRSGGQTLAVMATG
MDRIYPSEHYRLAEEVAAQGALLSEFCPGVVPHRGHFPRRNRTLSGLCLATVVIEAGHPS
GSLITANAAVEQGREVFAAPWSLFHRGGAGCLRLLSQGAQLIDTPAAVIEHLAVHLSGWA
ELTADALDYSSNAGETVSLAPLEPAKRQLLTLLGDGEHDLASLASALQCSSRQLLAMVTQ
LELQGYVEQTSAGLRAVRHP
>WP_007224080.1
MKDDETRAWLALSRIPHLPRRVLHRLILATSSAEEIFQLSAFELTAAKVGAEAQKMLREG
VDLRQVEQDFKTVQLQHIKLLPVSSTLYPALLKEINDPPPLLYIRGDLSVLDLPSLAMVG
SRRSSQAGGANAFRFARELAGAGFSIVSGMALGVDTQCHRGALAAGGSTVAVLGTGIDIV
YPRRNKELFESIVCQGAVISEFPMGTDPHPARFPRRNRIISGMSLGVLVVEAALQSGSLI
TARCAMEQGREVFAIPGSIHNSGSKGCHQLIKQGAKLVESVSDVMEELKGWCADAPPVLE
EKGAQNKVSKDLHERERLLLDIIGYDPVSIDSLQQRTDWPMHDLVALVTALELRGLLDCV
AGSYQRTV
>WP_007236375.1
FLAVSKKIQAAFGLGVAVVVVLTITVPVNNLIYQYLLADGALAWAGLPDVDLSFLGLLSY
IGVIAALVQILEMFLDRYVPALYAALGVFLPLITVNCAILGASLLMVERDYTFGESAVFG
AGAGVGWALAIVALAGIREKLKYSDVPDGLQGLGITFITVGLMSLGFMSFGGIDI
>WP_009772673.1
MADFSLEDLKTLRERLGTGMVETKNALVEAGGDLEKATELLRLRGAKSNAKRSDRSTSEG
LIAAQSSGTSTTIIELACETDFVAKSDKFVALGEAVAAAVAAAGASTVEEGLAAPAGSST
VAQLIDDEAAILGEKFELRRLTKLEGDSFEVYMHRTNKDLPPQVGVVVAYSGDDAETARG
IAQHISFAAPTYLSREEVPADDVENERRIVEEISRGEGKPDAALPKIVEGRLGAFFKQVA
LLDQDYARDNKVSIAKVSADAGITVTGFARFKVGA
>WP_148224520.1
MMSVRWTMPLLIAAVLMSSFAIIHSTHASRAYYANLQRLEGTHWYLQEDYSRLMLERSTL
ASPHRIAKMAQDELIMRAPDLATYRTIVEGAY
>WP_148224355.1
MIVILSGIVICFTLGKQALHAPMNLPQPDATVIVEQGDSLKQILTKLKSRGFIESSRLLE
LWARWQGVDRQIHTGEYLLVPGLSGIGFLERLGRGDVLSYKITLPEGITLQQALQRLHDD
RRLVRELRDAHDPLLLELVSPMTSPEGWFLPETYRFVAGDSDYDILRRAHHLMQRELIRV
WEARSSDTPLMTPYEALTLASIVERETSVAKERATIAGVFSRRLQAGMRLQTDPTVIYGL
GSDFDGNLKRRHLKDAANPWNTYRIKGLPPTPIALPGVAALEAAVRPASGAALYFVARGD
GYHVFSETIEEHNAHVQRYQLSRKVDYRSTPKGGD
>WP_007229556.1

MSNQQLGDFLAHSVELESEARERYLELAQAMIAHHNTDVAGFFNRMAEESRLHLEEVAEI
AQDIELPGLKAWEFGWPEEESPEAVSYEAVHYRMSLRQAMLLALENERAAEKFYRSFADA
SSDGETRHLAAQFSAEEASHAAQLEKMLGKLPPDREHHLEEDDQPHMPE
>WP_007234052.1
MSKTNIFVGVFLSLLLGAASQAQSSDLTNPTALVDNGPYTGDLQGLVERRIVRVLTVYGP
GRYYLDNGAKGVTAEYANRLEKVINESFDTGHLKVAVFVLPVARDELFLALEQGRGDIII
AGTTITPAREQRAAFTIPSSKPLKEILVMGPSAPPINSIDDLSGKSVYLRASSSYSDSIA
TLNERFTREGKALVTVEPMSELLEDEDLIEMVDAGLLPWTIVDDYKPTQWSGVFTNLTVR
NDIIFRKGSRHAWAVRQDNPELKKFLNNFLKDNKEGTLFGNILKNRYVRDFNWAANTNAE
SELQRYRDLEALFRRHGVSYGIDPALLAAQGFQESRLDQNVRSGAGAVGVMQLLPSTAAD
KNVGIPNIHEVDPNIEAGAKYLAFLKRRYFSTPGMDPLNGALLALAAYNAGPAKVRRLQE
TARTRGYDPYRWFDNVEVIAAEKIGRETVQYVANIFKYYLSYQMINRESARREAARQAAG
APTHEQRQN
>WP_007235497.1
MIKPRGRRIGLIRLSAIGDVCHAVATVQALQRHAPEDDITWIIGRTEAALVSDLPGITFI
VFDKKQGLTAFRNVLNEIAEPFDVLLHMQVSLRANILAAVVPAKAKLGFPKHLSKELHGM
VVNRRVPMPETPHVLEGFQHFAYALDVPTFAPTWSIPISEADQAWVRERLTAQKPYVVIA
PSASNAERNWLVDRYAALANHLQYRGYNVVLTASPAPSEVALAQQITALAGSNIINLAGQ
TTLKQLLAVVADATLVVAPDSGTAHMAVTQNTPVIGLYAHSNPNRTGPYRFQFLTIDAYQ
KNLQHLFSNSAKSNKWGVRLKGAHLMEDIALSEVIAKADEVLSEAPNPSDHNS
>WP_007235565.1
MFKIRTFNAISVKGLERFPRQSYEVGGEIGSADAMLLRSHKLQADEISASVTAIARAGAG
VNNIPLSHCTELGIPVFNTPGANANAVKELVAAGLLLASRDILGGIDFVNSLSEDLDEQA
MGPLLEAEKKRFAGAELKGKTLGVLGLGAIGSLVAQLGLELGMDVVGFDPAISIEAAWQL
PSSVKRMENMQALFSRADYISIHVPAIESTHHLINQETLKYFRSDACLLNFAREQIVDTE
AVAAALDKQGLGRYITDFPHPLLRGRKDCILMPHIGASTAEAEENCAIMGADQLRAFLEH
GNIRNSVNFPRLELERTTGSRIAITNTNLPGTLSHILTAIGDSQINVVDLLNKSRDEIAY
NLIDLNTTPPADLLEQLRGIEGVINVRCIPDQAAD
>WP_007227712.1
MAKQVLTLNQISLKGLERLPRDSYEIASEFSHPDAILLRSHKLQAQDIADSVLAIGRAGA
GVNNIPVAECSQRGIPVFNSPGANANAVKELVAAGLLLGSRGIVEGIQYVDTLSAMADKT
EMNKTLEAQKKQFKGSELEGKTLGVVGLGAIGSMVAEMALTMGMDVVGYDPALSVEAAWR
LSSQVRKADTLSALFGRCDFITLHLPVLDSTRGLINAELLSSTREGTCLLNFARQEIVDE
EALVQALDGDKLRKYIADFPSPALIGRDNVILMPHIGASTDEAEDNCAIMAANQLKDFLE
NGNIRNSVNFPNLSLERVSGCRLSVTNENVPKILGSVLSILADENINVIDMLNKSRNDIA
YNLIDVVGHTSDEVLDKMRALEGVVNVRMIGDCA
>WP_007226314.1
MSSISKTVAKTFGLILFFISIANAEITGIVVSVTDGDTIKVLDENSNQHKVRLTGIDAPE
RGQPFGQASKKYLASMVSGKEVFVESNKKDRYGRVLGKVWVQPADCPSCGKTLDINHAQL
LAGMAWWYRYYAKQQSPEDRGRYESAEDEAKARGWGLWSAASPINPYNWRKGRR
>WP_007229969.1
MSATKPDLVWDRIRTETQKHAQEEPVLASFLHSTILNHDSLECALSFHLASQLDSPTVTS
LLLREVMLQAMRADDAIGEAIRADLLAVVERDSASHELYIPFLYFKGFHALQSHRIAHWL
WHNNRKSMGLFFQNRISVEFGVDIHPAAKMGQGIMLDHATGLVIGETAVVGNNVSILQSV
TLGGTGKQDGDRHPKIGDGVLISAGAKILGNICVGDGAKVGAGSVVLEDVPPHTTVAGVP
AKVVGRPATNAPALDMNHDFFCDSGDVEG
>WP_007228043.1
MEELLSLFIRSIFIDNMALAFFLGMCTFLAISKKIDAALGLGIAVIVVLTITVPVNYLIY
NYLLADGALAWAGQPDLDLSFLGLLSYIGVIAAIVQIMEMFLDKFVPALYNALGVFLPLI
TVNCAILGATLFMVERDLDFAESVVFGAGSGVGWALAIVALAGIREKLKYSDVPDGLKGL
GITFIIVGLMSLGFMSFGGIDL
>WP_007227945.1
MDMTIVFGVAMFTAIVLALVAIILFARSALVSSGNVSIEINGEKTITVPAGGKLLQTLSE
SGLFLPSACGGGGTCAQCKCIINDGGGSMLPTEEGHFTKRDAAEGWRLSCQTAVKQDMKI
EVPEEVFGVKQWECTVESNPNVATFIKELTLKLPEGEHVDFRAGGYVQLECPAHHVKYSD
FDIEEEYRGDWEHFNFFKHESVVKEDVIRAYSMANYPEEKGVVKFNIRIATPPPGSEGIP

AGQMSSWVFNLKPGDKVKVYGPFGEFFAKDTDAEMVFIGGGAGMAPMRSHLFDQLKRVHS
DRKISFWYGARSLREMFYVEDYDMLARDNENFDWHVALSDPQPEDHWDGLTGFIHNVLFE
EYLKNHPAPEDCEYYMCGPPMMNAAVIQMLIDLGVEPENIMLDDFGG
>WP_050774034.1
MEVIVGEPRKPLPRGSDFYTPEQRLRRDSSVWTTVQGVLAPLQFLAFALSLVFVINFLAN
GTGYSAAVISVLIKTLFLFTIMVTGAIWEKVVFGRYLFAPAFFWEDVVSMLVIFLHVAYV
VSWLFDLQAPREQMWLAIAAYTAYVINAAQFLLKLRAARVGSSQNNTDSVNEYAVEVSR
>WP_040823654.1
MLLAGVDEVGRGPLAGDVVAAAVILDPANPIRGLDDSKKLTEKKREALFPEIQEKALSWF
VARASVREIDELNILQASLLAMKRAVEGLVLQPEHVLVDGNKLPRWAYSAEAVVRGDSRV
QVIGAASILAKVVRDREMVAFDDEYPGYGFAGHKGYPTRVHMTALDVLGVTPIHRSSFGP
VKRKIAQMNRP
>WP_009772453.1
MATPNPLDAVINLAKRRGFVFQSGEIYGGSRSAWDYGPLGMALKENIKKQWWQTIVQGRD
DVVGIDSAVILPRKVWEASGHVEVFSDPLVESLHTHKRYRADHLLEAYEEKHGHPPVNGL
ADIRDPDTGQPGSWTEPQNFSGLLKTFLGPVDNEEGMHYLRPETAQGIFTNFANVMGAAR
MKPPFGIGQVGKSFRNEITPGNFIFRTREFEQMEMEFFVEPGTDEEWHQYWIDESMKWYT
DLGIKPENLRFYEHAQEKLSHYSKRTVDVEYRFRFAGSEWGELMGIANRTDFDLRTHSEA
SGADLSYFDQAKDERWTPYVIEPAFGLTRALMAFLIDAYAEDEAPNAKGGVDKRTVLRLD
RRLSPVKVAVLPLSRNERLSPLARSVAADLRKFWNVDFDDAGAIGRRYRRQDEIGTPFCV
TIDFDSLDDNAVTVRERDTMEQKRMPLEELRGYLAQELIGC
>WP_007236092.1
MSEDRTVWVVDDDRSIRWVMEKALTQAGLLCQSFETAEALLEAITSGAPDVVISDIRMPG
IDGLALLGQLRAAYPELPVIITTAHSDLDSAVASYEEGAFEYLPKPFDVDEIVATVLRTP
TMRKERKAPVTELPDKPTEIIGNAPAMQEVFRAIGRLAHSQITVLINGESGTGKELVARA
LHRHSPRKDGPFIALNMAAIPRELMESELFGHEKGSFTGATARRAGRFEQADSGTLFLDE
IGDMPAETQTRLLRVLADGEFFRVGGAAPVKADVRIIAATHQNLETLVANGQFREDLFHR
LNVIRIHLPRLADRQEDIPKLMQFFLGKAAQELGVEGKVLSTSASRYLCQLPWPGNVRQL
ENTCRWLTVMAAGREIHPSDLPPELLEPAQSQRVDNATTWQDTLATWAQQRLAAGESNVL
RKALPEFERIMIAAALTHTGGKRAEAAETLGWGRNTLTRKIKELEEDGTPAKGA
>WP_007226588.1
MATYRNFALVAAIVIVAFYQTLVDLMGNWLKFDESQSHGLIIIALFIHLFTGQLKQLPSP
PATPNWLGLMGLSASSLVWCLAAMLNIEAIEQLILLPILFFLCWSSLGLRSTVTLTPSIA
LLIFAIPIWDYLTPTLIDASSYVVMTLIQLSSITAFIDGNSIYLPHGRIDIADGCSGLRY
FIIAIALAYYLILTSKTTHLTKVKVLGIAIALGLFTNWLRIYIIIMVAHFTEMESSLVKD
HELFGWFLFFIVCLPLVYFARSLPHYEPTTPSATSAGVTKLTLVVSVIALTSGPLLYQLM
NTKVTAPNLGNWQQLGYQQLSSPTNGPFQLPPSNLNLRKQSGATLRDVAIHWQNSQDSDL
VPYIANSLNRDYWTQLQTSTLQTPKQQSLQLNLYNRKATNQYRCTVSWYRVGGMETTHYN
IAKLLQIPALLSQHNQFSAAVISINSETANCDPHQQQLIDAAIETHNDIVQLTGLTEQ
