# Supplementary material for: BaPreS: a software tool for predicting bacteriocins using an optimal set of features
Source: BMC Bioinformatics. 2023 Aug 17;24:313. doi: 10.1186/s12859-023-05330-z (PMC10433575; doi:10.1186/s12859-023-05330-z)

Figure S1. Illustrating the distribution of the samples/features in the training dataset using principal componet analysis (PCA). Only first two principal components of the PCA were shown in the plot to depict the distribution of the features in a two-dimensional space, where each point in the space represents a sample. The first and second principal components are the linear combinations of the features that capture the highest and second highest amount of variations in the training dataset.

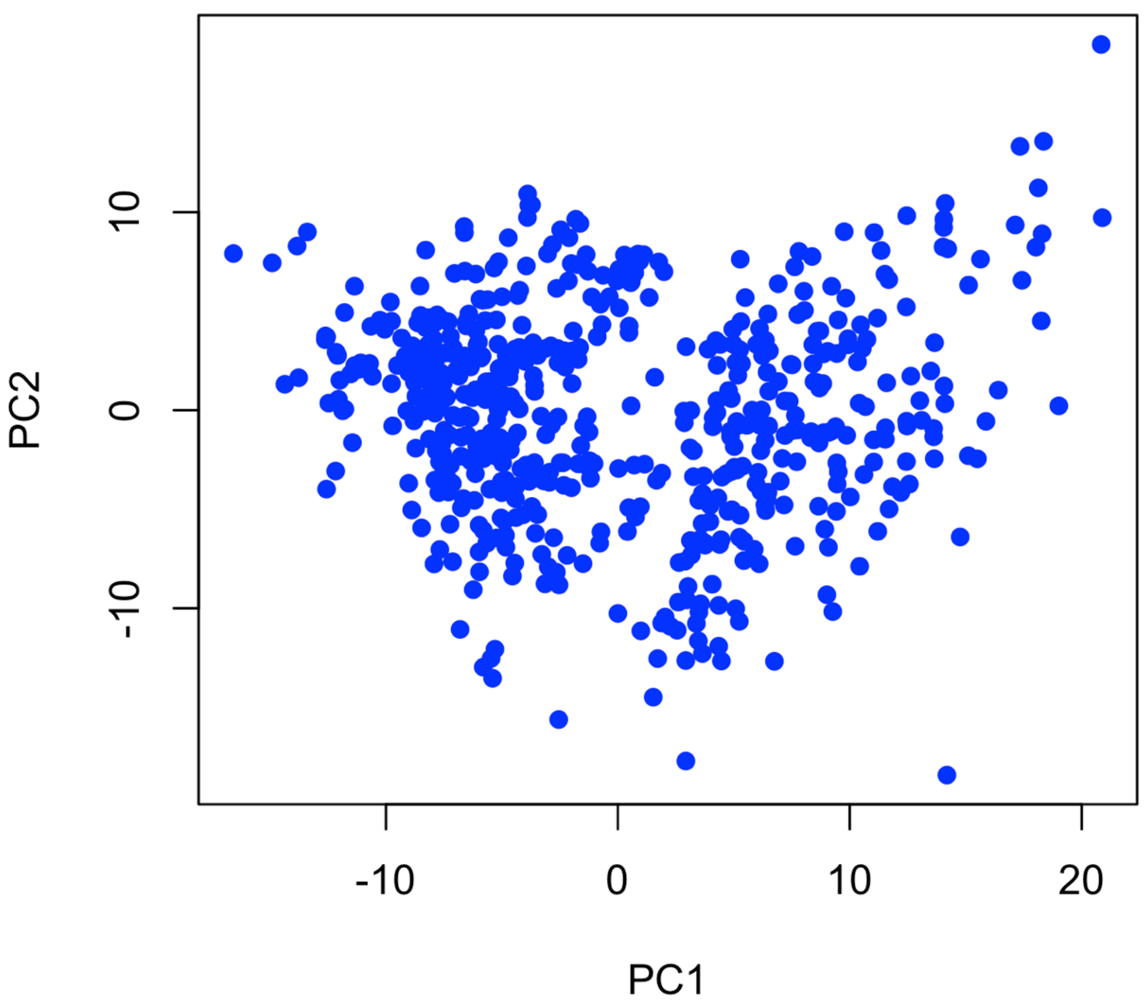

Supplement: Supplementary file 2 — Additional file 2 Principal component analysis of the training dataset. [file 12859_2023_5330_MOESM2_ESM.pdf]
