# Supplementary material for: BaPreS: a software tool for predicting bacteriocins using an optimal set of features
Source: BMC Bioinformatics. 2023 Aug 17;24:313. doi: 10.1186/s12859-023-05330-z (PMC10433575; doi:10.1186/s12859-023-05330-z)
Supplement: Supplementary file 3 — Additional file 3 Testing dataset composed of known bacteriocin and non-bacteriocin protein sequences. [file 12859_2023_5330_MOESM3_ESM.pdf]

**Testing dataset**

**Bacteriocin**
**---------------------------------------**
>BAC005
TPVVNPPFLQQT
>BAC008
DIDITGCSACKYAAG
>BAC013
CVQSCSFGPLTWSCDGNTK
>BAC052
MNFLKNGIAKWMTGAELQAYKKKYGCLPWEKISC
>BAC076
ATYYGNGLYCNKQKCWVDWNKASREIGKIIVNGNVQHGPWAPR
>BAC110
GGAPATSANAAGAAAIVGALAGIPGGPLGVVVGAVSAGLTTAIGSTVGSGSASSSAGGGS
>BAC112
NKWGNAVIGAATGATRGVSWCRGFGPWGMTACALGGAAIGGYLGYKSN
>BAC116
KSYGNGVQCNKKKCWVDWGSAISTIGNNSAANWATGGAAGWKS
>BAC127
AVNDYEPGSMVITHVQGGGRDIIQYIPARSSYGTPPFVPPGPSPYVGTGMQEYRKLRSTL
DKSHSELKKNLKNETLKEVDELKSEAGLPGKAVSANDIRDEKSIVDALMDAKAKSLKAIE
DRPANLYTASDFPQKSESMYQSQLLASRKFYGEFLDRHMSELAKAYSADIYKAQIAILKQ
TSQELENKARSLEAEAQRAAAEVEADYKARKANVEKKVQSELDQAGNALPQLTNPTPEQW
LERATQLVTQAIANK
>BAC149
MSKRDCNLMKACCAGQAVTYAIHSLLNRLGGDSSDPAGCNDIVRKYCK
>BAC177
DWTCWSCLVCAACSVELLNLVTAATGASTAS
>BAC213
KCPWWNLSCHLGNDGKICTYSHECTAGCNA
>BAC215
VLSIVACSSGCGSGKTAASCVETCGNRCFTNVGSLC
>BAC217
GWVACVGACGTVCLASGGVGTEFAAASYFL
>BAC220
MGAIAKLVAKFGWPFIKKFYKQIMQFIGQGWTIDQIEKWLKRH
>BAC221
KRKKHRCRVYNNGMPTGMYRWC
>BAC226
ITSFIGCTPGCGKTGSFNSFCC
>BAC227
ANLGNYTSQCYSSQCYSSKCYSDSCYSSNCYTGRHMCGYTHGYSC
>BAC228
GIGTAQCAYFKALCYSGGSEWLGGYGGCGSTQNNCELARKYC
>ACA04496.1
MNKKNILPQLGQPVIRLTAGQLSSQLAELSEEALGGVDASTSIAPFCSYDGVDASTSIAP
FCSYDGVDASTSIAPFCSYDD
>CAA74348.1
MENKKDLFDLEIKKDNMENNNELEAQSLGPAIKATRQVCPKATRFVTVSCKKSDCQ
>AAK32694.1
MNKELNALTNPIDEKELEQILGGGNGVIKTISHECHMNTWQFIFTCCS
>sp|Q38L35|Q38L35_STRSL
MKNSKDVLNNAIEEVSEKELMEVAGGKKGPGWIATITDDCPNSIFVCC
>AAZ76602.1

MKSNLLKINNVTEVEKDMVTLIKDEDMELAGGSTPACAIGVVGITVAVTGISTACTSRCI
NK
>sp|Q52052|Q52052_9ZZZZ
MENLSVVPSFEELSVEEMEAIQGSGDVQAETTPVCAVAATAAASSAACGWVGGGIFTGVT
VVVSLKHC
>BAD74571.1
MAKLDDFDLDIVVKKQDNIVQPNITSKSLCTPGCITGILMCLTQNSCVSCNSCIRC
>BAB04172.1
MVNSKDLRNPEFRKAQGLQFVDEVNEKELSSLAGSGDVHAQTTWPCATVGVSVALCPTTK
CTSQC
>NP_940772.1
MENSKVMKDIEVANLLEEVQEDELNEVLGAKKKSGVIPTVSHDCHMNSFQFVFTCCS
>ABI99444.1
MNKKNILPQQGQPVIRLTAGQLSSQLAELSEEALGDAGLEASVAACITFCAYDGVEPSCT
LCCTLCAYDGE
>WP_013079673.1
MTSRFQLLRLGKADRLTRGALVGLLLEDITVARYDPM
>YP_142020.1
MATQTIENFNTLDLETLASVEGGGCSWRGAGGATVQGAIGGAFGGNVVLPVVGSVPGYLA
GGVLGGAGGTVAYGATCWWS
>NP_297556.1
MRELTSIEMNNVSGGDLATRIEASIVFGVSAFFAGSIWGGTRGGDGGGILGVGSIAQGVG
MVYGGIVGGIGGLIAGFVLDKNVTYNYAVGFYNSLFNGTFTK
>CAA11804.1
MKNLKEGSYTAVNTDELKSINGGTKYYGNGVYCNSKKCWVDWGQASGCIGQTVVGGWLGG
AIPGKC
>AAL39164.1
MKQYKVLNEKEMKKTIGGESVFSKIGNAVGPAAYWILKGLGNMSDVNQADRINRKKH
>AAU29394.1
ATRSYGNGVYCNDDKCWVNWNEANQQIAGIVISGWASGLAGMGH
>NP_964622.1
MKQFNYLSHKDLAVVVGGRNNWQTNVGGAVGSAMIGATVGGTICGPACAVAGAHYLPILW
TAVTAATGGFGKIRK
>AAL77872.1
MKKFKELKENELTAITGGSFVGYYLGRFLASATHYYGKTVTKGHMHSSTINN
>NP_664144.1
MTTMKELTINDMASISGGNAPGDAVIGGLGGLASGLKFCKLPHPVLTGGCVVGFTVGGAY
LGYTAN
>AAL09346.1
MMKKIEKLTEKEMANIIGGKYYGNGVTCGKHSCSVNWGQAFSCSVSHLANFGHGKC
>AAZ76605.1
MWGRILAFVAKYGTKAVQWAWKNKWFLLSLGEAVFDYIRSIWGG
>CAA75396.1
MDKIIKFQGISDDQLNAVIGGKKKKQSWYAAAGDAIVSFGEGFLNAW
>CAA75397.1
MNNALSFEQQFTDFSTLSDSELESVEGGRNKLAYNMGHYAGKATIFGLAAWALLA
>AAG02567.1
MKISKIEAQARKDFFKKIDTNSNLLNVNGAKCKWWNISCDLGNNGHVCTLSHECQVSCN
>YP_395172.1
MMIFKKLSEKELQKISGGVGIQKCSLGFSSREYLNKITKWIKHH
>ZP_03845684.1
MLYKIIYRSMILMEKFIELSLKEVTAITGGKYYGNGVHCGKYSCTVDWGTAIGNIGNNAA
ANWATGGNAGWNK
>AAY44084.1
MQNTKELSVVELQQILGGKRASFGKCVVGAWGAGAAGLGAGVSGGLWGMAAGGIGRELAY
MGANGCL

>YP_025353.1
MSGGDGKGHNSGAHDSGGSINGTSGKGGPSSGGASDNSGWSSENNPWGGGNSGMIGGSQG
GNGANHGGENTSSNYGKDVSRQIGDAIARKEGINPKIFTGYFIRSDGYLIGITPLVSGDA
FGVNLGLFNNNQNSSSENKGWNGRNGDGIKNSSQGGWKIKTNELTSNQVAAAKSVPEPKN
SKYYKSMREASDEVINSNLNQGHGVGEAARAERDYREKVKNAINDNSPNVLQDAIKFTAD
FYKEVFNAYGEKAEKLAKLLADQAKGKKIRNVEDALKSYEKHKANINKKINAKDREAIAK
ALESMDVEKAAKNISKFSKGLGWVGPAIDITDWFTELYKAVKTDNWRSLYVKTETIAVGL
AATHVTALAFSAVLGGPIGILGYGLIMAGVGALVNETIVDEANKVIGI
>AAN76832.1
METAVAYYKDGVPYDDKGQVIITLLNGTPDGSGSGGGGGKGGSKSESSAAIHATAKWSTA
QLKKTQAEQAARAKAAAEAQAKAKANRDALTQRLKDIVNEALRHNASRTPSATELAHANN
AAMQAEAERLRLAKAEEKARKEAEAAEKAFQEAEQRRKEIEREKAETERQLKLAEAEEKR
LAALSEEAKAVEIAQKKLSAAQSEVVKMDGEIKTLNSRLSSSIHARDAEMKTLAGKRNEL
AQASAKYKELDELVKKLSPRANDPLQNRPFFEATRRRVGAGKIREEKQKQVTASETRINR
INADITQIQKAISQVSNNRNAGIARVHEAEENLKKAQNNLLNSQIKDAVDATVSFYQTLT
EKYGEKYSKMAQELADKSKGKKIGNVNEALAAFEKYKDVLNKKFSKADRDAIFNALASVK
YDDWAKHLDQFAKYLKITGHVSFGYDVVSDILKIKDTGDWKPLFLTLEKKAADAGVSYVV
ALLFSLLAGTTLGIWGIAIVTGILCSYIDKNKLNTINEVLGI
>CAA33859.1
RFAHDPMAGGHRMWQMAGLKAQRAQTDVNNKQAAFDAAAKEKADADAALSTAMESRKKKE
DNKRDAEGKLNDELAKNKGKIPGLKIDQKIRGQMPERGWTEDDIKNTVSNGATGTSFDKR
SPKKTPPDYLGRNDPATVYGSPGKYVVVNDRTGEVTQISDKTDPGWVDDSRIQWGNKNDQ
>prf||1615299A
RFAHDPMAGGHRMWQMAGLKAQRAQTDVNNKQAAFDAAAKEKSDADAALSSAMESRKKKE
DKKRSAENKLNEEKNKPRKGVKDYGHDYHPDPKTEDIKGLGELKEGKPKTPKQGGGGKRA
RWYGDKGRKIYEWDSQHGELEGYRASDGQHLGSFEPKTGNQLKGPDPKRNIKKYL
>prf||1814449A
MSGGDGRGHNSGAHNTGGNINGGPTGLGGNGGASDGSGWSSENNPWGGGSGSGVHWGGGS
GHGNGGGNSNSGGGSNSSVAAPMAFGFPALAAPGAGTLGISVSGEALSAAIADIFAALKG
PFKFSAWGIALYGILPSEIAKDDPNMMSKIVTSLPAETVTNVQVSTLPLDQATVSVTKRV
TDVVKDTRQHIAVVAGVPMSVPVVNAKPTRTPGVFHASFPGVPSLTVSTVKGLPVSTTLP
RGITEDKGRTAVPAGFTFGGGSHEAVIRFPKESGQKPVYVSVTDVLTPAQVKQRQDEEKR
LQQEWNDAHPVEVAERNYEQARAELNQANKDVARNQERQAKAVQVYNSRKSELDAANKTL
ADAKAFIKQFERFAREPMAAGHRMWQMAGLKAQRAQTDVNNKKAAFDAAAKEKSDADVAL
SSALERRKQKENKEKDAKAKLDKESKRNKPGKATGKGKPVNNKWLNNAGKDLGSPVPDRI
ANKLRDKEFKSFDDFRKKFWEEVSKDPELSKQFSRNNNDRMKVGKAPKTRTQDVSGKRTS
FELHHEKPISQNGGVYDMDNISVVTPKRHIDIHRGK
>YP_194414.1
MVGSITPKLVYRLNGMHHVVAQVGAVNGDHVFALQLLHSAHDVLVYRKHKGLTKDINYTN
PHLVMTGFGHTQTWVPANDNDEYFVGAKPNSGNWTTQIARVKYPRLLSENYTSNTQLPRL
SHLNRVTDVPYDGHNHLHRVEASVSPNGKYFMIASIWNNGSGHFGLFDLDEVNQKLDENG
TTNTPITDLHCLSAFHIDNFDNPSVAPDEEEPTMIDSVQGYAIDNDKNIYISNQLSPKIN
HETGEVTTWARKIVKFPWGETDSNNWQVAMIDGIDLPDRYSEVESIHVNAPDDIYLTVAY
HQKIVKGDEYALRTLENQIFHIDNL
>AAT85003.1
MEMVDQKINAQVLSGVNDDISEMKSLTTLRKRVVTDGEVVSKSQNAFRLAGGKTGVILRN
DGNDFYALVTPEDQAQDGQWNTLRPLSFNLKTGRVSLRNGVDISGGAVVSHDAGISARTT
GPSPIINGQTYSSPSIHTDFTSGNITTQMMMCARVEAGKQDYGLLSYRDWQGSWNELRVR
SNAELDAGQFTKRNSEGWIKAAGNRNVNNDKDRKTNALWIQGAGDLSADFYHYERIGQHH
FLGLHVANGGAQGWYEFRNDGHAYTNGAWNSSSDARMKTQVEKIDNALEKLDCISGYTYL
KQGVTEAGVIAQELEEVLPQAVSKTELTLNDGSVLKDARSININGVVALLIEALKEERQA
RLALEKRLADLEARSGQETE
>AAT90328.1
MAEQKKNALVLNGINDDITELKSLTTLRKRVVSDGEIVSKGVNGFRLAGASTGVILRNDG
KNFNFLTTADGQARDGAFNTLRPFAFSLTTGRVSLRNGVDVSGGAFISHNAGITAQTTGP
DPLINGQTYRAPDIHTDFTTGKKTTTMLMGSRIVTGQEDYGLISYRDMKGSWNELHLKPN

AELSVGQLTKRNTEGWYKAAGVRKVNNGKDNKTNALWIQGAGDLSADFYHYERIGQHHLL
GLHVANGGAQGWYEFRNDGHAYTNGAWNSSSDARMKTDIEKIDNALDRLDRIGGYTYLKQ
GKPEAGVIAQEVETVLPQAVTQTALTLNDGSVLEDARAVNINGVVALLVEALREEKQARL
ALEARLQVLEGTDAVHS
>ZP_00378412.1
MNNLYRDLAPVTDSAWAEIEEEARRTFKRNIAGRRIVDVEGPTGFETSSVGTGHIRTLGS
TGGDISIKQRISQEFIELRVPFTVTRQAIDDVERGSGDSDWQPVKDAATTIAMAEDSAIL
HGLDSAGIGGIVPGSSNTPVAIPDAVEDFADSVAQALSGLRKAGVDGPYSLLLSSEEYTK
VSESTDHGYPVRDHLSRLLGDGEIIWAPALEGALLVSVRGGDYELHLGQDLSIGYHSHNG
DSVELYLQETFGFLALTDESSVPLHR
>YP_121242.1
MNNLHRELAPITSEAWAAIEEEAGRTFKRHIAGRRVVDVAGPHGVDFSAVGLGRTTGIAA
PDEGVQARQRVVAPLVELRVPFTLSREELDNVERGAKDTDLDAVKEAARRIAFAEDRAIF
EGYPAAGITGIRAAGSNAPITVPDDARLVPEAITQALTALRLAGVDGPYSVLLSAELYTE
VSETSDHGYPIRTHIERLIPDGEIIWAPAIDGAFVLTTRGGDYELTLGQDVSIGYLSHDA
DTVRLYFQQTMQFLVHTAEAAVALRR

**Non-bacteriocin**
**-----------------------------------------------------------------------**
>WP_177374305.1
MPLDPIHAFYCRKDYLSLAQSCKVKSGGICARCGGVFDLNELRPHHKIELTLDNIDDTNI
TLNPDNIEVLCHACHNAVHSRFGNAIGAKRVYLVYGSPYAGKTTYVALVATRNDIVVDLE
RIHAAICVCGQYDKPDATKRIAFNIRDYLLDEIRTATPRRKWQDAYIIGSYPDRIDRDNF
VREYNAELVHIDTPQDACVKRAYEDIKRVAARDAVVGWIADYWRRYNE
>WP_142482129.1
MIEVSTRQDRANFYGSNTWRKLRLKALERDHYECQWCKEQGKVTTINDAILEVDHIKELE
HYPELATDMDNLRTLCKDCHNKRHGRMNYRGEERKKKFDDEWW
>WP_149877315.1
MTELVVVLFYWGDSMLVACSRCGVIHERGDCKIQDGYSERRIKKRGEVERFRSSALWQRK
RKKILDRDKHLCRVCLDGKYVPKAITNQRLEVHHIVPIVENEKLKLADDNLISICAFCHV
LAEKGNVPRDYLFGLVKIPPRGHYVEN
>WP_121704945.1
MVYIRKRHWVTYNSEKCKMYLRNDFQFECAYCGMKERDNVIGEGLFEKDHFVSRQSDVAW
NLDSYGNMVYSCCKCNGTKSDQNIEIILDPCKDDIYGGQHPHIRRLGAENHYKLYGVTPQ
GQQFIDDLKLNSRFYRKMRQTQAQNEEIRREIYQLLDKSSDFQPSGIDRKIEAYLENGTL
IDERSDEFRCGTSKAGEDVYRVLEKLKERDIKYELLFADDDLDVRVEYCGNIYDCEIRVT
DYAGTEKRGPIVKREKKKTWLKTGNVCGVLYYYKEQDIMDLYIYPNEERTEIVKLG
>WP_086414226.1
MSKEKTASRWGGKGVRVGIIACVIVMVAGIVLWMVQLTGGMIQTGMRNLDAWGLYLTLFM
FFVGLSAGGLIISSIPNAFGMKGFGDISKVAIWSSVCCTCMAIGFVVVDLGGPLRLWELF
VYSNLSSPLMWDILVLSIYLVLSLVYLWAYVRYEQGRMKHTGIRFVSAVALIVAILVHSV
TAWIFSLSPAHEFWHTALMAPWFVASALDCGTALVLIVVIVLRKVGYLELDQHNIVNLAK
MLAVFVCVDLYFFACDLLTSGYFGGTDGAEVVATLTTGSIAPFFWIQMAFMALALVILFV
PKLRTNGGVVVASALVIAGVFCKRCQIMLGGFQIANIDFADTANAFTITNWTDGYSLAGY
SGLVYWPEPIEFGVSLGVIALGALFLLLGLRYLPLRQAKRVSE
>WP_160213184.1
MYGPLIIAYLFFGGTAAGAMLVMAWWSLRFYRKANRPTSRMARAFAAMQQRVYPIGFVLL
LVSMLCLLGDMNYLERAFLVFTRPHPTPITFGAYALAAEMVLAAALSVANILQPLFFTGK
VRRFLEILTVPCSVLLMVYTGVYLFSIMGVPLWNNPAIIPLFFCSSLSSGISAVLLVDYF
ADGSTLLLRAAKPLQKAHMTCIAAEAIVAIAYGASLALDPAAEASLSLLTSPGIAPVLLI
GFAGFGMAVPFCMEGYTLARKECRTIPVSDFVCLVGGFCLRWCVIMCATH
>WP_152931844.1
MLAYLIGLVVTSTLIFIFSEEKVTYRLFAAAITGLTWPLSLIPSIISLMIRKSD
>WP_099730807.1
MIFAPVIRRAAYAQAPRSADLALQRFLMGALAQPAAAPAAGCTVTQDEKATTLQLDVPGL
AREQLSISIEGQVVQVQSVEGAPRKVQRAWELPTEIDASASTAKLENGVLTLTLVRLEPV

SKATTLTIH
>WP_048781921.1
MKASTLRKERKAQSIKSEIIGEVLNAVTHGIGVALAITALVLLLMKAVAVNNTTQIIAFS
VYGASLILLFLASTLYHSFKFTKAAKVFQRIDHSSIYLLIAGTYTPFCLIGIGGQQGFIF
CIAIWVFAIGGVIIEAFFLEKFSKISVFLYLAMGWVSIFTLKPLYESMGWGGILYLFLGG
LSYSLGTIFYKRKYHNFYHVVWHLFVLAGAIFMFLAVFKYL
>WP_005865615.1
MLTAMVIVFLVGYLMIALEHPLKINKAGTALLIGTILWVMYTYAAPFFIPRASAEEFSLF
LESFPSLGSLTFKEQCTRFVVEHQVLDSIGEIAETLIFLIGAMITVELIDAHGGFMFITN
HITTKKKKKLLALIAVITFFMSAVLDNLTTSIVMIMLIRKLLGNYKERWVFGSIIIIAAN
SGGAWSPIGDVTTIMLWVRGNISTSSTIPHLILPSIVSALIPVLIAMRFLHGNVTPPNAF
SQMEADNELLKKLKDKEKLSILIIGVLCLLFVPVFKTVTHLPPFMGILMGVGILWFYTEM
LYARKPIDEDLKLRLSKVVHRIDGATLLFFLGILLAVDALRCSGVLSDFAFWLDDTVGNV
YAVNLIIGALSSIVDNVPLVAGAIGMYPVATDAMVAAATDPAYLANFMQDGVFWQFLAYC
AGVGGSMLIIGSAAGVVVMGLERINFIWYLKNISLLALAGYLSGAVVYILQNLIL
>WP_169170392.1
MIFAPVVRRAAYATRLPMSDLALQRFLRAALARPAAAPGCSAAQDEKAITLQLDVPGLAR
EQLDITIDGAVVRVRSVDGAPRQVQRAWELPEAIDAAASGAKLEHGVLTLTLAKLAPVNR
ATHLTIQ
>WP_110511594.1
MAHYISLFVRAVFVENMALAFFLGMCTFLAVSKKVSTAFGLGVAVTVVLGISVPVNNLIY
NLVLRDGALVEGVDLSFLNFITFIGVIAALVQILEMILDKYFPSLYNALGIFLPLIAVNC
AIFGGVSFMVQRDYNFPESIVYGFGSGIGWMLAIVAMAGIREKMKYANVPAGLRGLGITF
ITTGLMALGFMSFSGVQL
>WP_116624776.1
MFQTLFSSSDVTSTALSVATVATLATAVLTMLGLSWTSERWRVPVALSAVALLASGLVYQ
SALNLWLTGHQLTPATRYVAWFVVQPLQICSVFFFARISGAVPSGVFWRTGAAAILMVLS
RYLGDAQIFNPTLGVLLSIAFWLYILGEMYFGAMAEVVRKSSRPIRLGYFWVRLIMTIGW
AIYPILHFVDVVIGAGHVPSVIVLYTVADLVNLIAVSLIVLAVAGEERF
>WP_140455306.1
MRTSPTRLLGTGLLTALLASLCCIAPLLALVGGVTGAISAFGWVEPFRPYLAGVTVAVLA
LAWYQRLKAGKSAAACACEGEASPTFWKSNKFLLAVSCVALLLLAFPEYAGAFYRQQPVA
KAASVQTDFTQSVKLQVKGMTCTGCEAHVNQEIGKLAGVFSVSTSYEKGNAIIKYDSTKV
KPMQILQAAKKTGYTVAIEDKKP
>WP_168247034.1
MPITKGHGNPTWTREETILALDLLYLHGKPVDRKHQDVSQLSEFLRRVDIHPAQSRTEKF
RNPDGVALKLQNLFSAVEPGRGLTYSKTDLEIVTAFPNSRKSELAEIARLLRSSLLTHEL
VEEHVDEEEVFIEGRWLTSRHRYRDIRLRKRLLQSLPKLCCEICDFSPPSLSRSIQESFF
EAHHTIPISAAEGSVATKVLDMALLCASCHRFIHRLIAEEKRWVTPAEARDYLTGKRNDK
LEDRS
>WP_120447158.1
MGLLGGFFNDLQKVVNDSVGNDYRKIYFSAHPEEQQECACCGATLYRGDSDFTIDHIIPQ
KYNGTNFVTNLQPMCRSCNSRKKDKIDALTLKYSGTMLINEIKNLNRKKEW
>WP_120424551.1
MRKHSKEYSSYMKSDAWSAKREERLQLDGNRCVMCGRPNGLQKDSVTPVLQVHHICYSNL
GNEPMSDLVSICPGCHKKIHKYYRRLRSWEDKEVVARA
>WP_120423357.1
MIKVERKITEKSRRAMDSLERERLKNGSYNTPEVNAALKEMFHGKCYICENKQITSYHIE
HLNPHHGNIELKYSWDNLFLSCAHCNNIKSDKFDPIIDCTKENVEDMIAFRKEGYFGRDE
KLIFDMLDSRIETQNTIKLLQEVYYGSTPQKKMEATILRRTLRKELSDFKEYVREYQESE
DEEKEDLMYLLQMQLSSSSPFAAFKRWLIRDNKDVYPELLEYID
>WP_160581195.1
MKKHQKTLAVLLTAAMLVSLTACSSGGKDSTTAAEAAKTETSAAAGTQAKAETKAEASQE
PVDIAVIVPQKRGDLGFTDSIYKGVEQVMADYADRVNITFTECAGDSSKFESTIYDVCDQ
GPDLIITPSGSGFADLIATKAAHDYSDIKFVLVDNSAAYAGITTDNVAGMSYKQNEATFL
TGALAALLNETGMIGYVAGMSNAVINDFTVGYIQGAQYINPDIKIQISYIGDFADSAKAK

ELAATQIGLGADVVAQVAGTAGLGVLDAAKEAGVWGIGVDADQAAAYKESNPEMSAIIAS
SAMKNGASLLVSIVDRFIGENDLPWGGIESQGLVEGAVEIAPIADNVPDEVKKQISELQE
KVIKGEIEVKSAFTISEEEFNEYVNSCQ
>WP_135856548.1
MATSYDYAPLFRSTVGFDRIFNLLENAQRARSISDWPPYDIIKTGDDSYRISVAVAGFAE
DELDITFQSNLLTVTGKKQDASADEYLHRGIAGRPFEHRFELADHVRVNGADLRNGLLSI
DLVREIPEALKPRKIDIQTSPALQHKVAPAQIEAQKAA
>WP_135901797.1
MSESRIRFRHLQAFLEVARQRSVAKAADFLHVSPPAVTKTLRELEEALGVAVVERDGRGI
RVTRIGEIFLRHAGTAITALRQGVDSVRQDGAINRYPIRIGALPTVSAKVMPHAMSLFLK
ENTSAAIKIVTGENAVLLEQLRTGALDLVVGRLAAPENMTGFFFEHLYSEQVLFVVRSGH
PLLELGADIFARLDAFPVLMPTRESVIRPFVDRLFITNGMTAPATEIETVSDSFGRAFLR
QSDAVWIISAGVVANELGSGAFVALPVDTEETKGPVGLTMRTDTAPSPAFSILLQTIREA
ARPRA
>WP_120435514.1
MLFIWESWHFWLFFVLGACFYYRRNEAEYE
>WP_120446042.1
MKKLLEEIEKNQELKAKIEELDKNPKSTPKDYIQAAAEYGIEIKEEDFKTTRGELSDDEL
DAVAGGKVCSCFVGGGGEGGRRDKICACVAFGAGEDNYADTLRCFCPLAGSGDTHDH
>WP_007225590.1
MDKLIRDSKAAFIIAMALTIWVCAPGAALAGPAYDTYGTVTFDGIKTDVNEYTGGVSSGS
LDLQWFNDHESKNFRYADNVTNALLWEINESSDSPTVWSLNVFFEVPTDARRMIWEDGCT
WIKGGIEGTSCDGLKGLPNGEAILDAYADGSHHFSSKKESKKESKKESKKGSKKESKKES
KKESKKESKKESKKESKKESKKESKKESKKDHEKHSQEGKKEAKMSYSTQTGSEEFSIGE
GEAANNWFGLQKWQDEDENVKDDGSWLTSREYLIENELCDTTFCDAWDSSFSVELLFLFN
TQAGAQNKIKSLTDESVNYAMRLHLSDEANGIDSVTVPEPGPGILLILGLAGLGFARRKA
Q
>WP_140972653.1
MNAYIREPVNAFTHLGGAVLSFIALLAMIVKVSVKMPSFASITAVILFGIGMMVLYTASA
VYHSVVASERVIYFFRKLDHSMIFILIAGTYAPFCLITLHSASGLLLFCLVYATAICGIV
FKMFWFSCPRWLSTAIYITMGWLIVLFFAPLAANLSTGGMVLLVLGGILYTIGGFIYGTK
PKWLEFKYMGHHEIFHVFVLLGSLAHFLSVYCYVI
>WP_169252559.1
MPDSVPTGLIAILRGVRSDEVLDIAEGIVDAGFSAIEVPLNSPDPLASISALVEKFGDSV
EIGAGTVLTADQVRECRQAGARIIVAPDTDRDVITTALELGLTPYPGAATPTEAFAAVKA
GATNVKLFPSSAVGISGMKAWREVLPSGTELFPVGGVGADNAAEWRKAGAAGLGLGSSLY
RRGDRPDDVRTQAQAIASAWAQSI
>WP_169251584.1
MLAVLIGACVGLGVLLWSGSTRRRLQSLLGEGRTDPTGTEAATPEAGPAESGTEPAVADD
QLAFDLDLVAICLTSGLPIPVALTLTAEATDDRSDLQRIARAMTIGGRRLADDDRLLPVL
EVFEFSEHTGVGPAPLIESVAEELRASSRRRRQEAAASLGVQLVLPLGVCILPAFLLLSV
VPVVISLLTDLTTVFF
>WP_169253902.1
MNTDEDVRAAIASLLRIGEPGDGLLKRLVDGIGPVAAQGIIMAVGRGETTAHEAVHGLTV
TGTEAAEHGQMPEAIDRWAVRAGDVETRGDDLDKIARIGGRLVIPDDDEWPRMLDDLGPA
APLGLWVRGAASLSTVLARAVAVVGARAASSYGTKCASDLAWDLAARGITVVSGGAFGID
AAAHRAAIAREAPTVAFMAGGVDRFYPAANADLFEQILSTGAIVSETAPGMTPMRHRFLL
RNRLIAASAQVSVIVEAGWRSGALNTARHALELSRQVAAFPGSVYSASSTGAHKLVREHE
AELVTCSDDVIALMDDETPALFDATAAGAAAENGERPPPPDPREALDEREKICLNALTVS
KPLDVGTIASRAGLTIADALNSLTTLDLAGMAERRDTGWVKLRTSRG
>WP_169253261.1
MNIRRMTVAAVAAVALALTGCGSDGGSGGGGETTDDLTLGTGGTSGTYYPLGGELASIFE
DNVDGVTVNYVESGASAENLGKIYQGEWQLGFTQSDTANTAVNGELEDLDGTKIDNVGWL
ASLYPEAAHIIVREDSGIESVEDLKGKKIAVGDAGSGTRAISDAILDAAGIGESDYTPEI
TDFGASTDMLADKQIDATIFVVGTPVAGLTQLAATTDVKLLGLDDDTTKTIEEGSGAESY
DIPADAYDFLDEDVPTVSVFASLVASTDQVSEDTAYNLTKALFEHTDDITLDVGKLITKD

SAMLGVGDVPLHPGAQKYFEEEGIELP
>WP_040823762.1
MKIFQRVSLRLSLALMCTALFAGTVAAESTASMPLVSPGPHEVVEKTTQQVMEVITSAKG
YYATDPQRFYSEIESVLEDVIDFDGFSRGVMGQYASKKMYVSLETDEEKSAFKERMRRFS
ATFRNGLVQTYAKGLLAFNGNRIDVLPPIESKDLSGTDSVTVTQHIFGEAEKPFVIQYKL
RPNRAGEWKLRNVTIEAINLGIVYRGQFNSAVRLYDGDIDKVIDNWSVDPTGSAKSS
>WP_007235350.1
MQRIDVYWRDIPAQVLIKRGRDRGKHLLSHRFQAAIDRAAMKAGKGGSDAYLEEWRRVTT
SIEAEGSVKDIAQEFGEQIEAQYSDDDIARLVAQKGFDEALT
>WP_007229782.1
MTTISLFPLSGVLLPHGKVPLQIFEQRYIDLVRSSMKTGDPFGIVWIRRGSEVAGRGRAS
SELGDWGTLARIVDWDQLPNGLLGITIQGEGRFDLYETETQSNGLVLGEVVYRDNPASVS
MEAKWQPMLDVLQSLESHPHVQQMGLQLDYGDAWNVAWALIQLLPLEEYLKYELLGLDAI
DEVMSELDLILNQISGED
>WP_007227784.1
MEKVIVCWRDVPAQVIIKHRRKRATVELSERFQKAIDKAAMRAGKADSDAYMEDWCRRSS
PHAGVGSLEDIAKNVADSIESDYSDDVLSKMVADVGYRVR
>WP_007228934.1
MAMDRPQVLIVDDDPRVCRLIQNMACSEQFEYTDIVDPRRLKTVYEDLLPEKIFLDLSMP
GMDGIEALTFLKDSGSTSHICLISGWSEKVLRSSCALGKKMGLNMSPPIHKPFRATEIRA
FLKADQFTHTSVPVSPILPRSRKFKEELKHAIYNVGEIQPFYQPIVDLKTGEVDSLEALC
RWHHPTRGILCPGDFLPLVEKYGLMKDLTYSLLKIILEDMACWDSMASRPNVSINLFPEL
LEEQSLPDVFMAHMKSAGIDPSRITIEITEQSNYGDSVQMMNVISRLRIAGFGLSLDDFG
TGFASMEKVKEIPFTELKIDRSFVSDLLHDPDAKAIVKSSISLAQEIGIPTVAEGIENRE
TLSWLISNGCTRGQGYLLCPPGDFDKSILLAQSSTNHKYEIGEFDSGDVSSSTDTVSTKE
CQTTATLTVTPDIT
>WP_007227234.1
MTGTILLVEDNELNRDMLIRRLVRAGKEVVSAADGQQALDLMRSEKPAVVLMDMNLPILN
GWTACRQARADDTIKHIPIIALTAHASDADRLNALEAGCDDYATKPVDFPGLLIKIEKLT
GNC
>WP_007225255.1
MTLILLVEDNDMNRDMLSRRLQRRQYRVTTANNGAIAVEKATLEKPDLILMDMELPIKDG
WTASREIKATLDTPIIALTAHALSGDRDKALAAGCDDYTTKPINFDHLVAMIEQYLGKKN
N
>WP_007226686.1
MQPPSNHPPLILASSSAYRRQLLLKLNLSFDCVNPCIDETAGSNETADQLVARLAREKAL
AVIHSHPAHLIIASDQVAVLDGVIMTKPGDHGSAIAQLRQCSDKKVIFYTGLTLLNSSTG
RLQNAVEPFSVYFRKLDATTIERYLANEKPYDCAGSFKVEGLGITLFKKLEGDDPNSLIG
LPLIQLTSMLANEGILRP
>WP_007233776.1
MTTHKVLIVDDELPIRDMLRMALETAGYECLEAETIDAAYHQIVDDRPDIVLLDWMLPGG
SGIELLRRIKRAEMTQDLPVIMLTAKAAEHNVIQGLDVGADDYITKPFALRELLARIKAL
LRRAKSSDDRNLLVVRDLTIDIDSRRAFVGEEALQLGPTEFNLLLFFMSHPERAYTRSQL
LDRVWGANIYVEERTVDVHIRRLRAALDAAEGDYSQLIQTVRGTGYRFSEQGG
>WP_007225159.1
MEEKTILIVDDEAPIREMIRMSLDMAGFNCREAADTREAYRVIADSKPDLVLLDWMLPGG
SGIELLRRLRKEELTADLPVIMLTAKTDEDNKIQGLDVGADDYITKPFAPREMLSRIKAL
LRRTSTGIGGSVIEVQGLKLDISSHRVYIDTRPVDMGPTEFRLLSFFMTHQERAYSRGQL
LDHVWGGNVYVEERTVDVHIRRLRKALESEGGCYNECVQTVRGTGYRFSSKSIPPA
>WP_007226710.1
MTQDTNPPGVPEGFRTLRNSAHAETHVGPFYYKKDDDELTLGFLAGDQHSNAIGGVHGGV
LMFFADYAVVMSAMKGQKENCATISASCDFVSSAHTGEWVEAEATITRRTGSMVFVSGRI
YVGDKTVMTVQSVLKRIIPREK
>WP_040541238.1
MSQRIVIDPNDPVTCPDCSHEFPLVQGISHHLIERYEEEYDQKLAEEREALEARAVRTAE
RQLSGRFEEQLGDLTDKLEDAQAEREKAHKKLTKEKARAADQAREEAAEELSDLKQQLGE

KDEKLEDFRKEELALRKAKQVLDQEKRDLELTLQRQLEEQQSALRAELGNEFQLREAELR
KKIDDAHSANEDLKRKLEQGSQQLQGEVLELELEEILSQAFPIDQVDAVSKGVRGADVIQ
TVNLRSGASAGKIVWETKRAENWSNKWVSKLKDDQQSVGGEIGVLVSTAYPANVDEPFTQ
IDGIWLVRPEFAKPLADALRAILIEAFRQRTASSGKNEKMEALYDYVCSAQFAQKVRAVL
DAYAAMRDDLEREKAAMQRLWKKREGQLERITVNVVGICGELQGLSTASLPHLDEIAPIE
VA
>WP_009773675.1
MGKNITVVAAIAVATLLMGACSSGEVIDPAEGNPGADLRAGEAYDPRAFEGESINMLLIE
HPFVNSLRPLIPDFEAATGITVNLEVLNEQQGFDKLQADLSAGVGNYDLFMTDPLHNWQY
SAAGWIEPLDGYVENDAITMPDYNIDDFAPGVLDAGRWNRELLTGLGEGSLWALPVNFES
YNLTYRPSMFEDAGVEVPTTYEDVLDVTESLATSLSGNNYPIVTRFDKYWDLTYLTFGSM
AESYGVNLLNDDGEVDIASDASVEVTDLFIDIIKAGSPQDASAFTWYEVLQGMASGRFAL
ALNEADLFAATYENDAESEIADDVGYALIPEGPEKRAASAWIWQLSMAQASADKGAAWTF
LQWLTSADVLMQTHLAGNMNPVRLSAWEDPELAALVDTWGSEPGQYREVLEGTAEIAAIN
YPPHPELTRALDRWAEAVQQSFFDGNTKANLESAASDIERILLP
>WP_009773511.1
MSPTIVRGMTWEHERGYGSVVKAAEAYRSVAPDVEVQWEYRSLQAFADQDLESLVEQYDL
LVIDHPHIPIAAEEKLFTPLNGRGFDTELATLATQSVGRSHESYKHLGQQWGLALDAAAQ
VAAYRPDLLESPPRNWDEVMALAEEGRVLWPFKPVDAYSSLITIAAGLGEDPMATAGVFL
SEEMLTRAMELLVRLARLVPADNAGFNPIQVADVLAESDIFAYSPLLFGYTNYSRVGYRS
KRVQYTDIPSSTRGVAGSLLGGAGIAVSSRSRVMDAAIAHAFWLASGPVQEGSYYDGGGQ
PGNAVAWESARTNSDSLDFFTGTRATLEGAYMRPRFATYIELQNAVSPFVTSALLGEITI
TELRERLDAGVAEWLVR
>WP_007235958.1
MTIDIDHRRVLIVDDQSTRAHHLIDALGMDAFEFDVANEVPDLQGALGPDSPWDCVLCNA
GLINVSWASVRRAMRNFDVQVPVIVVADEQNVDSMKTALGLGATDFFVKPHARPGLLKRS
IERCVNHRYLQRELKASKEDVERSNTELRHSLRVLEQDQQAGRQVQRALLPSGALHQGDY
WFSHTIVPSLYLSGDFTDYFSVGEDQIAFFLADVSGHGSSSAFATVLLKNLFARKRSDFL
RRGDHSVVSPKDMLELANNELLELAINKYATMIVGVLNFKSHQLTYSIAGHLPHPVLLDE
NSVRYLEGEGPPVGLMRDARYTQHEVVLPEHFVLALLSDGILELLGNGNLIEKEASLLSL
LEGPLESPRSLATRLGLEAVDPNHLPDDVAALFITRGFS
>WP_007227112.1
MGNCETKVLILEDDPLAASELKDCLAREGLHPSIARSKEHFEKIVEQHEFQLLIVDIGLP
DGSGLDVIREVREQSSVGIIVVSGYTTESDVVAAIELGADDYIKKPISIKELRAKVRRMM
IRTSGNGYSRSIAQPNNNEQKFFGDWHLDLDSHRLFYKINHEVGLTSAEYKILLALLNNC
DQVLSRHSLLNHLQSISSPYDERTIDGLINRVRKKLAIPASYEPVQKVRNAGYMFCETVR
TEHQQTTESPGSGFSLTAEKMTIDKAPSPTLEPPVFSSAPESGSTKLTH
>WP_007224478.1
MSSVEQLIQDSRLWRGKHYRDDHSQQTGNSISSGITQLDQQLHWRGWPLHSSSELLCEHW
GIGELSLLMPLLKKVSHKGRIAWINPPFIPYSPALLSQGITPEKCLLLYPSESDQWWAAE
QVLASSAFAIVMTWFTRQASNATPYRRLQAAAEKGHCLHFHFRPLSSKQQSSPARLRIQL
SSSASQLAVEVLKQPGGWSGQQLVISRPESLLFKQQAVEKWPVYHSSRPSYQVVNGRTDI
PSIIDPQHSDRLNDDQSIIHQPSSSAPTQPH
>WP_007223999.1
MAILALIQNNIDRFSDVTGRILAWLCLLLMLLSCSVVFIRYGLGAGSIALQESVTYLHGT
IFMLGAAYTLRHDGHVRVDIFYRNMSARSKAWVNCGGGIIFLLPLCVYFFISSWGFVQQS
WEFREISSEPGGIPAVFLLKTLIPLMAVNLGLQAFAETLRNLLILIAREDSVQL
>WP_007223681.1
MSDLSPQEIEILHEALDDEYLAWSTYDQVIEDFGEISPFINIREAESRHIEALCTLFNRY
GVPVPPNPWLGRVERYKSIQEACEAGVKAEIANGEMYERLMVATQRRDFLEVLGNLQEAS
QKRHLRAFERCVSRRGSGCGAGRGRGRGNGGRC
>WP_007234293.1
MDLATLLGLLGGLAVVGTAIFYGGAGPTFYNVPSILIVIGGTFMTVMVKFSLKQFLGAFK
VAGRAFSNKSHDPESLIAEIVNLANIGRKEGLLALEKAAISESFLKDGIQMLVDGSNQEV
VKAVMAKDMQQTMDRHNWGERVWRAVGDVAPAMGMIGTLVGLVGMLVNMNDPKAIGPQMA
VALLTTLYGAVLANMVALPIADKLHLRKSNEKLIHQMCIDGVLAIQAGQNPRVIESMLKA

YLDPAHRDKNANSGK
>WP_007236262.1
MSAVLVKELRERTGLGLLECKRALKEADNDIDAAIEALRKSSGMKAAKKAGRIAADGVVT
TRTAEDGSYGVLVEVNSETDFVARDENFLGFVGSVADTLYESRSADIDALKSGSLEQARE
ALVQKIGENIGIRRASLVTAENGVVGSYVHGNNRIAVLVELRGGDQDLARDVAMHVAAVN
PQVVSPADMPEALLEKERDIFTAQAQESGKPAEIIEKMIGGRIKKYLAENSLSEQAFVKD
PDVTVGQLVKAADAEVISFSRFEVGEGIEVDKVDFADEVAAQLKG
>WP_007225796.1
MAAVSASMVKELRDRTGLGMMECKKALVEAGGDIDAAIEEMRKNSGMKAAKKAGRTAAEG
VVTAKVAEDGSYGIVVEVNSETDFAARDESLLAFVATVSEKVFTEKQTDVKALMEGDLNT
AREALVQKIGENISVRRSEVVDSDGVVGSYVHSNNRIAVLVSLTGGDAELARDIAMHVAA
VNPQVVRPEDMPEDVVTQEKNIIKAQPDMEGKPEAIVEKMMIGRINKFLKENSLLEQAFV
KDPEITIGKLAKNAGAEVVSFVRYEVGEGIEKEEIDFAAEVAAQLNG
>WP_007233347.1
MSVATRVRTFFQQARDSLVLSGFAKTDGPMGKIVAGVGALYLIVMIVLAIWWSAAPPAFD
MTTKTQDYSTASGQPLVPGSATTLALIEVIDTLLEKDGGYTHNDLLPPGLFIDNMPNWEY
GVLVQSRDLARALREVLSRSQSQSREDVDLTLAEPRINFQSDSWILPASEREYRSANKYL
KAYLARLPEKGPEGARFYARADNLGFWLGMIEKRLGSLSQRLSASVGQRRLNTDLAGDPT
ASAATIDPEEQEIKTPWSEIDDVFYESRGAAWALIHLLKGAEIDFAGVLEKKNARVSLQQ
IIRELEATQGIVWSPIILNGSGFGLWANHSLVMANYISRANAALIDLRELLAQG
>WP_007230569.1
MSVVDRDLRAAGTEVLSLADPLYPPLLKTIPDPPPVLHVRGNPMLLARPQLAIVGARRAS
AAGLQAAHKLAVAAVRAGLGVTSGLALGVDGAAHRGALSAGGDTVAVMATGIETIYPHRH
EPLGQEIASSGCLVTEFPPGTKPLPYHFPKRNRIISGLSLGVLVVESALPSGSLITATSA
MEQGREVFALPWSISHKGGAGCLSLIRDGAKMVLGIEDILEELDSLFGLQQELSQVSTIP
SPESISEQDCLLLELLGFEVISLDQLVVASGLPVGQVMGELSSLELAGRVNRCPGGYIRS
R
>WP_007226893.1
MEHYLSLFVSAIFIKNMALSLFLGMCTLLALSKKMNAAIGLGIAVVVVLSITVPVNYLIY
TYLLREGALVWLSPEFASVDLSFLGLLSYIGVIAALVQILEMFLDKFVPALYNALGVFLP
LITVNCAILGASLLMVEREHDFGESVVFGVGAGVGWAIAIILLAGIREKMKYSDVPAGLQ
GLGITFITVGLMSLGFMSFGGIDI
>WP_007225006.1
MYKILTLNQISTKGLDKFPREDYEIASEFVTSDAVLVRSHKLQPADIQDSVLAIGRAGAG
VNNIPVDYCTEQGIPVFNTPGANANAVKELIVSALTLGSRGILEGIDYVNTLDDLTDGAA
MSKLLEKEKKRFKGNELSGKTLGVIGLGAIGSMVADTALALGMKVAGYDPALSVDAAWRL
SSEVEKVDNITSLVSRADFITLHLPVLDATRKMINRELLSHLKSGAVLLNFAREEIVDTT
AVVEVLDSGKLSKYIADFPTPELIGKRGAVLTPHIGASTDEAEENCAIMAAVQLKDFLEN
GNIKNSVNFPPLYLERTPQSGSVRLSISNRNVPKILGSILSILADENINVIDMLNKSRED
IAYNLIDLQSSPPEQVLEIMRKIDGVVNVRLIG
>WP_007235663.1
MTALSINLNKIALVRNSRVTTVPNIVSHAEMCISAGADGITVHPRPDQRHIRAQDCFDLQ
SALDVELNIEGNPFTEPRASDQPHVGDYPGFIALVQAISPAQVTLVPDSDQQLTSDHGFD
VARDGKRLEPLIKIFKDLGCRVSLFMDPDPSAMATVASLGADRIELYTESYARAHEVGDF
EVSLAAFQETAEAAFAARLGVNAGHDLNLSNLPDFKVPHLEEVSIGHSFTVDALRWGIAN
TIPRYQQALGKNC
