# Supplementary material for: BaPreS: a software tool for predicting bacteriocins using an optimal set of features
Source: BMC Bioinformatics. 2023 Aug 17;24:313. doi: 10.1186/s12859-023-05330-z (PMC10433575; doi:10.1186/s12859-023-05330-z)
Supplement: Supplementary file 4 — Additional file 4 Confusion matrices of the machine learning models. [file 12859_2023_5330_MOESM4_ESM.pdf]

Confusion matrices:

Below are the confusion matrices (1 = positive/bacteriocin and -1 = negative/non-bacteriocin) for the testing dataset. Tables S1-S4 and Tables S5-S8 are confusion matrices for the MDG feature sets, and *t*-test feature sets, respectively.

Table S1. RFE-MDG-RF (RF model).

		Reference	
		-1	1
Predicted	-1	54	4
	1	2	52

Table S2. RFE-MDG-SVM (RF model).

		Reference	
		-1	1
Predicted	-1	54	4
	1	2	52

Table S3. RFE-MDG-RF (SVM model).

		Reference	
		-1	1
Predicted	-1	52	6
	1	4	50

Table S4. RFE-MDG-SVM (SVM model).

		Reference	
		-1	1
Predicted	-1	52	6
	1	4	50

Table S5. RFE-*t*-test-RF (RF model).

		Reference	
		-1	1
Predicted	-1	54	5
	1	2	51

Table S6. RFE-*t*-test-SVM (RF model).

		Reference	
		-1	1
Predicted	-1	54	6
	1	2	50

Table S7. RFE-*t*-test-RF (SVM model).

		Reference	
		-1	1
Predicted	-1	51	7
	1	5	49

Table S8. RFE-*t*-test-SVM (SVM model).

		Reference	
		-1	1
Predicted	-1	54	3
	1	2	53
