# Supplementary material for: BaPreS: a software tool for predicting bacteriocins using an optimal set of features
Source: BMC Bioinformatics. 2023 Aug 17;24:313. doi: 10.1186/s12859-023-05330-z (PMC10433575; doi:10.1186/s12859-023-05330-z)
Supplement: Supplementary file 5 — Additional file 5 Prediction probability values for the testing dataset. [file 12859_2023_5330_MOESM5_ESM.pdf]

Table S9. Probability values for the testing dataset (1 = positive/bacteriocin and -1 = negative/non-bacteriocin).

Sequence	1	-1
BAC005	0.976913	0.023087
BAC008	0.977036	0.022964
BAC013	0.979409	0.020591
BAC052	0.975501	0.024499
BAC076	0.957989	0.042011
BAC110	0.979507	0.020493
BAC112	0.997717	0.002283
BAC116	0.995387	0.004613
BAC127	0.182473	0.817527
BAC149	0.769546	0.230454
BAC177	0.980366	0.019634
BAC213	0.988856	0.011144
BAC215	0.99301	0.00699
BAC217	0.986664	0.013336
BAC220	0.981434	0.018566
BAC221	0.976822	0.023178
BAC226	0.983233	0.016767
BAC227	0.994985	0.005015
BAC228	0.958991	0.041009
ACA04496.1	0.993733	0.006267
CAA74348.1	0.961206	0.038794
AAK32694.1	0.987638	0.012362
sp|Q38L35|Q38L35_STRSL	0.997067	0.002933
AAZ76602.1	0.993676	0.006324
sp|Q52052|Q52052_9ZZZZ	0.991626	0.008374
BAD74571.1	0.96145	0.03855
BAB04172.1	0.993237	0.006763
NP_940772.1	0.963549	0.036451
ABI99444.1	0.975657	0.024343
WP_013079673.1	0.924749	0.075251
YP_142020.1	0.998746	0.001254
NP_297556.1	0.990276	0.009724
CAA11804.1	0.981162	0.018838
AAL39164.1	0.946792	0.053208
AAU29394.1	0.994471	0.005529
NP_964622.1	0.997849	0.002151

AAL77872.1	0.981334	0.018666
NP_664144.1	0.996951	0.003049
AAL09346.1	0.995312	0.004688
AAZ76605.1	0.905644	0.094356
CAA75396.1	0.985005	0.014995
CAA75397.1	0.990006	0.009994
AAG02567.1	0.980887	0.019113
YP_395172.1	0.983775	0.016225
ZP_03845684.1	0.947162	0.052838
AAY44084.1	0.992205	0.007795
YP_025353.1	0.999745	0.000255
AAN76832.1	0.977548	0.022452
CAA33859.1	0.995892	0.004108
prf||1615299A	0.987902	0.012098
prf||1814449A	0.972508	0.027492
YP_194414.1	0.882225	0.117775
AAT85003.1	0.073011	0.926989
AAT90328.1	0.285482	0.714518
ZP_00378412.1	0.897958	0.102042
YP_121242.1	0.903445	0.096555
WP_177374305.1	0.000823	0.999177
WP_142482129.1	0.051078	0.948922
WP_149877315.1	0.002555	0.997445
WP_121704945.1	0.031917	0.968083
WP_086414226.1	0.00099	0.99901
WP_160213184.1	0.001149	0.998851
WP_152931844.1	0.461289	0.538711
WP_099730807.1	0.398126	0.601874
WP_048781921.1	0.013491	0.986509
WP_005865615.1	0.010191	0.989809
WP_169170392.1	0.302178	0.697822
WP_110511594.1	0.037187	0.962813
WP_116624776.1	0.00865	0.99135
WP_140455306.1	0.001271	0.998729
WP_168247034.1	0.001075	0.998925
WP_120447158.1	0.282495	0.717505
WP_120424551.1	0.021751	0.978249
WP_120423357.1	0.00244	0.99756
WP_160581195.1	0.002487	0.997513

WP_135856548.1	0.024719	0.975281
WP_135901797.1	0.01609	0.98391
WP_120435514.1	0.96087	0.03913
WP_120446042.1	0.21329	0.78671
WP_007225590.1	0.552184	0.447816
WP_140972653.1	0.001344	0.998656
WP_169252559.1	0.051634	0.948366
WP_169251584.1	0.093247	0.906753
WP_169253902.1	0.000775	0.999225
WP_169253261.1	0.244695	0.755305
WP_040823762.1	0.009905	0.990095
WP_007235350.1	0.020842	0.979158
WP_007229782.1	0.095576	0.904424
WP_007227784.1	0.179508	0.820492
WP_007228934.1	0.001568	0.998432
WP_007227234.1	0.002452	0.997548
WP_007225255.1	0.011095	0.988905
WP_007226686.1	0.002058	0.997942
WP_007233776.1	0.00019	0.99981
WP_007225159.1	0.001212	0.998788
WP_007226710.1	0.112318	0.887682
WP_040541238.1	0.41631	0.58369
WP_009773675.1	0.038157	0.961843
WP_009773511.1	0.043609	0.956391
WP_007235958.1	0.001285	0.998715
WP_007227112.1	0.004548	0.995452
WP_007224478.1	0.024766	0.975234
WP_007223999.1	0.000182	0.999818
WP_007223681.1	0.029906	0.970094
WP_007234293.1	0.000517	0.999483
WP_007236262.1	0.019007	0.980993
WP_007225796.1	0.006921	0.993079
WP_007233347.1	0.029956	0.970044
WP_007230569.1	0.001706	0.998294
WP_007226893.1	0.014671	0.985329
WP_007225006.1	0.027897	0.972103
WP_007235663.1	0.001931	0.998069
