# Supplementary material for: BaPreS: a software tool for predicting bacteriocins using an optimal set of features
Source: BMC Bioinformatics. 2023 Aug 17;24:313. doi: 10.1186/s12859-023-05330-z (PMC10433575; doi:10.1186/s12859-023-05330-z)
Supplement: Supplementary file 6 — Additional file 6 List of the predicted bacteriocin sequences. [file 12859_2023_5330_MOESM6_ESM.pdf]

Total number of predicted bacteriocin sequences = 55

BAC005

BAC008

BAC013

BAC052

BAC076

BAC110

BAC112

BAC116

BAC149

BAC177

BAC213

BAC215

BAC217

BAC220

BAC221

BAC226

BAC227

BAC228

ACA04496.1

CAA74348.1

AAK32694.1

sp|Q38L35|Q38L35_STRSL

AAZ76602.1

sp|Q52052|Q52052_9ZZZZ

BAD74571.1

BAB04172.1

NP_940772.1

ABI99444.1

WP_013079673.1

YP_142020.1

NP_297556.1

CAA11804.1

AAL39164.1

AAU29394.1

NP_964622.1

AAL77872.1

NP_664144.1

AAL09346.1

AAZ76605.1

CAA75396.1

CAA75397.1

AAG02567.1

YP_395172.1

ZP_03845684.1

AAY44084.1

YP_025353.1

AAN76832.1

CAA33859.1

prf||1615299A

prf||1814449A

YP_194414.1

ZP_00378412.1

YP_121242.1

WP_120435514.1

WP_007225590.1
